# Characterization of Gut Microbiome Dynamics in Developing Pekin Ducks and Impact of Management System

**DOI:** 10.3389/fmicb.2016.02125

**Published:** 2017-01-04

**Authors:** Aaron A. Best, Amanda L. Porter, Susan M. Fraley, Gregory S. Fraley

**Affiliations:** ^1^Department of Biology, Hope CollegeHolland, MI, USA; ^2^South Crossing Veterinary CenterCaledonia, MI, USA

**Keywords:** pekin duck, duck microbiota, 16S rRNA amplicon sequencing, industry grow-out development, aviary environment, barn environment, bacteroidetes, riemerella

## Abstract

Little to no research has been conducted on the gut microbiome of the Pekin duck, yet over 24.5 million ducks are raised for human consumption each year in the United States alone. Knowledge of the microbiome could lead to an understanding of the effects of growing conditions such as the use of prebiotics, probiotics, and enzymes in feeding practices, the use of antibiotics, and the sources of pathogenic bacteria in diseased ducks. In order to characterize changes in the caecal microbiome that occur as ducks develop through a typical industry grow-out period, a 16S rRNA community analysis of caecal contents collected over a 6-week period was conducted using a next generation sequencing approach. Transitions in the composition of the caecal microbiome occurred throughout the lifespan, with a large shift during days 4 through 10 posthatch. Two major phyla of bacteria were found to be present within the caeca of aviary raised ducks, with the relative abundance of each phylum varying by age of the duck. Proteobacteria is dominant for the first 3 days of age, and Firmicutes increases and dominates beginning at day 4. Barn raised ducks contained a significant population of Bacteroidetes in addition to Proteobacteria and Firmicutes at later developmental time points, though this phylum was absent in aviary raised ducks. Genera containing pathogens of anseriformes most often found in industry settings were either absent or found as normal parts of the caecal microbial populations. The high level differences in phylum abundance highlight the importance of well-designed sampling strategies for microbiome based studies. Results showed clear distinctions between Pekin Duck caecal contents and those of Broiler Chickens and Turkey in a qualitative comparison. These data provide a reference point for studies of the Pekin Duck through industry grow-out ages, provide a foundation for understanding the types of bacteria that promote health, and may lead to improved methods to increase yields and decrease instances of disease in agricultural production processes.

## Introduction

Investigation of microbes associated with host organisms has become an increasingly important approach to better understand the host organisms, the microbial communities, and the interactions that occur between hosts, their microbes, and environment (Gilbert et al., 2016). Collectively, the microbes found in a particular environment can be referred to as a microbiome. The microbiome of the gut of many organisms, in particular mammals, has been found to protect against pathogens, impact digestion, influence immune system function, and affect the health of individuals (e.g., Turnbaugh et al., 2006; Cho and Blaser, 2012; Flint et al., 2012; D'Argenio and Salvatore, 2015; Gilbert et al., 2016). The microbiome and its associated genetic content has been proposed to be an extension of the host organism that readily influences development and normal function and may be heritable (Ley et al., 2008; Funkhouser and Bordenstein, 2013; D'Argenio and Salvatore, 2015; Van Opstal and Bordenstein, 2015). Thus, the microbiome can be indicative of the health state of an individual, potentially linked to the absence or presence of disease, and suggest alterations in diet or treatment of gut related disease (Nicholson et al., 2005; Frank et al., 2007; Reid et al., 2011; Gevers et al., 2014; Lewis et al., 2015). A recent meta-analysis of microbiomes associated with various avian species revealed a dynamic, intestinal microbiome that changes with species of bird, host site of sample acquisition (e.g., crop, caecum), captivity status (wild or domesticated), and potential associations with diet (Waite and Taylor, 2014). Studies of commercial avian species have revealed changes in the microbiome associated with species, age, diet, host site, and commercial environmental conditions (van der Wielen et al., 2002; Lu et al., 2003; Gong et al., 2007; Wei et al., 2013; Choi et al., 2014; Stanley et al., 2014; Vasaï et al., 2014; Roto et al., 2015). The majority of bacteria associated with avian species has been found in the intestinal caeca, where a relatively lower oxygen partial pressure and decreased enzyme and bile salt concentrations create conditions suitable for a variety of bacteria (Gabriel et al., 2006).

It is known that avian intestinal contents are much different than those of monogastric mammals (Pérez de Rozas, 2004), however most research has centered upon the gut microbiome of galliformes, specifically turkeys and broiler chickens. Broiler chicken caeca, analyzed using 16S rRNA clone libraries and Sanger sequencing, are dominated by Firmicutes at all ages. At days 7 through 14 of age, the chicken caecal contents resemble that of the chicken ileum. From day 14 forward, the compositions of the two regions diversify and stabilize, becoming significantly different from each other (Lu et al., 2003). By day 28, 5–10% of the caecal contents is composed of Bacteroidetes. Comparable results were found in 28-day old chickens using pyrosequencing to analyze the V1–V3 region of 16S rRNA (Choi et al., 2014; Stanley et al., 2014). In contrast, the caecal microbiome of 18-week old turkeys is dominated by Bacteroidetes (52%), while Firmicutes composition is 33% (Scupham et al., 2008). A recent study assessing 12–14 week old Pekin (*Anser platyrhynchos*) and Muscovy (*Cairina moschata*) ducks revealed that the caecal microbiomes consists of ~65 and ~50% Bacteroidetes, respectively, similar to the composition of turkeys at older ages (Vasaï et al., 2014). Pathogens are often found in the gut of vulnerable galliformes. These include *Brachyspira*, causing colitis (Neo et al., 2013), *Campylobacter jejuni*, a common food-borne pathogen, and *Clostridium perfringens*, causing necrotic enteritis (Van Immerseel et al., 2004). Often present in broiler chickens and turkey, these pathogens have not been linked to anseriformes used in the food industry, such as the Pekin duck. *Riemerella anatipestifer* is the most common pathogen in anseriformes (Wobeser, 1997), occurring globally in both commercially raised and wild ducks (Brogden, 1989). Other bacterial pathogens commonly associated with commercially raised ducks include *Escherichia coli, Salmonella, Streptococcus* and *Enterococcus*.

To date, very little research has been conducted on the gut microbiome of the Pekin duck, yet over 24.5 million ducks are raised for human consumption each year in the United States alone (AGMRC, 2012). Knowledge of the duck caecal microbiome could lead to a better understanding of the effects of management practices such as the use of prebiotics, probiotics and enzymes in feeding practices, the use of antibiotics, and to a better understanding of the sources of pathogenic bacteria in diseased ducks. This study characterized the microbiome of Pekin ducks over the industry standard 36-day period in which the ducks reach market weight, referred to as the grow-out period; determined if bacterial groups consistent with common anseriform pathogens are part of the endogenous caecal flora of developing ducks; and qualitatively compared the microbiomes of galliformes and anseriformes. Further, we compared the microbiome of ducks raised in a highly controlled aviary environment to a barn environment used in commercial practices to identify differences in microbiome composition related to environmental setting.

## Materials and methods

### Sample collection

The study was conducted in two aviary experiments at Hope College in Holland, Michigan, and in a third barn experiment conducted at Maple Leaf Farms (MLF, Leesburg, IN, USA). A straight run (defined as a roughly equal mix of male and female) of day-old ducklings was obtained from MLF and housed in a controlled aviary setting at Hope College. The ducklings were of the commercial strain developed and utilized for international meat production by MLF. Housing conditions adhered to industry standards for 18:6 light:dark cycle, temperature (~18.5°C), humidity (60–65%), *ad libitum* access to commercial feed (identical feed provided by Maple Leaf Farms, Inc. for all studies) and pin-metered water lines, and pine litter flooring. Flock density was standardized across three pens based on industry standards (~0.16 m^2^/duck). All care and procedures were in concordance with the Guide for the Care and Use of Agricultural Animals in Research and Teaching (McGlone et al., 2010) and approved by the Hope College Animal Care and Use Committee (HCACUC).

*Aviary Study 1*. Sixty-two ducks were used. Ten ducks were sacrificed on days 1, 8, 15, 22, and 36, and 12 ducks on day 29. *Aviary Study 2*. Sixty-one ducks were used in which six ducks were sacrificed daily on days 1–10, excluding day 9, when 7 ducks were sacrificed. A final live weight was determined for each animal when euthanized. Samples of water, feed and bedding were also obtained for Aviary Studies 1 and 2. *Barn Study 3*. In an attempt to approximate the conditions of an actual commercial barn setting, our study was conducted in two research barns owned by Maple Leaf Farms, Inc. (Leesburg, IN USA). Each barn was divided into 4 equal sized pens with 1000 ducks per pen (~ 0.17 m^2^ per duck). The study ran for the duration of a typical grow-out period in the USA, approximately 36 days. After the first study was completed, the experiment was replicated thus providing a final *N* = 16 pens. The ducks used in the study were from the same commercial Pekin strain developed by Maple Leaf Farms, Inc. used for the aviary studies. Ducklings were randomly selected for both barns and placed within hours (hr) of hatch (day 1). After an initial 10-day brooding period in approximately one-third of the pen, they were given access to the entire floor space in each pen. Gut ecology samples were obtained on days 5, 23, and 33. In each pen, 3 apparently healthy ducks (*n* = 12 per barn) were selected at random and immediately euthanized using Fatal Plus (400 mg/kg pentobarbital, intraperitoneal). Pentobarbital is a well-known inhibitor of gastrointestinal motility. The ducks were weighed and the paired caeca of each animal were removed aseptically, and caecal contents were obtained and stored at −80°C until they were processed for microbial DNA analyses. Samples of water were also obtained for Barn Study 3. When the ducks reached targeted commercial weight (~3.5 kg) at 34 days, they were processed at the Maple Leaf Farms processing facility. The Hope College Animal Care and Use Committee approved all studies.

### Bacterial DNA isolation

Caecal contents (300 mg wet weight) and environmental samples (water, bedding, feed) were prepared for total community analysis using the PowerLyzer PowerSoil DNA Isolation Kit (MoBio, Carlsbad, CA, USA) according to the manufacturer's protocol except that MP Biomedical FastPrep24 lysing matrix D tubes with 1.4 mm ceramic spheres were used in place of the MoBio glass bead tubes for sample preparation, resulting in consistently higher DNA yields. DNA was eluted in a final volume of 100 uL of elution buffer according to the manufacturer's protocol. Total community DNA was stored at −20°C.

### Sequencing of 16S rRNA

Total community DNA samples were submitted to the Institute for Genomics and Systems Biology Next Generation Sequencing (IGSB-NGS) Core Facility at Argonne National Laboratory for sequencing of community 16S rRNA genes. Briefly, genomic DNA was amplified using the Earth Microbiome Project barcoded primer set, adapted for the Illumina MiSeq (Caporaso et al., 2012). The V4 region of the 16S rRNA gene (515F-806R) was amplified with region-specific primers that included the Illumina flowcell adapter sequences and unique, 12 base barcode sequences. Each 25 ul PCR reaction contained 12 ul of MoBio PCR Water (Certified DNA-Free), 10 ul of 5 Prime HotMasterMix (1x), 1 ul of Forward Primer (5 uM concentration, 200 pM final), 1 ul Golay Barcode Tagged Reverse Primer (5 uM concentration, 200 pM final), and 1 ul of template DNA. The conditions for PCR were as follows: 94°C for 3 min to denature the DNA, with 35 cycles at 94°C for 45 s; 50°C for 60 s; and 72°C for 90 s, with a final extension of 10 min at 72°C to ensure complete amplification. The PCR amplifications were done in triplicate, and then pooled. Following pooling, amplicons were quantified using PicoGreen (Invitrogen) and a plate reader. Once quantified, different volumes of each of the products were pooled into a single tube so that each amplicon is represented equally. This pool was then cleaned up using an UltraClean® PCR Clean-Up Kit (MoBIO), and then quantified using the Qubit (Invitrogen). After quantification, the molarity of the pool was determined and diluted down to 2 nM, denatured, and then diluted to a final concentration of 6.75 pM with a 10% PhiX spike for sequencing on the Illumina MiSeq.

### Data analysis

The Quantitative Insights into Microbial Ecology (QIIME) software package, version 1.9.1 (Caporaso et al., 2010b) was used to analyze 16S microbial sequencing data. We utilized custom shell scripts to perform “upstream” and “downstream” processing stages as recently described (Navas-Molina et al., 2013). All steps requiring comparison of sequences to a reference database used the GreenGenes database, release 13_8 (DeSantis et al., 2006). For the upstream analysis steps, we performed demultiplexing and quality-filtering for Illumina based sequence reads using default values. Clustering of sequencing reads that passed quality filters into operational taxonomic units (OTUs) was performed through an open-reference strategy at a threshold of 97% identity, using uclust (Edgar, 2010). Taxonomic assignment of representative OTUs was performed using the QIIME rtax workflow in order to take advantage of paired end sequencing reads (Soergel et al., 2012). The rtax settings allowed for inclusion of OTUs identified by non-paired reads (–single_ok option). Chimeric sequences were removed using ChimeraSlayer (Haas et al., 2011). In order to construct a phylogenetic tree of the identified OTUs, sequences were aligned using PyNAST (Caporaso et al., 2010a) against the GreenGenes core set template (DeSantis et al., 2006; McDonald et al., 2012). A phylogenetic tree was constructed using FastTree 2 (Price et al., 2010) within the QIIME workflow. Finally, an OTU table in BIOM format (McDonald et al., 2012) was produced, along with a complete metadata mapping file for use in downstream analysis steps.

Alpha diversity metrics (observed species, phylogenetic distance, Good's coverage, Chao1, and Shannon) were performed on all samples at the maximum depth for each sample to yield summary statistics for the data set using QIIME and are reported in Supplementary Table 1. We performed secondary filtering of OTUs to minimize the effect of very low abundance OTUs, using the recommended value of <0.005% of the total number of sequences (Bokulich et al., 2013) as a conservative threshold for removal of OTUs from further consideration. The filtered BIOM table, phylogenetic tree and metadata sample table were passed to the core diversity analysis workflow (Lozupone and Knight, 2005; Navas-Molina et al., 2013) in QIIME to perform taxa summarization, alpha diversity, beta diversity, and taxon differential distribution analyses. Full output files from taxa summary analyses performed in QIIME are reported in Supplementary Files 1–5. A jackknifed beta diversity analysis (Lozupone et al., 2011) was conducted to assess statistical variation of sample location in principal coordinate analysis (PCoA) plots based on unweighted and weighted UniFrac distances. We used EMPeror (Vázquez-Baeza et al., 2013) to visualize PCoA plots. Following initial evaluation of the data, the BIOM table was variously filtered to focus on particular sample comparisons as described in the Results section; rarefaction for all analyses was to 10,000 reads per sample. Targeted group significance tests were used to compare OTU frequencies amongst combinations of age, gender, and experimental setting as implemented in: QIIME (Kruskal-Wallis, Mann-Whitney U, group and pairwise adonis); IBM SPSS version 23 for Macintosh (repeated measures ANOVA); and the R packages phyloseq (McMurdie and Holmes, 2013) and vegan (Dixon, 2003) (DESeq2, two-way adonis). Sequencing data have been deposited as a combined data set for all three studies in the European Nucleotide Archive (ENA) under the accession number (http://www.ebi.ac.uk/ena/data/view/PRJEB15658).

## Results

Microbial communities associated with caecal contents of Pekin ducks were analyzed in order to understand the structure of and changes associated with the development of ducks through a typical industry growth period. Two studies were conducted in a controlled aviary setting—Aviary Study 1 was designed as a broad overview of the 36-day developmental cycle; Aviary Study 2 was designed to focus on the first 10 days of duck development. These data were compared to Barn Study 3, a parallel study that occurred in a production barn environment (Schenk et al., 2016) in order to examine differences in microbial community structure and developmental changes that could occur in different environments. The microbial community profiles were searched for the presence and abundance of potential anseriform pathogens. Processing of sequencing data and evaluation of taxonomic distribution, alpha diversity, and beta diversity metrics were performed in QIIME. Basic information for samples, including barcodes used, sequencing reads, number of taxa observed, estimates of alpha diversity, and metadata are reported in Supplementary Table 1. Clear shifts in the microbial communities occurred in all three studies associated with the age of the ducks and the environmental setting, whereas significant associations with other factors, such as the sex of the ducks, were not observed.

### Aviary study 1: differences in microbial populations associated with age of the developing duck

In order to assess the microbial populations of ducks throughout a typical industry grow-out period, we collected caecal contents at 7-day intervals through 36 days for analysis via 16S rRNA gene sequencing of total community DNA. Multiple alpha diversity metrics revealed clear differences among microbial populations in ducks of different ages through the grow-out period with respect to richness and diversity. In general, there is a significant increase in the diversity of the microbial populations by all metrics as ducks mature (Supplementary Figure 1A, repeated measures ANOVA inset for each metric). Ducks at day 15 or less have fewer than 80 observed species, whereas ducks at day 22 or older have greater than 115 observed species. Ducks at day 36 had, on average, 143 observed species. All pairwise comparisons of the number of observed species grouped by day were statistically significant (Supplementary Table 2, pairwise *t*-tests, *p* < 0.05) with the exception of day 1 vs. day 15 and day 22 vs. day 29. In fact, all alpha diversity metrics produced statistically indistinguishable values for day 22 vs. day 29. When considering the Shannon diversity metric, which takes into account richness and evenness of species, a pattern of early and late stages in the 36 day grow-out period emerges. This is supported by statistically significant differences between early (Days 1 and 8) and late (Days 15, 22, 29, and 36) age duck samples (Supplementary Table 2, pairwise *t*-tests, *p* < 0.05), and the absence of statistically significant differences between days within the early and late groupings (Supplementary Table 2, pairwise *t*-tests, *p* > 0.05). This suggests that there are early and late stages in the 36-day grow-out period that are distinct from each other.

**Figure 1:**
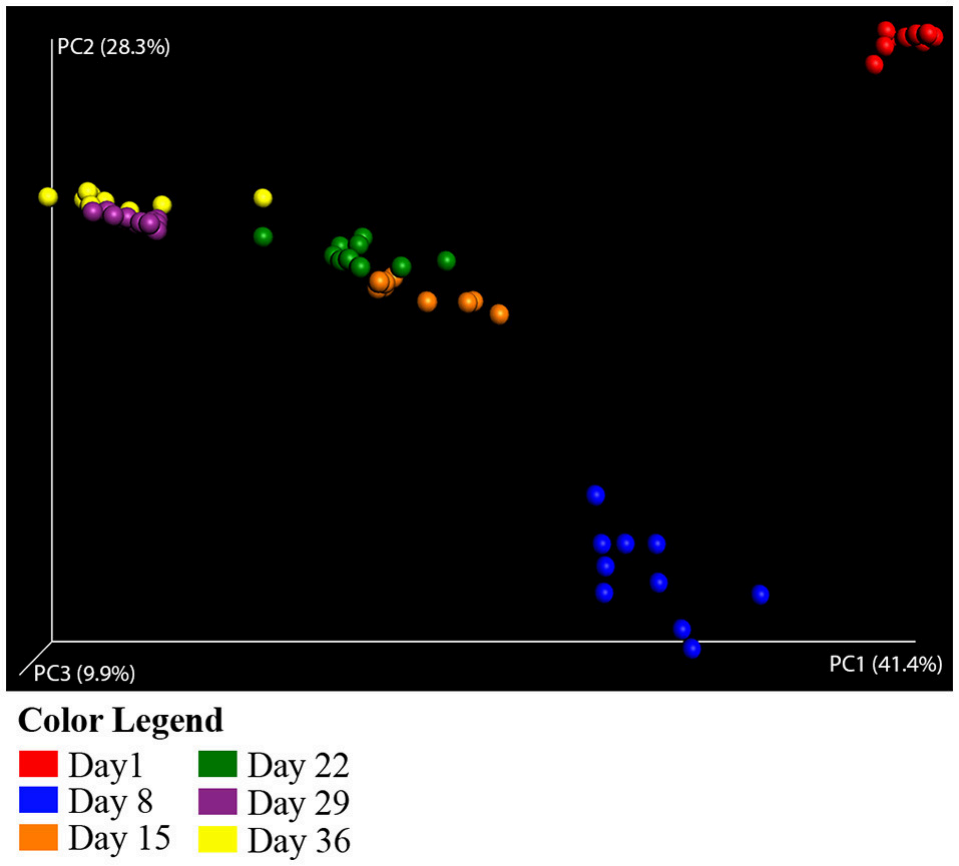
**Principal coordinate analysis of weighted UniFrac distances for Aviary Study 1**. Principal coordinate analysis (PCoA) plots each sample as a point in multidimensional space based on the composition of the bacterial population in each sample. Closeness of two points in the PCoA denotes similar bacterial population composition between the samples. The halo around each point depicts the variation in point location observed in 3 dimensional space after jackknife resampling at the specified rarefaction level. Samples are colored by age of the duck throughout the grow out period—Red, Day 1; Blue, Day 8; Orange, Day 15; Green, Day 22; Purple, Day 29; Yellow, Day 36. Analyses were conducted on data rarefied to 10,000 sequencing reads. Axes are scaled by the percent of variation explained by each principal coordinate. Individual ceacal samples from ducks of the same age form distinct clusters. Halos from statistical resampling are not visible at this scale, an indication of the significance of the separation shown between points.

The structure of the duck caecal microbial populations are distinct and are clearly correlated with the age of the duck based on beta diversity measures. In weighted UniFrac Principal Coordinates Analysis (PCoA), the first principal coordinate (PC1) explained 41% of the variation among samples, with PC2 and PC3 explaining 28 and 10% of the variation, respectively. This analysis shows distinct clustering of individual duck caecal samples associated with age (Figure 1), supports the distinction between early and late age ducks, and suggests a difference between Day 1 and Day 8 ducks. Differences among age groups were shown to be statistically significant in a multivariate ANOVA based on dissimilarities (adonis) test (weighted UniFrac distances, *DF* = 5, 999 simulations, *F* = 41.904, *R*^2^ = 0.79, *p* = 0.001) All pairwise comparisons of age groups were statistically significant (pairwise adonis, weighted UniFrac distances, *DF* = 1, 999 simulations, *F* = 5.48–195.24, *R*^2^ = 0.24–0.91, *p* = 0.001).

### Aviary study 1: major shift in microbial caecal contents occurs early in the 36 day grow-out period

**Figure 2:**
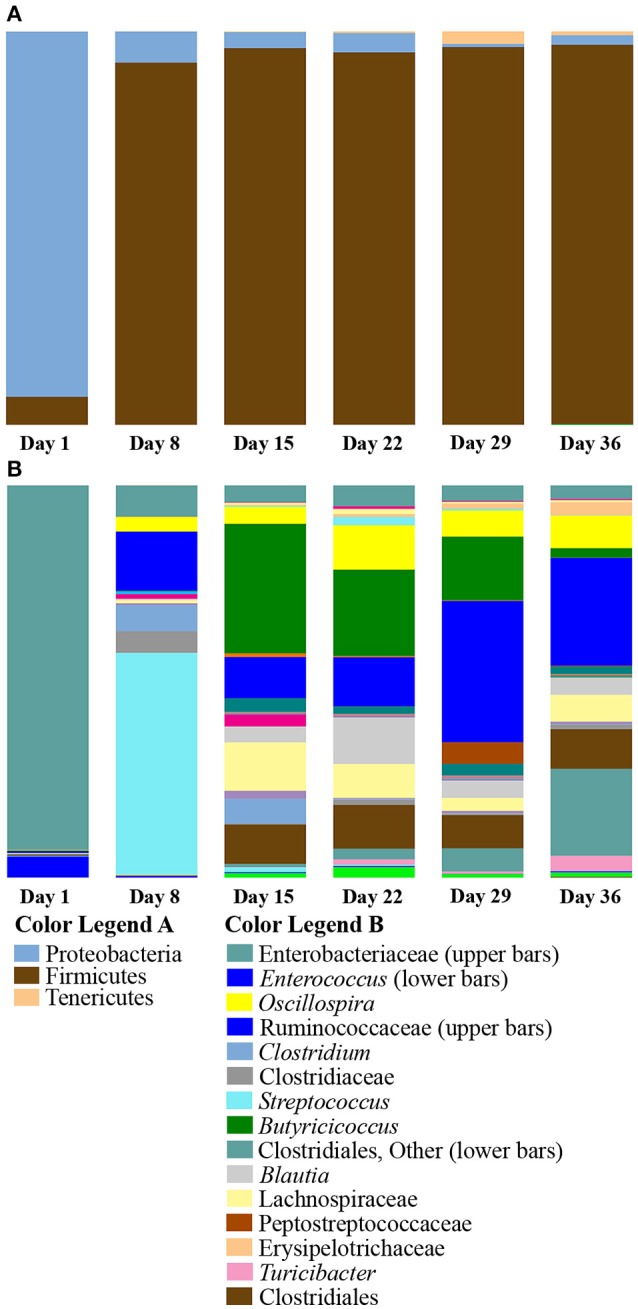
**Summary of bacterial taxa observed by age of duck in Aviary Study 1**. The relative abundances of bacterial 97% operational taxonomic units (OTUs) are shown for duck caecal samples grouped according to age of the duck. **(A)** Depicts phylum level classifications for observed OTUs. OTUs representing smaller proportions of the population are listed in Supplementary Table 5, Taxa Summary Legends. **(B)** Depicts genus level (or higher) classifications for observed OTUs for samples grouped according to the age of the duck. Full color legends for each panel are listed in Supplementary Table 5, Taxa Summary Legends. A major shift from Day 1 to Day 8 occurs at the phylum level. There are clear differences in abundance of particular OTUs from Day 8 through Day 36.

Taxa summaries of the samples grouped by age from Aviary Study 1 revealed a shift in phylum level relative abundances by the Day 8 sampling point (Figure 2A). Day 1 ducks were dominated by the phylum Proteobacteria, ranging from 77 to 99% of the microbial population in an individual. By day 8, the population had shifted to dominance by the phylum Firmicutes, ranging from 81 to 98% of the population in an individual. The dominance of Firmicutes extended through the rest of the grow-out period, making up an average of 96% of the microbial population.

The shift to Firmicutes by day 8 and subsequent maintenance of this shift suggested that a major transitional period in caecal population development occurred prior to day 8. However, it was also apparent from the data that day 8 individuals were different in both the diversity of taxa present and the composition of the population with respect to days 15 through 36. This is borne out by examining the taxonomic composition at ranks below the phylum level, where it is clear that day 8 individuals had distinct relative abundances of different taxa (Figure 2B). In order to further characterize the differences in early and late age ducks, taxonomic groups that are significantly differentially distributed between age groups were determined using DESeq2 (Anders and Huber, 2010) as implemented in phyloseq (McMurdie and Holmes, 2013) (Supplementary Table 3). In pairwise comparisons with late age ducks (days 15, 22, 29, and 36), an average of 43 OTUs were identified as significantly enriched (Benjamini-Hochberg adjusted *p* ≤ 0.05) in day 1 ducks. These OTUs were evenly distributed between the Proteobacteria and Firmicutes in the comparison with day 15, but the distribution was increasingly biased toward members of the Proteobacteria in comparisons with days 22, 29, and 36. The OTUs identified as enriched in the late age ducks as compared to day 1 ducks were almost all members of Firmicutes, with a small number distributed between Actinobacteria and Tenericutes. The largest numbers of enriched OTUs identified in comparisons with day 1 ducks were for days 29 (105 OTUs) and 36 (104 OTUs). In pairwise comparisons of late age ducks and day 8 ducks, an average of 56 OTUs were identified as significantly enriched in day 8 ducks. These OTUs were roughly evenly distributed between Proteobacteria and Firmicutes for all late age comparisons, with a trend toward Firmicutes in comparisons with days 29 and 36. As with the day 1 duck comparisons, the OTUs identified as enriched in the late age ducks as compared to day 8 ducks were almost all Firmicutes, with a small number distributed between Actinobacteria and Tenericutes. The largest number of enriched OTUs in comparisons with day 8 ducks were for days 29 (97 OTUs) and 36 (95 OTUs). A pairwise comparison of day 1 and day 8 ducks identified 36 OTUs and 29 OTUs as significantly enriched for each day, respectively. The day 1 enriched OTUs were evenly distributed between Proteobacteria (17 OTUs) and Firmicutes (19 OTUs), whereas the day 8 enriched OTUs were predominantly from Firmicutes (26 OTUs). For both days, all enriched Proteobacteria OTUs were members of the Enterobacteriaceae; all but one of the enriched Firmicutes OTUs were members of either Bacilli or Clostridiales classes. These results are consistent with the shift to Firmicutes early in the grow-out period and with increased alpha diversity metrics of late aged ducks.

### Aviary study 2: analysis of first 10 days of developmental grow-out period

The data from Aviary Study 1 led us to focus on the first 10 days of the grow-out period in a second group of ducks (Aviary Study 2) in order to more fully characterize the shift from Proteobacteria to Firmicutes. Alpha diversity metrics showed a consistent level of diversity throughout the 10 day period; three of the metrics showed statistically significant differences among age groups (Supplementary Figure 1B, repeated measures ANOVA inset for each metric). The Shannon diversity index exhibited the strongest pattern and showed statistically significant differences between pairwise combinations of early (Days 1, 2, and 3) and late (Days 5, 6, 8, 9, and 10) portions of the study period (*post-hoc t*-tests, Bonferroni corrected *p* < 0.05). The number of observed species ranged from 67 to 93 through the 10 days, consistent with that seen in the early days from Aviary Study 1.

The structures of the duck caecal populations were not as clearly distinguished in PCoA plots, in contrast to the broader age range covered in Aviary Study 1. The trend observed in PC1 (49% of the data explained) appeared to loosely correlate with age (Supplementary Figure 2), and the groupings by age were statistically supported in both weighted (adonis, *DF* = 9, 999 permutations, *F* = 7.7439, *R*^2^ = 0.58, *p* = 0.001) and unweighted UniFrac analyses (adonis, *DF* = 9, 999 permutations, *F* = 1.9546, *R*^2^ = 0.26, *p* = 0.001).

**Figure 3:**
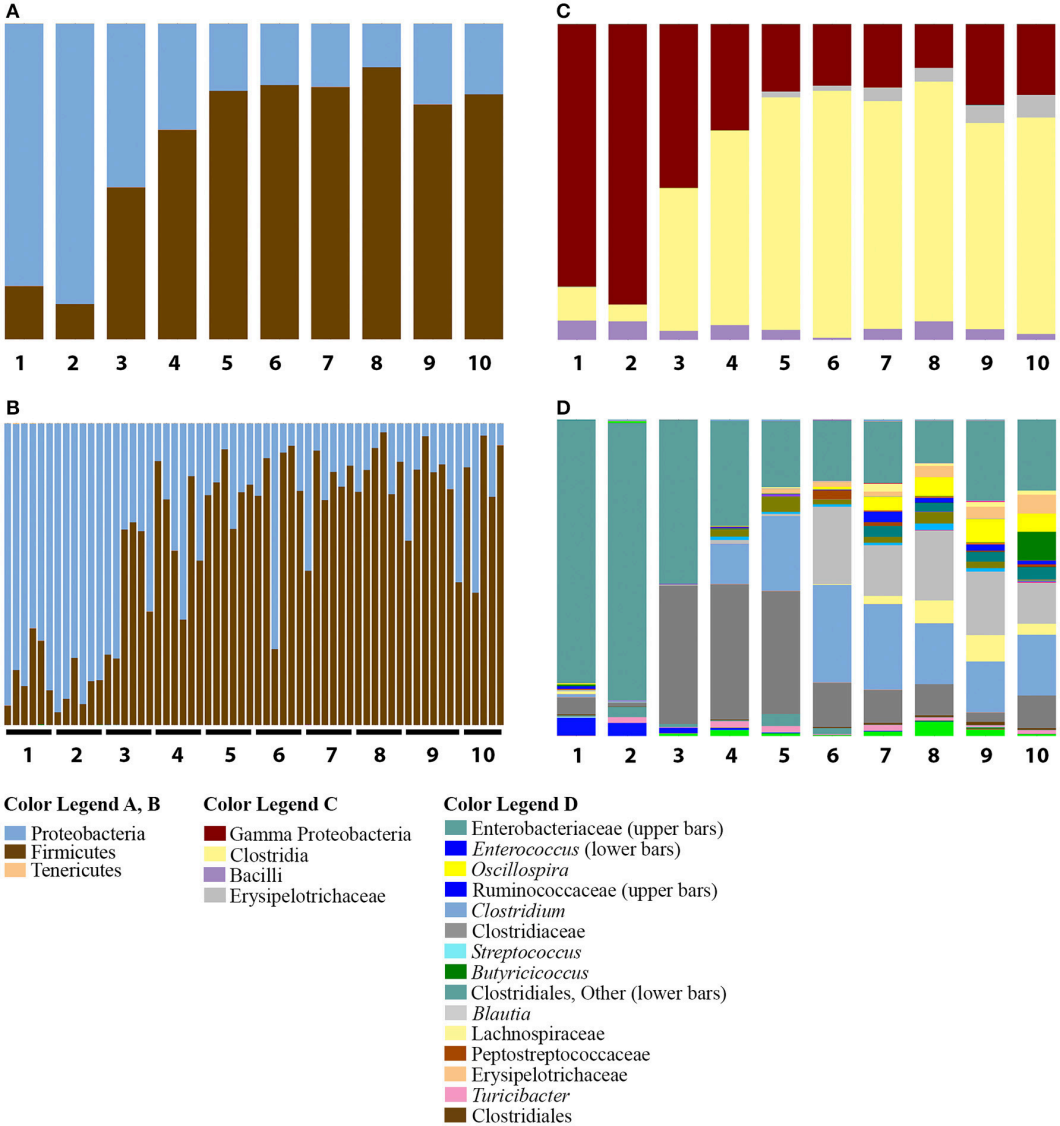
**Summary of bacterial taxa observed by age of duck in Aviary Study 2**. The relative abundances of bacterial 97% operational taxonomic units (OTUs) are shown for duck caecal samples from Aviary Study 2, representing the first 10 days of the grow out period. **(A)** Depicts phylum level classifications for observed OTUs for samples grouped according to age of the duck. Numbers under each bar indicate the age of the ducks in days. **(B)** Depicts phylum level classifications for observed OTUs of individual samples, sorted according to age of the duck. Each horizontal line underneath the bars encompasses the samples derived from ducks of the indicated age in days. **(C)** Depicts class level classifications for observed OTUs for samples grouped according to age of the duck. **(D)** Depicts genus level (or higher) classifications for observed OTUs for samples grouped according to the age of the duck. Full color legends for each panel are listed in Supplementary Table 5, Taxa Summary Legends. The observed transition from Proteobacteria to Firmicutes is seen to occur by Day 3 and occurs in all individuals that make up the age groups **(A,B)**. The distribution of taxa below the phylum level shows that ages 4 and 5 are distinct from ages 6 to 10 **(C,D)**.

Despite weaker trends in PCoA analyses, the summaries of the taxonomic groups observed in age-based categories of ducks recapitulated the major shift from Proteobacteria to Firmicutes by day 8 in the Aviary Study 1. Proteobacterial dominance persisted through the first 2 days of age (Figure 3A; this pattern held for all individuals from days 1 and 2 in the study, Figure 3B). The phylum Firmicutes rose in abundance sharply between days 2 and 3 of age, with an increase from averages of 11 to 48% of the population. Day 3 represented a clear transition—3 out of 6 individuals maintained proteobacterial dominance, and 3 individuals had already shifted to dominance by Firmicutes (Figure 3B). By day 4, the proportion of Firmicutes rose to an average of 66% and stabilized from day 5 through day 10 of the second study at ~78% of the population. Days 2 and 4 were shown to be significantly different in pairwise comparisons of weighted UniFrac distances (adonis, *DF* = 1, 999 permutations, *F* = 18.637, *R*^2^ = 0.65, *p* = 0.005) along with 31 of 45 possible pairwise combinations between Days 1–10 (adonis, *DF* = 1, 999 permutations, *F* = 2.7867–32.345, *R*^2^ = 0.22–0.76, *p* < 0.05).

The transition from Proteobacteria to Firmicutes dominance from days 3 through 10 was characterized by the increase of a small number of major taxonomic groups, including the classes Bacilli, Clostridia, and Erysipelotrichi. The Clostridia comprised the majority of the Firmicutes observed in most individuals from days 3 through 10, ranging from 45 to 78% of the population (Figure 3C). Comparatively smaller populations of Bacilli and Erysipelotrichi existed through this period, though they both rose to over 10% of the population in some individuals.

The dominant taxa within the class Clostridia were members of the families Lachnospiraceae, genus *Blautia* (Clostridiales, Lachnospiraceae); Clostridiaceae, genus *Clostridium* (Clostridiales, Clostridiaceae), and an uncharacterized genus in the family Clostridiaceae; and Ruminococcaceae, genera *Oscillospira* (Clostridiales, Ruminococcaceae) and *Butyricicoccus* (Clostridiales, Ruminococcaceae) (Figure 3D). The genus *Blautia* comprised less than an average of 1% of the population through day 5, but jumped to a peak of 25% of the population on day 6. Through day 10, the genus ranged from 13 to 25% of the population. The genus *Clostridium* was present at less than 1% of the population through day 3, followed by a rise to 13% on day 4, a peak of ~30% on days 6 and 7, and stabilizing at ~17% of the population through day 10. In contrast, the uncharacterized Clostridiaceae genus was present as a large fraction, ~5%, of the population of ducks from day 1, expanded to be ~42% of the population on days 3–5 of the grow-out period, and declined to ~10% of the population on days 6–8 and 10, ranging down to 3% on day 9 (Figure 3D). Thus, in days 3–5, the large increase in Firmicutes is due primarily to a single genus in the Clostridiaceae. The genus *Oscillospira* is less than 1% of the population through day 6, but ranges from 4 to 8% of the population on days 7–10, ending at 5% by day 10. *Oscillospira* represents the only major taxon of the family Ruminococcaceae until day 10, when the genus *Butyricicoccus* blooms to become 9% of the population. Prior to day 10, *Butyricicoccus* is present at less than 0.5% of the population. Each of these groups is often associated with the gut microbiome from a variety of animals (Biddle et al., 2013; Tims et al., 2013; Eren et al., 2014; Geirnaert et al., 2015) and serve as examples of the individual taxon dynamics that occur during the development of ducks.

### Overview of the full 36 day grow-out period

The combination of Aviary Studies 1 and 2 reveal a clear succession of microbial populations through a highly variable early stage to a more stable late stage of development. The fine grained sampling through the first 10 days of development shows that the transition from dominance by Proteobacteria to dominance by Firmicutes occurs by day 4, however there are clear differences in the types of Firmicutes and their relative abundances observed as ducks mature.

Major taxa seen in the first 10 days of the grow-out period also appear as part of the populations seen in late age ducks from Aviary Study 1. Following these major taxa through the rest of the grow-out period shows the marked shift from Proteobacteria to Firmicutes. The proteobacterial population that remains after the shift is dominated by an undefined genus comprising the same two 97% OTUs in both aviary studies. Both OTUs are significantly differentially distributed across ages (Kruskal-Wallis, Bonferroni corrected *p* = 7 × 10^−12^). Within the Firmicutes, the genus *Blautia* peaks at 12% of the population in 22 day old ducks followed by stabilization of the population at ~4% by day 29 (Figure 2B). The percentages of the total population of *Blautia* between days 6 and 22 are well in excess of those seen in other organisms (Eren et al., 2014), though this comparison is to developed, rather than to developing specimens. The family Ruminococcaceae is represented primarily by *Oscillospira* prior to day 10 in Aviary Study 2 ducks (Figure 3D) and is seen to be present in high proportions of the populations in Aviary Study 1 ducks from day 15 to the end of the grow-out period (Figure 2B). In contrast, *Butyricicoccus* appears at day 10 in study 2 ducks, peaks at 33% of the population on day 15 of study 1 ducks, and remains above 15% of the population through day 29 before rapidly decreasing to 5% of the population by day 36 of the grow-out period (Figures 2B, 3D). Another group in the Ruminococcaceae family rapidly rises as a percent of the population in late age ducks, bringing the relative abundance of this family to a range of 19 to 60% from day 15 on (Figure 2B). The two major taxonomic groups within the family Clostridiaceae, *Clostridium* and an undefined genus, actually peak within the first 10 days at up to 19% and 10% of the population followed by a rapid decrease to become ~1% of the population or less by day 36 (Figures 2B, 3D). Thus, even though the phylum level Firmicutes population rises as age increases, the dominant taxa representing Firmicutes early in development are almost fully replaced by other Firmicutes late in development.

Both aviary studies were conducted 6 months apart and overlap with two time points (Day 1 and Day 8 ducks). A DESeq2 analysis of the microbial populations observed in both studies showed that there are 24 OTUs identified as significantly different between Day 1 ducks from both studies and 58 OTUs identified as significantly different between Day 8 ducks (Supplementary Table 3). The larger number of enriched OTUs identified for the day 8 comparison is consistent with the PCoA (Supplementary Figure 3); day 1 ducks from both studies cluster closely in PCoA (Supplementary Figure 3A), whereas day 8 ducks are clearly distinct between the two studies (Supplementary Figure 3). Despite the differences at day 8, many of the taxa observed in the 10 day period of study 2 were observed in later ages of study 1 ducks (cross reference Supplementary Figure 3B with results above).

### Barn study 3: bacteriodetes absent from duck caecal microbiome in aviary setting

Four major bacterial phyla most often associated with animal gut systems are Bacteroidetes, Firmicutes, Proteobacteria, and Actinobacteria (Ley et al., 2008). However, Bacteroidetes was not observed in the aviary data sets. To address whether this absence is due to the environmental setting or to being a unique feature of anseriformes, we analyzed data from a parallel study conducted in an agricultural barn setting (Schenk et al., 2016). The original goal of the parallel barn study (herein referred to as Barn Study 3) was to assess the advantages and disadvantages of watering the ducks with an open water trough or with a pin-metered line system. As such, a limited sampling of duck caecal contents was built into the design, restricted to days 5, 21, and 33 of the grow-out period. The source of the ducks in Aviary Studies 1 and 2 was the same source as the ducks in Barn Study 3. In the comparison of the ducks from the 3 studies, we used ducks from Aviary Study 2, day 5; Aviary Study 1, day 22; and Aviary Study 1, day 36. These ages were then grouped into early, mid and late time points in the grow-out period, respectively, and combined with the corresponding early, mid and late time points from the Barn Study 3 grow-out period for comparative analysis of the microbial populations.

**Figure 4:**
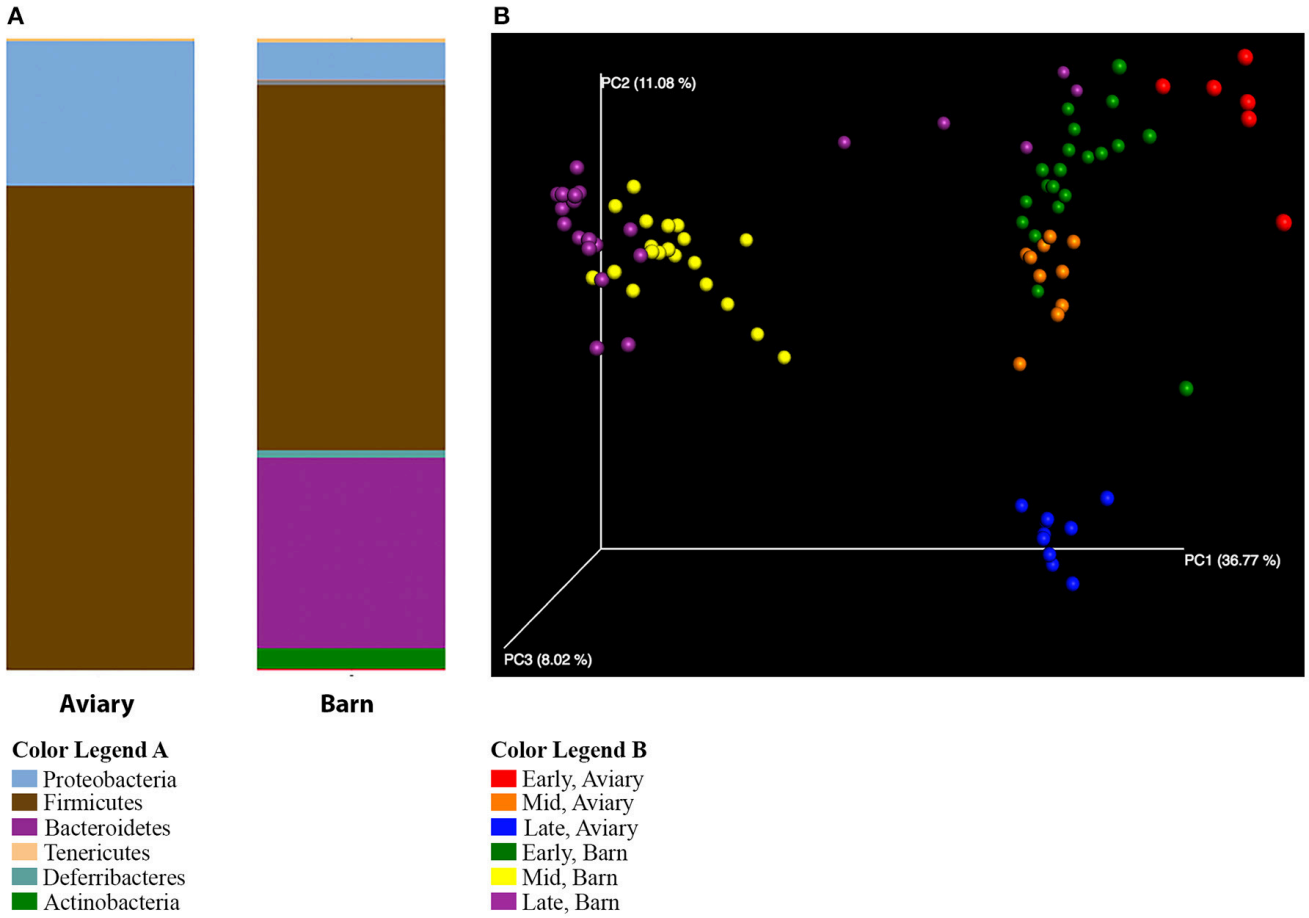
**Comparison of aviary and barn studies. (A)** The relative abundances of bacterial 97% operational taxonomic units (OTUs) are shown for all duck caecal samples from aviary and barn environments at the phylum level of classification. Full color legends for each panel are listed in Supplementary Table 5, Taxa Summary Legends. The ducks in the barn environment have greater phylum level diversity than those in the aviary environment, including a large proportion of Bacteroidetes. **(B)** Principal Coordinate Analysis of Weighted UniFrac Distances for aviary and barn studies. Samples are colored by environment and age group of the duck samples—Red, Early-Aviary; Orange, Mid-Aviary; Blue, Late-Aviary; Green, Early-Barn; Yellow, Mid-Barn; Purple, Late-Barn. Analyses were conducted on data rarefied to 10,000 sequencing reads. Axes represent the percent of variation explained by each principal coordinate. Individual ceacal samples from a given time point during the development cycle and environment group together. There is separation between aviary and barn environments, though early time point barn samples are partially intermixed with aviary samples from the mid time point.

Several observations confirm the expectation that the barn environment is very different from the aviary environment, and are reflected in significant differences in the composition of the caecal microbiome of ducks in both settings. Alpha diversity measures show a marked increase in the diversity of the microbiome in barn-raised ducks compared to aviary-raised ducks. The Shannon entropy for aviary ducks was 3.1 and for barn ducks was 4.6, a statistically significant difference (*t*-test, *t* = −8.12, *p* = 0.001). The number of observed species is also significantly higher in barn-raised ducks (90 vs. 155, respectively; *t*-test, *t* = −12.13, *p* = 0.001). The distributions of taxa observed in aviary vs. barn-raised ducks are consistent with alpha diversity metrics (Figure 4A). As noted before, the major phyla in the aviary ducks are the Proteobacteria (24%) and the Firmicutes (76%). In contrast, the barn-raised ducks contain four major phyla found most often in other animal systems, Firmicutes (57%), Bacteroidetes (30%), Proteobacteria (6%), and Actinobacteria (3%). The differences in the barn and aviary duck caecal microbiomes are clearly seen in PCoA plots as clusters associated with both environmental setting and age (Figure 4B). The factors environment and age and the interaction between the two factors were shown to be statistically significant between the barn and aviary microbiomes (two-way adonis, Environment—*DF* = 1, *F* = 46.961, *R*^2^ = 0.25293, *p* = 0.001; Age Group—*DF* = 2, *F* = 18.294, *R*^2^ = 0.19706, *p* = 0.001; Environment: Age Group—*DF* = 2, *F* = 6.061, *R*^2^ = 0.06528, *p* = 0.001). A DESeq2 analysis of the aviary and barn environments identified 217 OTUs as significantly different between the two environments, 77 significant to the Aviary and 140 significant to the Barn environment (Supplementary Table 4). Ninety-five percent of the OTUs identified in the barn environment are associated with the phyla Firmicutes, Bacteroidetes and Proteobacteria, whereas only Firmicutes and Proteobacteria are represented among enriched OTUs in the aviary environment. All but 8 of these OTUs are associated with the phylum Firmicutes. These data are consistent with increased alpha diversity metrics in the barn environment associated with major bacterial groups.

**Figure 5:**
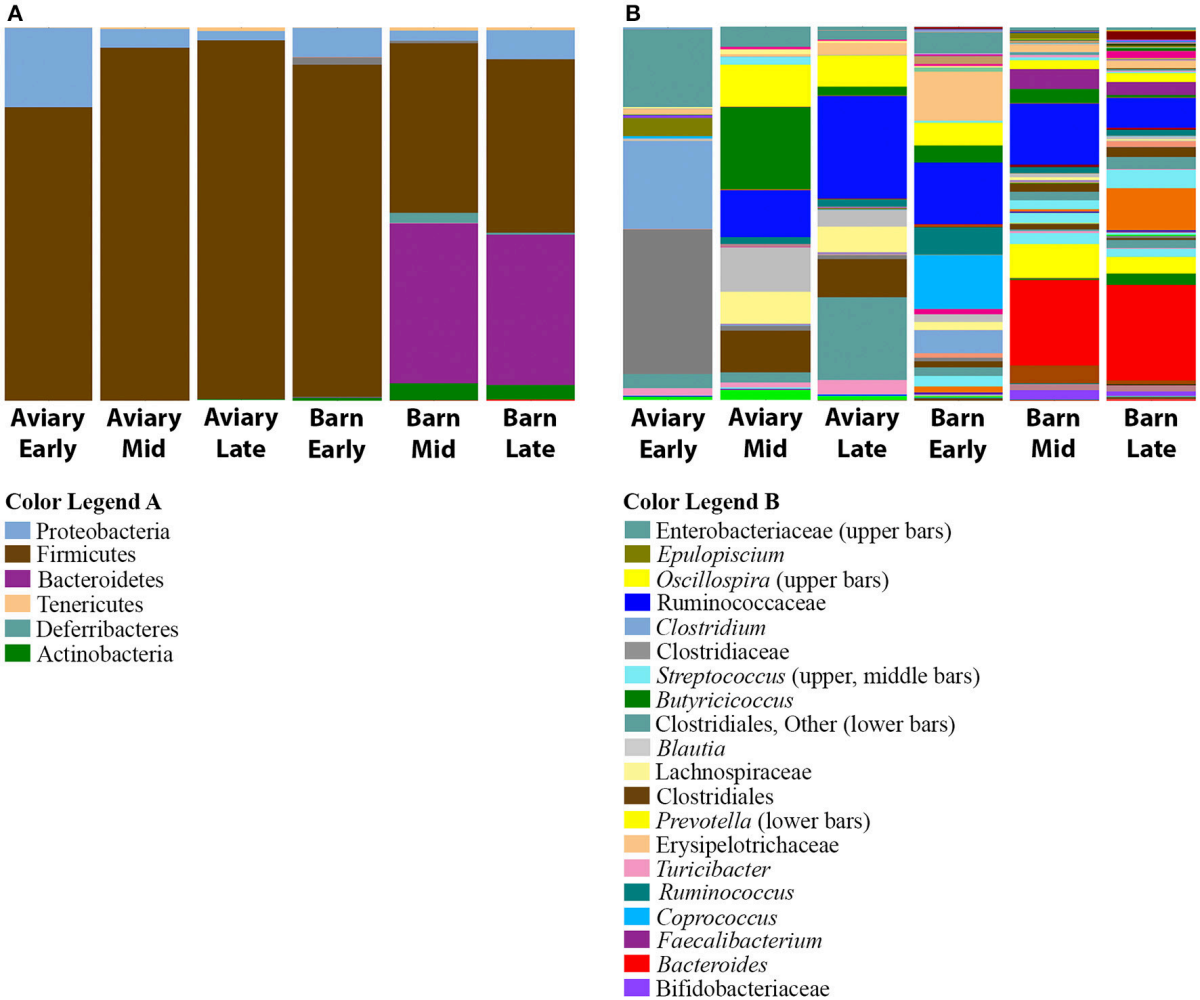
**Comparison of bacterial taxa observed in aviary and barn environments**. The relative abundances of bacterial 97% operational taxonomic units (OTUs) are shown for duck caecal samples from Aviary and Barn studies, representing early, mid and late time points within the grow out period. **(A)** Depicts phylum level classifications for observed OTUs. **(B)** Depicts genus level (or higher) classifications for observed OTUs for samples. Full color legends for each panel are listed in Supplementary Table 5, Taxa Summary Legends. At the phylum level, Bacteroidetes is present in barn-rasied ducks, but it is absent in aviary-raised ducks. There is greater diversity in barn-raised ducks.

Subdividing the aviary and barn raised ducks into early, mid and late age groups reveals that the development progression observed in the aviary setting also takes place in the barn setting (Figure 5). In both early time points (day 5), Firmicutes is the dominant phylum (aviary, 79%; barn, 89%). This is consistent with the shift from Proteobacteria dominance in days 1 and 2 to Firmicutes dominance by day 4 in the aviary setting. The mid and late time points show significant increases in the population of Bacteroidetes in the barn setting, from 0.2% on day 5 to ~40% on days 21 and 33. Within the aviary setting, the numbers of significantly enriched OTUs in aviary-early and aviary-mid/aviary-late pairwise comparisons using DESeq2 are 126 and 116, respectively. For the same pairwise comparisons within the barn setting, the numbers of enriched OTUs are 223 and 219. Pairwise comparisons between the two environments matched by age grouping show 121 OTUs (aviary/barn early), 200 OTUs (aviary/barn mid), and 200 OTUs (aviary/barn late) as significantly enriched, with 77, 64, and 59% of OTUs being enriched in the barn environment in each comparison, respectively (Supplementary Table 4). In all age based comparisons between the environments, Bacteroidetes OTUs are identified as significantly enriched (early, 3; mid, 27; late, 29). The Firmicutes represents the largest fraction of significantly enriched OTUs in the barn setting at all age comparisons (early, 89%; mid, 59%; late, 49%), highlighting the variability at the OTU level seen between the two environments for major taxonomic groups. Despite the taxon level diversity between the environments, similar shifts in high level taxonomic ranks are observed in both environments. All pairwise comparisons within and between environments grouped by age are provided in Supplementary Table 4.

### Evaluation of common anseriforme bacterial pathogens

The five most common bacterial pathogens associated with ducks in the production environment are *Riemerella anatipestifer, Escherichia coli, Salmonella, Streptococcus*, and *Enterococcus*. Of these, the focus of most monitoring efforts are *R. anatipestifer, E. coli*, and *Streptococcus* (personal communication with Dr. Dan Shafer, Vice-President Live Production, MLF, Inc.). The OTU assignments for ducks raised in the aviary and barn settings were queried for these organisms. None of the assigned OTUs were identified as *R. anatipesifter, E. coli*, or *Salmonella*, however, assignments to the genus level for *Escherichia* are problematic with the greenegenes database used in this study and in many current microbiome studies (Nelson et al., 2014). Assignments to Enterobacteriaceae were present, which is the group harboring *E. coli* and *Salmonella*. Both *Streptococcus* and *Enterococcus* were identified as part of the microbiome of the duck caecum in both aviary and barn settings, and *Streptococcus* was found to be a dominant part of the population in some ducks. Taxa summaries of the ducks grouped by study and day show that the microbiomes of Aviary Study 1 ducks at Day 8 were dominated by a population of the genus *Streptococcus* (Firmicutes, Bacilli) making up 57% of the population (Supplementary Figure 3B). The *Streptococcus* population was found in Day 8 ducks from Aviary Study 2, but at a very low percentage of the total population (9.2 × 10^−5^%). The *Streptococcus* genus that dominates Day 8 ducks from Aviary Study 1 is comprised of seven 97% OTUs, but a single OTU represents 99.8% of the population. This OTU is also present in ducks from Aviary Study 2, albeit at a very small proportion of the total population in study 2 ducks, and it is significantly differentially distributed between the two studies (Mann-Whitney U, Bonferroni corrected *p* = 0.002) and across ages through the entire grow-out period (Kruskal-Wallis, Bonferroni corrected *p* = 0.0004). The same OTU is also identified in ducks from Barn Study 3 at low percentages of the population. Despite the presence of these taxa, all ducks in the three, independent studies were healthy and reached market weights by Day 36 of the grow-out period.

## Discussion

Commercial farming of Pekin ducks is a multibillion dollar industry worldwide, with the production of over 24 million ducks per year in the United States, alone. This study provides the first assessment of the microbial populations present in the caecum of a commercial strain of Pekin duck throughout a 36-day grow-out period that is typical of industry practices. In particular, the developmental progression is characterized by a major transition from Proteobacteria in the first 2 days of age to dominance by Firmicutes by 5 days of age. This transition is observed in both aviary and industry barn settings, but there are stark differences in the diversity and composition of the microbial populations in the two settings. As discussed below, there are clear differences between anseriformes and galliformes, indicating that it is not advisable to extrapolate results of studies affecting the microbiomes from one bird order to another. These results highlight the necessity of careful experimental design as industries consider improvements to management practices based on nutrition, feed, prebiotic, probiotic, and antibiotic usage.

The caecae were utilized in this study because of their role in digestion and the overall health of birds. With internal villi, a sphincter and a blind end, nutrient-rich liquid contents of the digestive tract are concentrated in the caecae via reverse-peristalsis from the small intestine and colon (for reviews of avian gastric motility, see Duke, 1982; Clench and Mathias, 1995). The internal villi allow for nutrient uptake while the blind end allows for increased content retention time and continuous reverse peristalsis to retain contents within the digestive tract (Shibata and Sogou, 1982). With decreased oxygen levels, this area becomes ideal for increased bacterial loads, aiding in digestion and uptake of crucial nutrients. The development of the digestive system of the Pekin duck occurs rapidly posthatch. In particular, it has been shown that the ileal and jejunal mucosa undergo large morphological changes during the first 7 days posthatch, including increases in villus height and crypt depth (Applegate et al., 1999, 2005). These changes coincide with drastic changes in the microbiome of the caecum from dominance by Proteobacteria to Firmicutes observed in this study. In comparison to galliforms, other areas of the digestive tract have been found to have less bacterial diversity possibly due to the constant motion of contents through the tract (Gong et al., 2007; Stanley et al., 2014). Even so, the filling of contents in the caecum from the small intestine suggest that these developmental changes could have significant impact on the microbiome and microenvironments found within the caecum.

Surprisingly, the phylum Bacteroidetes was not observed in any of the ducks from aviary studies 1 and 2 at significant levels. This group, to our knowledge, is always associated with gut microbiomes in other animals and has been linked to health states of individuals (Ley et al., 2008; Cho and Blaser, 2012; Tims et al., 2013). For example, shifts in Firmicutes/Bacteroidetes ratios have been observed in lean and obese model systems (Gilbert et al., 2016), as well as in broiler chickens and Pekin ducks based on fecal swabs and detection via qPCR (Angelakis and Raoult, 2010). Further, Bacteroidetes was found to be a dominant phylum present in the caecal contents of 12–14 week old Pekin ducks (Vasaï et al., 2014). While both aviary studies were conducted in the same aviary setting, they were separated by 6 months in time. Bacteroidetes was identified in water samples from the aviary setting (data not shown), suggesting that the ducks were ingesting a potential source of this group of bacteria. However, this group of organisms did not establish a detectable population in the duck caecal cavity. Given that these are the first comprehensive data sets for the developing Pekin duck caecal microbiome, it was possible that the composition is very different from all other available data from animal gut environments. In a parallel study by our group (Schenk et al., 2016), we investigated the caecal contents of ducks raised in a commercial production environment that used well water and an open barn design, allowing us to compare the aviary and barn data sets to determine if the pattern of absence of Bacteroidetes as a major component of the microbiome of Pekin ducks was consistent across environments. These results suggest that the absence of Bacteroidetes in ducks raised in the aviary setting is dependent on local environmental factors. The aviary is kept clean throughout the time that ducks are present and sterilized bedding is used, whereas in a barn setting with thousands of ducks and open air flow, the ducks will be exposed to many more sources of environmental microbes. In all cases, caecal contents came from healthy, well developing individuals, which raises questions about how different microbial populations in the caecum affect agriculturally important phenotypes.

The most common pathogen in anseriformes is *Riemerella anatipestifer (RA)* (Wobeser, 1997), which occurs globally in wild and commercially raised ducks (Brogden, 1989). RA went unexplored for quite some time, and has only recently been taxonomically classified and further characterized (Ryll et al., 2008). Its pathogenesis remains unknown, though we hypothesized that it might be part of the endogenous gut microbiome and that it may become opportunistic under the right environmental conditions. However, RA was not observed in any caecal contents in this study. The respiratory tract and the epidermis represent two other areas of the Pekin duck that could harbor a subpopulation of RA and are of interest in further studies. Sequences of rRNA genes of non-serotypable RA-like strains isolated from the pharyngeal flora of healthy Pekin ducks were found to be 99% identical to those of RA (Ryll et al., 2008). Other genera that include pathogens, such as *Streptococcus* and *Enterococcus* appear to be normal constituents of the duck caecal microbiome, comprising up to 50% of the population during development in some individuals and in later aged ducks (Vasaï et al., 2014). The ducks in this study harboring *Streptococcus* were healthy, and this highlights the distinction between presence of a potential pathogen and actual instance of disease (Casadevall and Pirofski, 2014).

**Figure 6:**
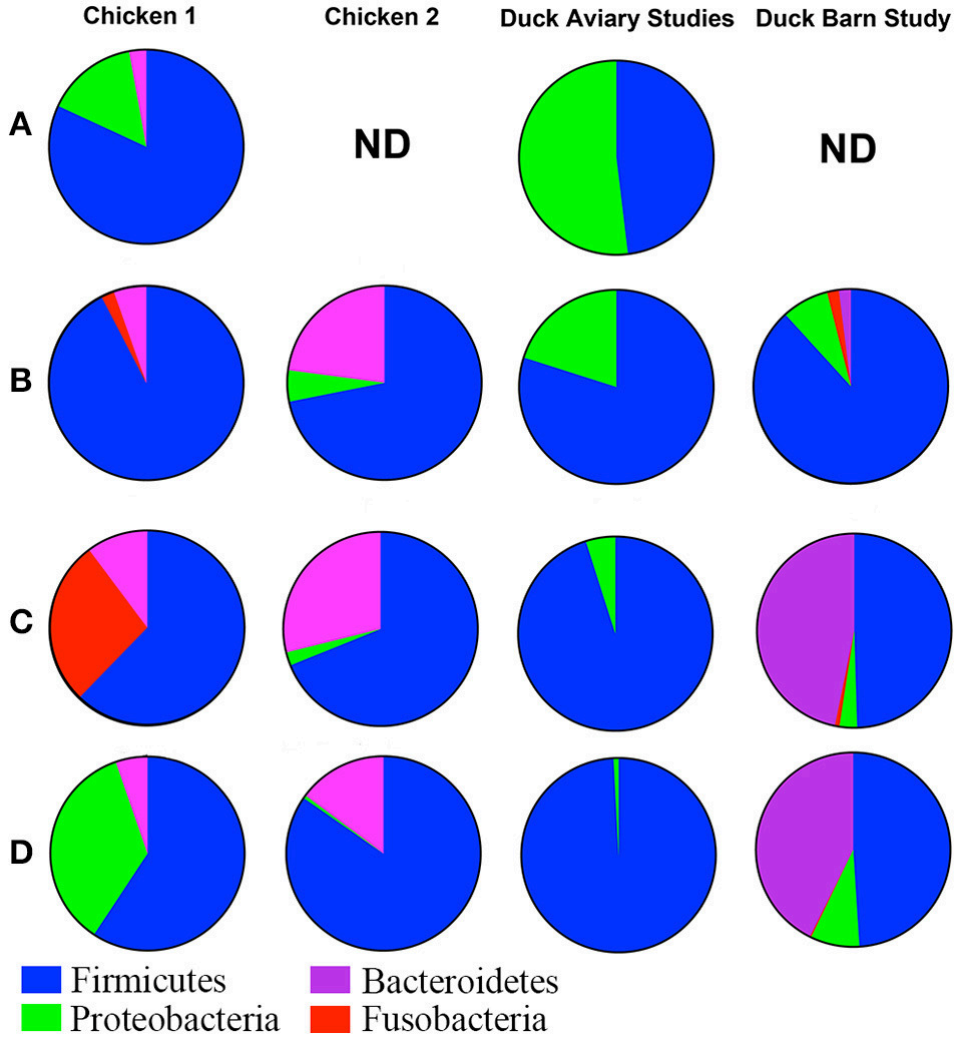
**Qualitative comparison of galliforme and anseriforme caeca contents at the phylum level**. Row **(A)** Mid-Week 1. Chicken data from Lu et al. (2003). ND, no data available. Row **(B)** End of week 1. Chicken 1 data from Lu et al. (2003) and Chicken 2 data from Asrore et al. (2015). Row **(C)** End of Week 3. Chicken 1 data from Lu et al. (2003) and Chicken 2 data from Asrore et al. (2015). Row **(D)** End of week 4. Chicken 1 data from Lu et al. (2003) and Chicken 2 data from Choi et al. (2014). Overall, chickens show predominately *Firmicutes* in the cecae early in life whereas ducks show near equal proportions of both *Firmicutes* and *Proteobacteria*. Though compositionally chicken and duck are similar, the levels of Firmicutes decline by the end of the growth period in chickens (left 2 columns), while they maintain levels in ducks (two right columns). As chickens age (rows), *Fusobacteria* increases and *Bacteroides* varies. Proteobacteria is much higher in ducks throughout the grow-out. Ducks raised in the aviary at Hope College showed no Bacteroidetes whereas ducks raised in the barn setting showed high levels of Bacteroidetes compared even to the chicken data. Turkey data could not be included due to collection only at 18 weeks.

While there are many caveats associated with comparison of microbial population data across different studies (Stanley et al., 2013; Gilbert et al., 2016), we present a high level comparison of galliforme and anseriforme caecal contents to identify major differences that may be inherent to the two bird types. Data taken from publications that describe the microbiome of chickens (Lu et al., 2003; Choi et al., 2014; Asrore et al., 2015) and turkeys (Scupham et al., 2008) were used for the qualitative assessment of caecal contents among different commercial birds and are illustrated in Figure 6. The primary difference at a phylum level comparison is the persistence of Proteobacteria throughout the maturation period of the duck, whereas this phylum is a very low percentage of the population or undetectable in broilers after the earliest sampled time point of 3 days posthatch. Broiler caeca microbial populations are dominated by Firmicutes from an early age and persist through >40 days posthatch, comprising well over half of the population (Lu et al., 2003; Choi et al., 2014; Asrore et al., 2015). Turkey caeca microbial populations at 18 weeks of age, are comprised of 52% Bacteroidetes, 33% Firmicutes, 5% Proteobacteria, 4% Deferribacteres, and 6% unclassified bacteria (Scupham et al., 2008). Each of these taxa are represented in the barn-raised ducks at the most mature time point tested at 40% (Bacteroidetes), 46% (Firmicutes), 8% (Proteobacteria), and 0.6% (Defferibacteres). Additional phyla represented in the most mature age group for ducks include Tenericutes (0.7%) and Actinobacteria (4%) (Figure 5A), neither of which were reported as present at more than a fraction of a percent in broilers and turkey. The differences within the galliforme genus and between galliformes and anseriformes may be the result of host genetics, environmental conditions, feed type, or immunity against species-specific pathogens. Various other factors may be at play causing differences between data sets including farming practices, age, breed, and experimental design of studies. However, it has been shown that even within a single study of chickens that the microbiomes of the different study groups can vary significantly (Stanley et al., 2013). Turkeys included in this comparison were sacrificed at 18 weeks of age, which is much older than a 1- to 4-week old chick or duckling. This age component, in itself, may have elicited considerable observed differences in caecal contents among the avian species. However, it is apparent that the caecal contents of anseriformes and galliformes are different, consistent with known differences in physiology and development (Applegate et al., 2005). Thus, it is possible that differences in environment or in feed composition could have considerably different effects in galliforms compared to anseriforms.

Prebiotic and probiotic supplementation has been popular in commercial farming to increase resistance to disease, increase growth rates, and improve overall poultry health (Lee et al., 1999; Roto et al., 2015). Ideal cocktails of probiotics are constantly being evaluated, singling out specific species that will prove to have the most positive effects. Understanding which bacteria are beneficial and how they promote health may improve methods to increase growth yields and decrease disease rates. Lactic acid bacteria such as *Lactobacillus acidophilus* have been shown to increase growth rates while decreasing *E. coli* roduction and are commonly used in commercial poultry feed (Watkins et al., 1982). *Lactobacillus salivarius* and *Lactobacillus agilis* have also been shown to increase growth yields in broiler chickens (Lan et al., 2003). Though widely used, probiotic effects and consequences *in vivo* are not extensively understood, especially in anseriformes. Members of Clostridia cluster IV, a group known to be associated with butyrate production and to be important for gut homeostasis in mammals (Lopetuso et al., 2013), were identified as significant members of the microbiome of ducks in this study. It has been shown that changes in the distribution of these group members, in particular *Oscillospira*, at the order level and below are associated with differences in body mass index (BMI) between pairs of monozygotic twins (Tims et al., 2013). *Butyricicoccus* is associated with butyrate production and is a development target for probiotics aimed at mitigating symptoms of irritable bowel syndrome (Geirnaert et al., 2015). These observations raise interesting possibilities for studying probiotic supplementation of duck feeding practices and for study of differential weight gain in ducks. Introduction of probiotics may induce changes in endogenous microbiome populations, possibly creating new outlets for disease expression and immune system alterations. Creating a reference point in healthy ducks not receiving dietary supplements for comparison to microbial caecal contents from treated ducks will allow for changes to be tracked and analyzed as health changes occur. However, the observation that major phylum level changes can occur with changes in the setting for a study (here, the absence of Bacteroidetes in Aviary Studies 1 and 2 compared to the Barn Study 3) dictates careful design of microbiome based studies to include internal controls rather than reliance on comparison between different studies. This is consistent with recently recommended best practices for microbiome experimental design (Goodrich et al., 2014; Westcott and Schloss, 2015).

In summary, microbial population succession correlated strongly with duck age, exhibiting a clear transition in dominant taxa as ducks matured. Caecal contents of ducklings showed high levels of Proteobacteria that decreased with age, but was maintained at a higher proportion of the population than seen in chicken or turkey. The taxonomic transition led to a dominance of Firmicutes for the remainder of the ducks' life span in an aviary setting. In contrast, the transition led to two major phyla, Firmicutes, and Bacteroidetes, in ducks raised in a barn setting. This taxonomic milieu proved to be much different than both broiler chicken and turkey gut microbiomes described previously (Lu et al., 2003; Scupham et al., 2008; Choi et al., 2014), whereas later time points in development assessed in this study are consistent with caecal microbiomes of 12–14 week old Pekin ducks (Vasaï et al., 2014). *R. anatipestifer* was not found in the samples collected in either aviary or farm settings; other genera that contain common pathogens of anseriformes were identified. Characterization of microbiomes reflective of overall healthy ducks will be used for further assessments of commercial production practices. In particular, these data will allow for investigation of the origin and development of pathogens in commercial flocks, evaluation, and development of prebiotics and probiotics, other practices that potentially improve growth yields, and maintenance of food safety.

## Author contributions

AB, SF, and GS designed the study. AP, SF, and GF acquired and processed samples in aviary and barn environments. AB and AP performed data analyses and drafted the manuscript. All authors participated in data interpretation and manuscript editing.

## Funding

The authors thank Maple Leaf Farms, Inc. for their support for this research project. This work was funded in part by National Science Foundation DBI Award 1229585 to AB and by the National Science Foundation REU award 0754293 to GF (Co-PI).

### Conflict of interest statement

The authors declare that the research was conducted in the absence of any commercial or financial relationships that could be construed as a potential conflict of interest.

## References

[B1] AGMRC (2012). Ducks and Geese, Ag Marketing Resource Center. Available online at: http://www.agmrc.org/commodities-products/livestock/poultry/ducks-and-geese/

[B2] Anders S. Huber W. (2010). Differential expression analysis for sequence count data. Genome Biol. 11:R106. 10.1186/gb-2010-11-10-r106 20979621 PMC3218662

[B3] Angelakis E. Raoult D. (2010). The increase of Lactobacillus species in the gut flora of newborn broiler chicks and ducks is associated with weight gain. PLoS ONE 5:e10463. 10.1371/journal.pone.0010463 20454557 PMC2864268

[B4] Applegate T. J. Karcher D. M. Lilburn M. S. (2005). Comparative development of the small intestine in the turkey poult and Pekin duckling. Poult. Sci. 84, 426–431. 10.1093/ps/84.3.426 15782911

[B5] Applegate T. J. Ladwig E. Weissert L. Lilburn M. S. (1999). Effect of hen age on intestinal development and glucose tolerance of the Pekin duckling. Poult. Sci. 78, 1485–1492. 10560818 10.1093/ps/78.11.1485

[B6] Asrore S. M. M. Sieo C. C. Chong C. W. Gan H. M. Ho Y. W. (2015). Deciphering chicken gut microbial dynamics based on high-throughput 16S rRNA metagenomics analyses. Gut Pathog. 7, 1–12. 10.1186/s13099-015-0051-7 25806087 PMC4372169

[B7] Biddle A. Stewart L. Blanchard J. Leschine S. (2013). Untangling the genetic basis of fibrolytic specialization by lachnospiraceae and ruminococcaceae in diverse gut communities. Diversity 5, 627–640. 10.3390/d5030627

[B8] Bokulich N. A. Subramanian S. Faith J. J. Gevers D. Gordon J. I. Knight R.. (2013). Quality-filtering vastly improves diversity estimates from Illumina amplicon sequencing. Nat. Methods 10, 57–59. 10.1038/nmeth.2276 23202435 PMC3531572

[B9] Brogden K. A. (1989). Pasteurella anatipestifer infection, in Pasteurella and Pasteurellosis, eds. Adlam C. Rutter J. (London: Academic Press, Inc.), 115–129.

[B10] Caporaso J. G. Bittinger K. Bushman F. D. Desantis T. Z. Andersen G. L. Knight R. (2010a). PyNAST: a flexible tool for aligning sequences to a template alignment. Bioinformatics 26, 266–267. 10.1093/bioinformatics/btp636 19914921 PMC2804299

[B11] Caporaso J. G. Kuczynski J. Stombaugh J. Bittinger K. Bushman F. D. Costello E. K.. (2010b). QIIME allows analysis of high-throughput community sequencing data. Nat. Methods 7, 335–336. 10.1038/nmeth.f.303 20383131 PMC3156573

[B12] Caporaso J. G. Lauber C. L. Walters W. A. Berg-Lyons D. Huntley J. Fierer N.. (2012). Ultra-high-throughput microbial community analysis on the Illumina HiSeq and MiSeq platforms. ISME J. 6, 1–4. 10.1038/ismej.2012.8 22402401 PMC3400413

[B13] Casadevall A. Pirofski L. A. (2014). Ditch the term pathogen. Nature 516, 165–166. 10.1038/516165a 25503219

[B14] Cho I. Blaser M. J. (2012). The human microbiome: at the interface of health and disease. Nat. Rev. Genet. 13, 260–270. 10.1038/nrg3182 22411464 PMC3418802

[B15] Choi J. H. Kim G. B. Cha C. J. (2014). Spatial heterogeneity and stability of bacterial community in the gastrointestinal tracts of broiler chickens. Poult. Sci. 93, 1942–1950. 10.3382/ps.2014-03974 24931967

[B16] Clench M. H. Mathias J. R (1995). The avian cecum: a review. Wilson Bull. 107, 93–121. Available online at: http://www.jstor.org/stable/4163516

[B17] D'Argenio V. Salvatore F. (2015). The role of the gut microbiome in the healthy adult status. Clin. Chim. Acta 451, 97–102. 10.1016/j.cca.2015.01.003 25584460

[B18] DeSantis T. Z. Hugenholtz P. Larsen N. Rojas M. Brodie E. L. Keller K.. (2006). Greengenes, a chimera-checked 16S rRNA gene database and workbench compatible with ARB. Appl. Environ. Microbiol. 72, 5069–5072. 10.1128/AEM.03006-05 16820507 PMC1489311

[B19] Dixon P. (2003). VEGAN, a package of R functions for community ecology. J. Veg. Sci. 14, 927–930. 10.1658/1100-9233(2003)014[0927:VAPORF

[B20] Duke G. E. (1982). Gastrointestinal motility and its regulation. Poult. Sci. 61, 1245–1256. 7134105 10.3382/ps.0611245

[B21] Edgar R. C. (2010). Search and clustering orders of magnitude faster than BLAST. Bioinformatics 26, 2460–2461. 10.1093/bioinformatics/btq461 20709691

[B22] Eren A. M. Sogin M. L. Morrison H. G. Vineis J. H. Fisher J. C. Newton R. J.. (2014). A single genus in the gut microbiome reflects host preference and specificity. ISME J. 9, 1–11. 10.1038/ismej.2014.97 24936765 PMC4274434

[B23] Flint H. J. Scott K. P. Duncan S. H. Louis P. Forano E. (2012). Microbial degradation of complex carbohydrates in the gut. Gut Microbes 3, 289–306. 10.4161/gmic.19897 22572875 PMC3463488

[B24] Frank D. N. St Amand A. L. Feldman R. A. Boedeker E. C. Harpaz N. Pace N. R. (2007). Molecular-phylogenetic characterization of microbial community imbalances in human inflammatory bowel diseases. Proc. Natl. Acad. Sci. U.S.A. 104, 13780–13785. 10.1073/pnas.0706625104 17699621 PMC1959459

[B25] Funkhouser L. J. Bordenstein S. R. (2013). Mom knows best: the universality of maternal microbial transmission. PLoS Biol. 11:e1001631. 10.1371/journal.pbio.1001631 23976878 PMC3747981

[B26] Gabriel I. Lessire M. Mallet S. Guillot J. F. (2006). Microflora of the digestive tract: critical factors and consequences for poultry. Worlds Poult. Sci. J. 62, 499–511. 10.1079/WPS2006111

[B27] Geirnaert A. Wang J. Tinck M. Steyaert A. Van den Abbeele P. Eeckhaut V.. (2015). Interindividual differences in response to treatment with butyrate-producing Butyricicoccus pullicaecorum 25-3T studied in an *in vitro* gut model. FEMS Microbiol. Ecol. 91:fiv054. 10.1093/femsec/fiv054 25999470

[B28] Gevers D. Kugathasan S. Denson L. A. Vázquez-Baeza Y. Van Treuren W. Ren B.. (2014). The treatment-naive microbiome in new-onset Crohn's disease. Cell Host Microbe 15, 382–392. 10.1016/j.chom.2014.02.005 24629344 PMC4059512

[B29] Gilbert J. A. Quinn R. A. Debelius J. Xu Z. Z. Morton J. Garg N.. (2016). Microbiome-wide association studies link dynamic microbial consortia to disease. Nature 535, 94–103. 10.1038/nature18850 27383984

[B30] Gong J. Si W. Forster R. J. Huang R. Yu H. Yin Y.. (2007). 16S rRNA gene-based analysis of mucosa-associated bacterial community and phylogeny in the chicken gastrointestinal tracts: from crops to ceca. FEMS Microbiol. Ecol. 59, 147–157. 10.1111/j.1574-6941.2006.00193.x 17233749

[B31] Goodrich J. K. Di Rienzi S. C. Poole A. C. Koren O. Walters W. A. Caporaso J. G.. (2014). Conducting a microbiome study. Cell 158, 250–262. 10.1016/j.cell.2014.06.037 25036628 PMC5074386

[B32] Haas B. J. Gevers D. Earl A. M. Feldgarden M. Ward D. V. Giannoukos G.. (2011). Chimeric 16S rRNA sequence formation and detection in Sanger and 454-pyrosequenced PCR amplicons. Genome Res. 21, 494–504. 10.1101/gr.112730.110 21212162 PMC3044863

[B33] Lan P. T. N. Binh L. T. Benno Y. (2003). Impact of two probiotic Lactobacillus strains feeding on fecal lactobacilli and weight gains in chicken. J. Gen. Appl. Microbiol. 49, 29–36. 10.2323/jgam.49.29 12682864

[B34] Lee Y. K. Nomoto K. Salminen S. Gorbach S. (1999). Handbook of Probiotics. New York, NY: Wiley Interscience.

[B35] Lewis J. D. Chen E. Z. Baldassano R. N. Otley A. R. Griffiths A. M. Lee D.. (2015). Inflammation, antibiotics, and diet as environmental stressors of the gut microbiome in pediatric crohn's disease. Cell Host Microbe 18, 489–500. 10.1016/j.chom.2015.09.008 26468751 PMC4633303

[B36] Ley R. E. Hamady M. Lozupone C. Turnbaugh P. J. Ramey R. R. Bircher J. S.. (2008). Evolution of mammals and their gut microbes. Science 320, 1647–1651. 10.1126/science.1155725 18497261 PMC2649005

[B37] Lopetuso L. R. Scaldaferri F. Petito V. Gasbarrini A. (2013). Commensal clostridia: leading players in the maintenance of gut homeostasis. Gut Pathog. 5:23. 10.1186/1757-4749-5-23 23941657 PMC3751348

[B38] Lozupone C. Knight R. (2005). UniFrac: a new phylogenetic method for comparing microbial communities. Appl. Environ. Microbiol. 71, 8228–8235. 10.1128/AEM.71.12.8228-8235.2005 16332807 PMC1317376

[B39] Lozupone C. Lladser M. E. Knights D. Stombaugh J. Knight R. (2011). UniFrac: an effective distance metric for microbial community comparison. ISME J. 5, 169–172. 10.1038/ismej.2010.133 20827291 PMC3105689

[B40] Lu J. Idris U. Harmon B. Hofacre C. Maurer J. J. Lee M. D. (2003). Diversity and succession of the intestinal bacterial community of the maturing broiler chicken. Appl. Environ. Microbiol. 69, 6816–6824. 10.1128/AEM.69.11.6816-6824.2003 14602645 PMC262306

[B41] McDonald D. Price M. N. Goodrich J. Nawrocki E. P. DeSantis T. Z. Probst A.. (2012). An improved Greengenes taxonomy with explicit ranks for ecological and evolutionary analyses of bacteria and archaea. ISME J. 6, 610–618. 10.1038/ismej.2011.139 22134646 PMC3280142

[B42] McGlone J. Swanson J. Ford S. Mitloehner F. Grandin T. Ruegg P. (2010). Guide for the Care and Use of Agricultural Animals in Research and Teaching, 3rd Edn. Champaign, IL: Federation of Animal Science Societies Available online at: http://www.fass.org

[B43] McMurdie P. J. Holmes S. (2013). Phyloseq: an R Package for reproducible interactive analysis and graphics of microbiome census data. PLoS ONE 8:e61217. 10.1371/journal.pone.0061217 23630581 PMC3632530

[B44] Navas-Molina J. A. Peralta-Sánchez J. M. González A. McMurdie P. J. Vázquez-Baeza Y. Xu Z. (2013). Advancing our understanding of the human microbiome using QIIME, in Methods in Enzymology, ed. Delong E. F. (New York, NY: Elsevier Inc.), 371–444. 10.1016/B978-0-12-407863-5.00019-8 PMC4517945 24060131

[B45] Nelson M. C. Morrison H. G. Benjamino J. Grim S. L. Graf J. (2014). Analysis, optimization and verification of Illumina-generated 16S rRNA gene amplicon surveys. PLoS ONE 9:e94249. 10.1371/journal.pone.0094249 24722003 PMC3983156

[B46] Neo E. La T. Phillips N. D. Alikani M. Y. Hampson D. J. (2013). The pathogenic intestinal spirochaete *Brachyspira pilosicoli* forms a diverse recombinant species demonstrating some local clustering of related strains and potential for zoonotic spread. Gut Pathog. 5:24. 10.1186/1757-4749-5-24 23957888 PMC3751851

[B47] Nicholson J. K. Holmes E. Wilson I. D. (2005). Gut microorganisms, mammalian metabolism and personalized health care. Nat. Rev. Micro. 3, 431–438. 10.1038/nrmicro1152 15821725

[B48] Van Opstal E. J. V. Bordenstein S. R. (2015). Rethinking heritability of the microbiome. Science. 349, 1172–1173. 10.1126/science.aab3958 26359393

[B49] Pérez de Rozas A. M. (2004). A comparative study of intestinal microbial diversity from birds, pigs and rabbits by Restriction Fragment Length Polymorphism analysis, in Reproduction Nutrition Development, eds Thiéry J.-C. Guesnet P. Guillomot M. (London: EDP Sciences), S4.

[B50] Price M. N. Dehal P. S. Arkin A. P. (2010). FastTree 2–Approximately maximum-likelihood trees for large alignments. PLoS ONE 5:e9490. 10.1371/journal.pone.0009490 20224823 PMC2835736

[B51] Reid G. Younes J. A. van der Mei H. C. Gloor G. B. Knight R. Busscher H. J. (2011). Microbiota restoration: natural and supplemented recovery of human microbial communities. Nat. Rev. Microbiol. 9, 27–38. 10.1038/nrmicro2473 21113182

[B52] Roto S. M. Rubinelli P. M. Ricke S. C. (2015). An introduction to the avian gut microbiota and the effects of yeast-based prebiotic-type compounds as potential feed additives. Front. Vet. Sci. 2:28. 10.3389/fvets.2015.00028 26664957 PMC4672232

[B53] Ryll M. Christensen H. Bisgaard M. Christensen J. P. Hinz K. H. Köhler B. (2008). Studies on the prevalence of riemerella anatipestifer in the upper respiratory tract of clinically healthy ducklings and characterization of untypable strains. J. Vet. Med. Ser. B 48, 537–546. 10.1111/j.1439-0450.2001.00471.x 11666036

[B54] Schenk A. Porter A. L. Alenciks E. Frazier K. Best A. A. Fraley S. M.. (2016). Increased water contamination and grow-out Pekin duck mortality when raised with water troughs compared to pin-metered water lines using a United States management system. Poult. Sci. 95, 736–748. 10.3382/ps/pev381 26769272 PMC4957366

[B55] Scupham A. J. Patton T. G. Bent E. Bayles D. O. Scupham A. J. Patton T. G.. (2008). Comparison of the cecal microbiota of domestic and wild turkeys. Microb. Ecol. 56, 322–331. 10.1007/s00248-007-9349-4 18183454

[B56] Shibata H. Sogou M. (1982). [Gastrointestinal transit in the chicken using 198Au-colloid as a marker (author's transl)]. Radioisotopes 31, 82–87. 6283603

[B57] Soergel D. A. W. Dey N. Knight R. Brenner S. E. (2012). Selection of primers for optimal taxonomic classification of environmental 16S rRNA gene sequences. ISME J. 6, 1440–1444. 10.1038/ismej.2011.208 22237546 PMC3379642

[B58] Stanley D. Geier M. S. Hughes R. J. Denman S. E. Moore R. J. (2013). Highly variable microbiota development in the chicken gastrointestinal tract. PLoS ONE 8, 6–12. 10.1371/journal.pone.0084290 24391931 PMC3877270

[B59] Stanley D. Hughes R. J. Moore R. J. (2014). Microbiota of the chicken gastrointestinal tract: influence on health, productivity and disease. Appl. Microbiol. Biotechnol. 98, 4301–4310. 10.1007/s00253-014-5646-2 24643736

[B60] Tims S. Derom C. Jonkers D. M. Vlietinck R. Saris W. H. Kleerebezem M.. (2013). Microbiota conservation and BMI signatures in adult monozygotic twins. ISME J. 7, 707–717. 10.1038/ismej.2012.146 23190729 PMC3603393

[B61] Turnbaugh P. J. Ley R. E. Mahowald M. A. Magrini V. Mardis E. R. Gordon J. I. (2006). An obesity-associated gut microbiome with increased capacity for energy harvest. Nature 444, 1027–1031. 10.1038/nature05414 17183312

[B62] van der Wielen P. W. Keuzenkamp D. A. Lipman L. J. Van Knapen F. Biesterveld S. (2002). Spatial and temporal variation of the intestinal bacterial community in commercially raised broiler chickens during growth. Microb. Ecol. 44, 286–293. 10.1007/s00248-002-2015-y 12219265

[B63] Van Immerseel F. De Buck J. Pasmans F. Huyghebaert G. Haesebrouck F. Ducatelle R.. (2004). Clostridium perfringens in poultry: an emerging threat for animal and public health. Avian Pathol. 33, 537–549. 10.1080/03079450400013162 15763720

[B64] Vasaï F. Brugirard Ricaud K. Bernadet M. D. Cauquil L. Bouchez O. Combes S.. (2014). Overfeeding and genetics affect the composition of intestinal microbiota in *Anas platyrhynchos* (Pekin) and *Cairina moschata* (Muscovy) ducks. FEMS Microbiol. Ecol. 87, 204–216. 10.1111/1574-6941.12217 24102552

[B65] Vázquez-Baeza Y. Pirrung M. Gonzalez A. Knight R. (2013). EMPeror: a tool for visualizing high-throughput microbial community data. Gigascience 2:16. 10.1186/2047-217X-2-16 24280061 PMC4076506

[B66] Waite D. W. Taylor M. W. (2014). Characterizing the avian gut microbiota: membership, driving influences, and potential function. Front. Microbiol. 5:223. 10.3389/fmicb.2014.00223 24904538 PMC4032936

[B67] Watkins B. A. Miller B. F. Neil D. H. (1982). *In vivo* inhibitory effects of *Lactobacillus acidophilus* against pathogenic *Escherichia coli* in gnotobiotic chicks. Poult. Sci. 61, 1298–1308. 10.3382/ps.0611298 6813835

[B68] Wei S. Morrison M. Yu Z. (2013). Bacterial census of poultry intestinal microbiome. Poult. Sci. 92, 671–683. 10.3382/ps.2012-02822 23436518

[B69] Westcott S. L. Schloss P. D. (2015). *De novo* clustering methods out-perform reference-based methods for assigning 16S rRNA gene sequences to operational taxonomic units. PeerJ 3:e1487 10.7717/peerj.1487 26664811 PMC4675110

[B70] Wobeser G. A. (1997). Diseases of Wild Waterfowl. Boston, MA: Springer US.

