# Supplementary material for: Characterization of Gut Microbiome Dynamics in Developing Pekin Ducks and Impact of Management System
Source: Front Microbiol. 2017 Jan 4;7:2125. doi: 10.3389/fmicb.2016.02125 (PMC5209349; doi:10.3389/fmicb.2016.02125)
Supplement: Supplementary file 6 [file DataSheet1.ZIP › Supplemental_File_1_AviaryStudy1_TaxaSummaries/charts/fKHTIJ7J0KxWet5gcqmpIKXzKDWTz3_legend.pdf]

NOHIT;Other;Other
k__Archaea;p__Crenarchaeota;c__Thaumarchaeota
k__Bacteria;p__Acidobacteria;c__BPC102
k__Bacteria;p__Acidobacteria;c__DA052
k__Bacteria;p__Actinobacteria;c__Acidimicrobiia
k__Bacteria;p__Actinobacteria;c__Actinobacteria
k__Bacteria;p__Actinobacteria;c__Coriobacteriia
k__Bacteria;p__Bacteroidetes;c__Bacteroidia
k__Bacteria;p__Bacteroidetes;c__Flavobacteriia
k__Bacteria;p__Bacteroidetes;c__Sphingobacteriia
k__Bacteria;p__Bacteroidetes;c__[Saprospirae]
k__Bacteria;p__Chlorobi;c__OPB56
k__Bacteria;p__Chloroflexi;c__Anaerolineae
k__Bacteria;p__Cyanobacteria;c__4C0d-2
k__Bacteria;p__Cyanobacteria;c__Synechococcophycideae
k__Bacteria;p__Deferribacteres;c__Deferribacteres
k__Bacteria;p__Firmicutes;c__Bacilli
k__Bacteria;p__Firmicutes;c__Clostridia
k__Bacteria;p__Firmicutes;c__Erysipelotrichi
k__Bacteria;p__Fusobacteria;c__Fusobacteriia
k__Bacteria;p__Gemmatimonadetes;c__Gemmatimonadetes
k__Bacteria;p__Planctomycetes;c__Planctomycetia
k__Bacteria;p__Proteobacteria;c__Alphaproteobacteria
k__Bacteria;p__Proteobacteria;c__Betaproteobacteria
k__Bacteria;p__Proteobacteria;c__Deltaproteobacteria
k__Bacteria;p__Proteobacteria;c__Epsilonproteobacteria
k__Bacteria;p__Proteobacteria;c__Gammaproteobacteria
k__Bacteria;p__Tenericutes;c__Mollicutes
