# Supplementary material for: Characterization of Gut Microbiome Dynamics in Developing Pekin Ducks and Impact of Management System
Source: Front Microbiol. 2017 Jan 4;7:2125. doi: 10.3389/fmicb.2016.02125 (PMC5209349; doi:10.3389/fmicb.2016.02125)
Supplement: Supplementary file 6 [file DataSheet1.ZIP › Supplemental_File_1_AviaryStudy1_TaxaSummaries/charts/OJqrfxWzroRz09ECNlHycxRU19DxKh_legend.pdf]

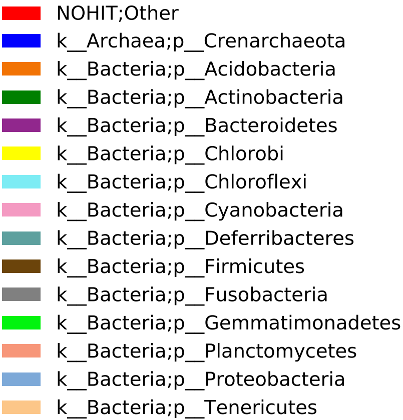
NOHIT;Other
k__Archaea;p__Crenarchaeota
k__Bacteria;p__Acidobacteria
k__Bacteria;p__Actinobacteria
k__Bacteria;p__Bacteroidetes
k__Bacteria;p__Chlorobi
k__Bacteria;p__Chloroflexi
k__Bacteria;p__Cyanobacteria
k__Bacteria;p__Deferribacteres
k__Bacteria;p__Firmicutes
k__Bacteria;p__Fusobacteria
k__Bacteria;p__Gemmatimonadetes
k__Bacteria;p__Planctomycetes
k__Bacteria;p__Proteobacteria
k__Bacteria;p__Tenericutes
