# Supplementary figures and images for: Characterization of Gut Microbiome Dynamics in Developing Pekin Ducks and Impact of Management System (part 1 of 2)
Source: Front Microbiol. 2017 Jan 4;7:2125. doi: 10.3389/fmicb.2016.02125 (PMC5209349; doi:10.3389/fmicb.2016.02125)

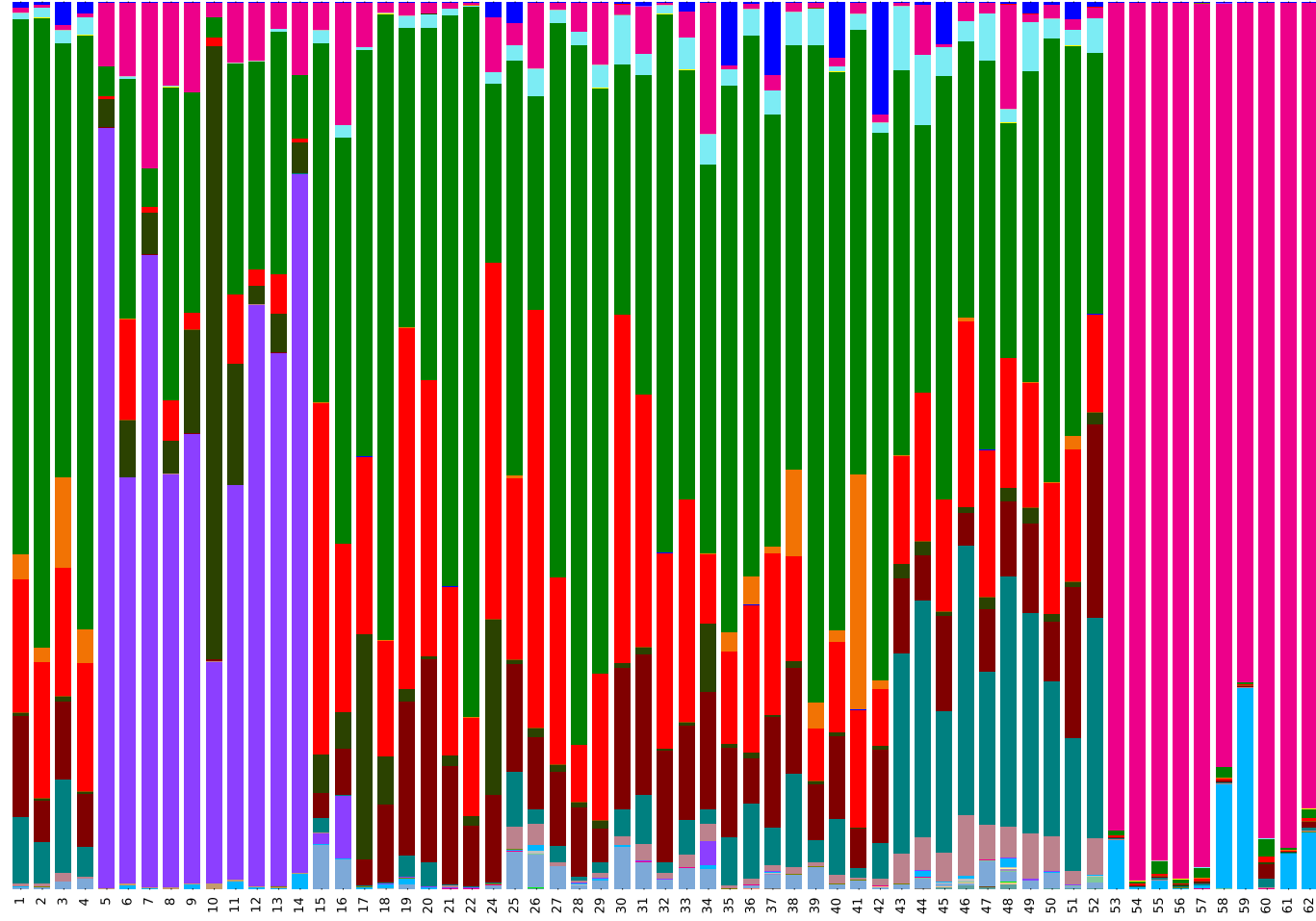

Supplement: Supplementary file 6 [file DataSheet1.ZIP › Supplemental_File_1_AviaryStudy1_TaxaSummaries/charts/1KTnglx4za0LJr8yWgZzDSadSZe817.pdf]

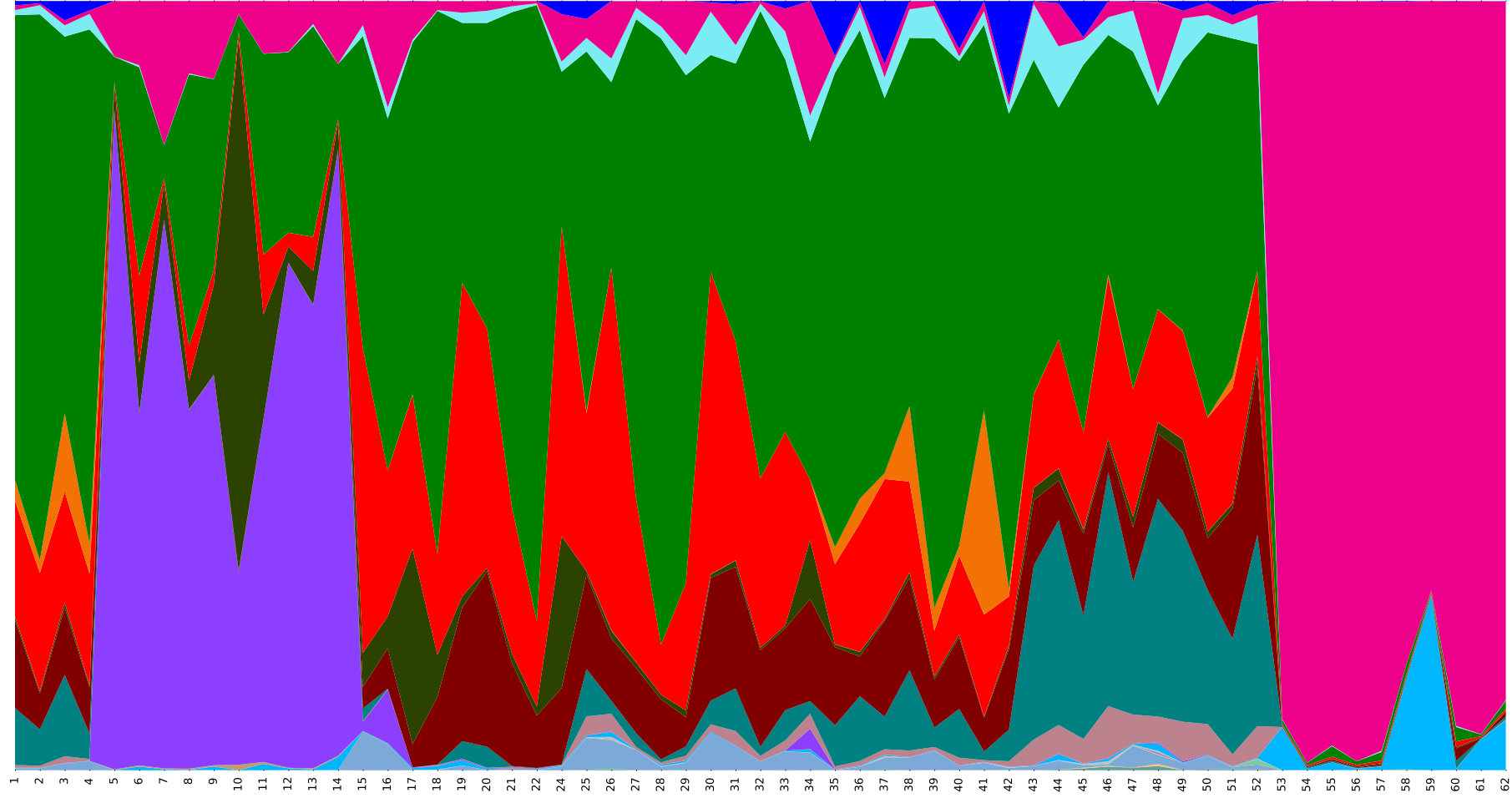

Supplement: Supplementary file 6 [file DataSheet1.ZIP › Supplemental_File_1_AviaryStudy1_TaxaSummaries/charts/7uRH9gIm0CcAsierBaBYGeURZqMZtz.pdf]

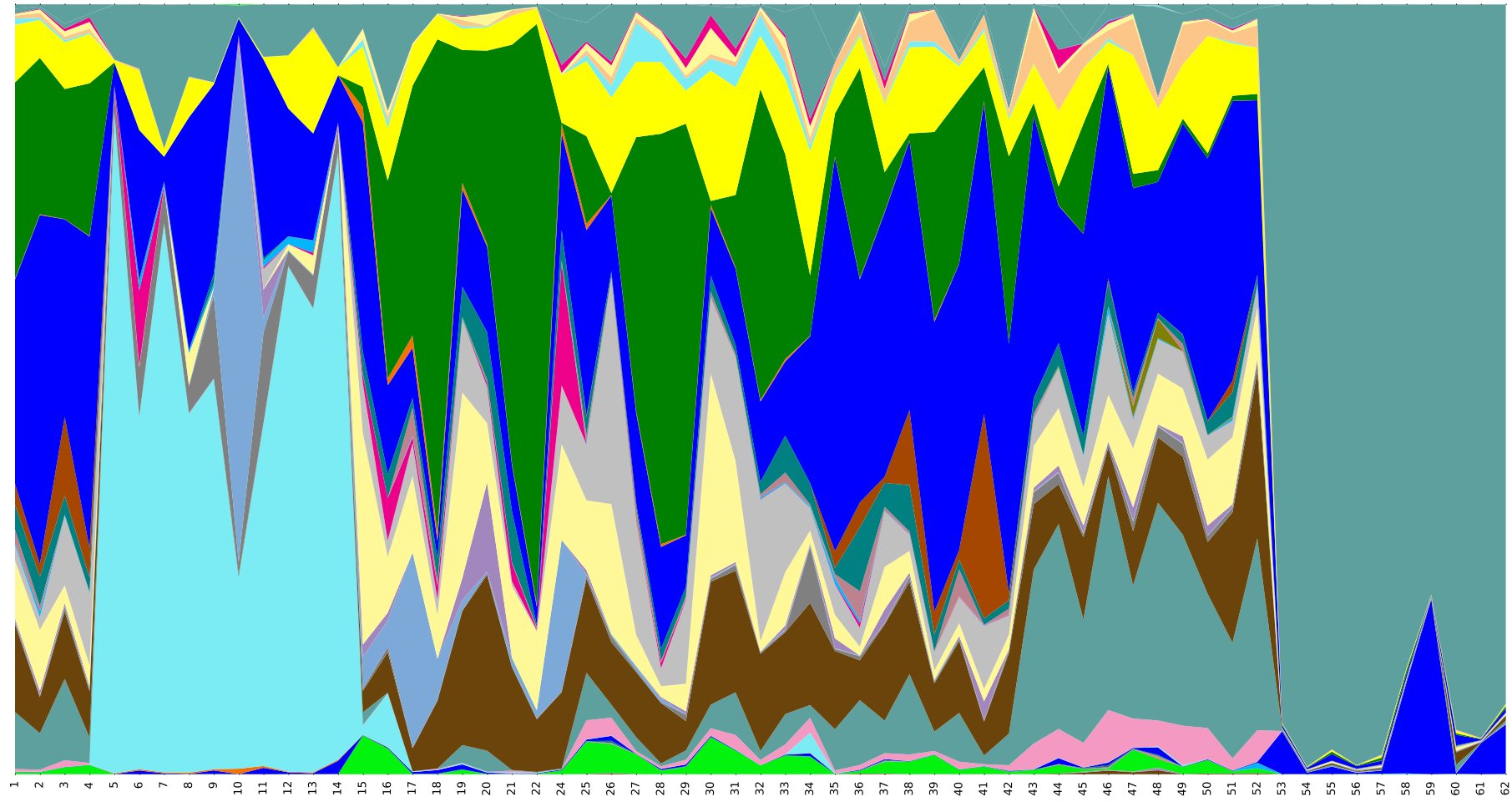

Supplement: Supplementary file 6 [file DataSheet1.ZIP › Supplemental_File_1_AviaryStudy1_TaxaSummaries/charts/8lOJjzjEZ3osiFA8x5fEKUTFZ0Mipl.pdf]

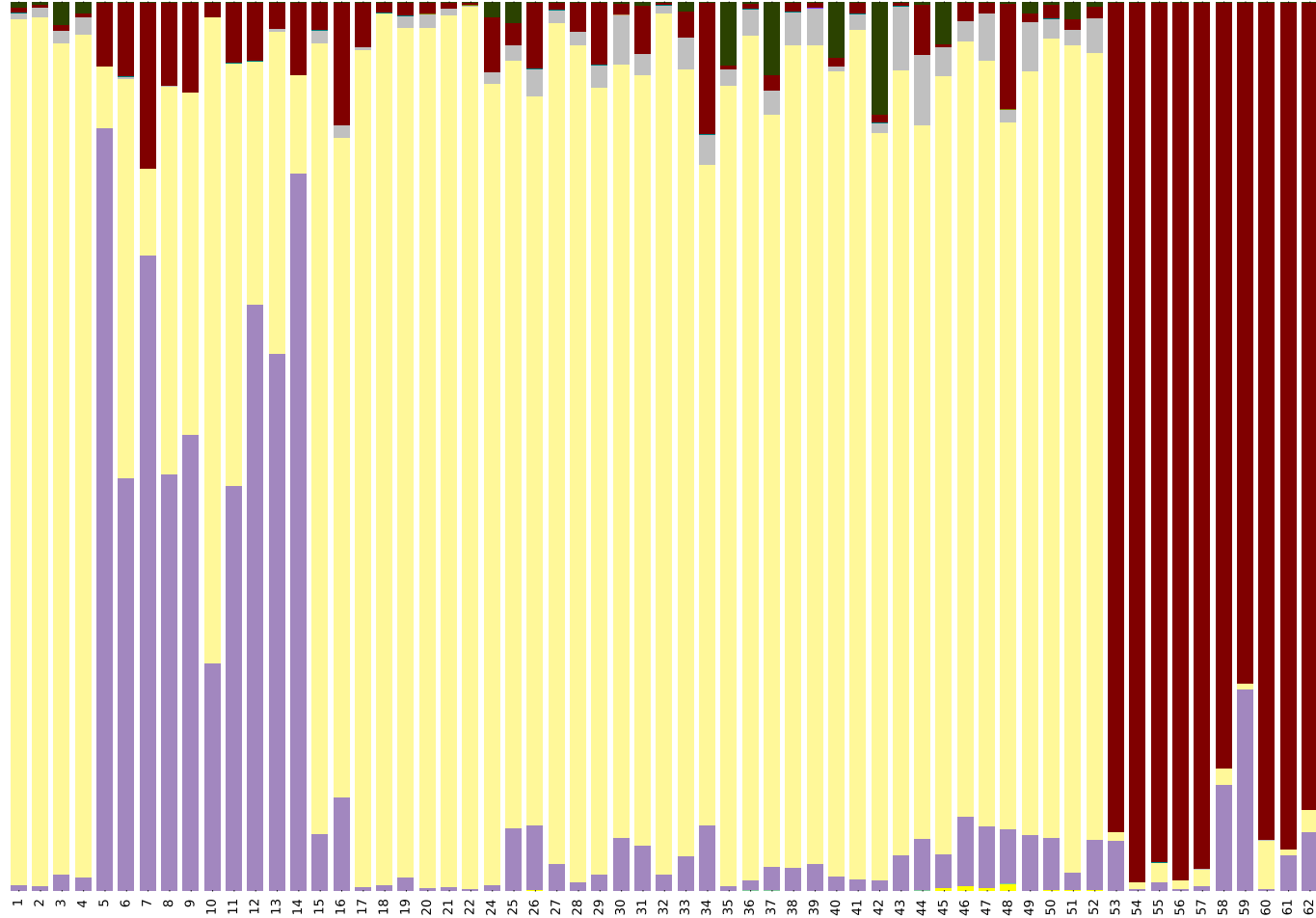

Supplement: Supplementary file 6 [file DataSheet1.ZIP › Supplemental_File_1_AviaryStudy1_TaxaSummaries/charts/aGzDHyIUCdNQsIO80m87QC9wdKM2TA.pdf]

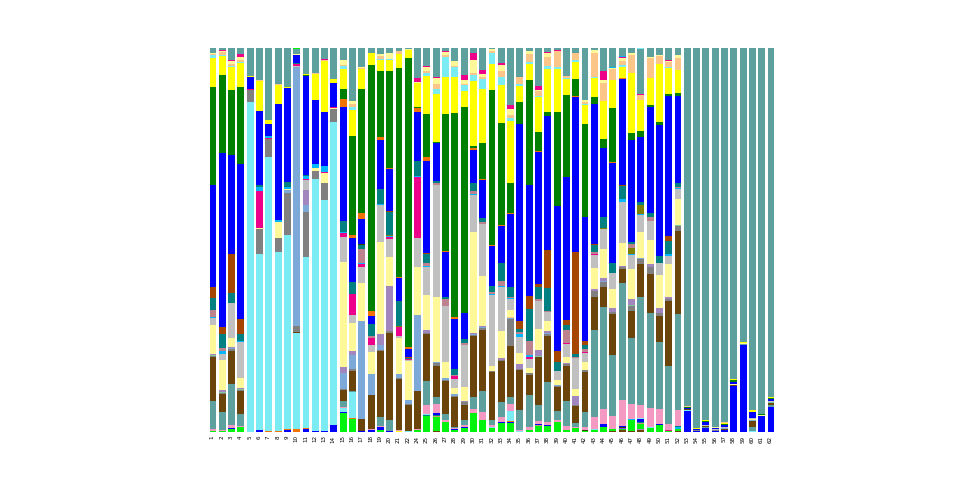

Supplement: Supplementary file 6 [file DataSheet1.ZIP › Supplemental_File_1_AviaryStudy1_TaxaSummaries/charts/aZxe7F0zKZxbfzOedsQpjH4wrUEAzl.png]

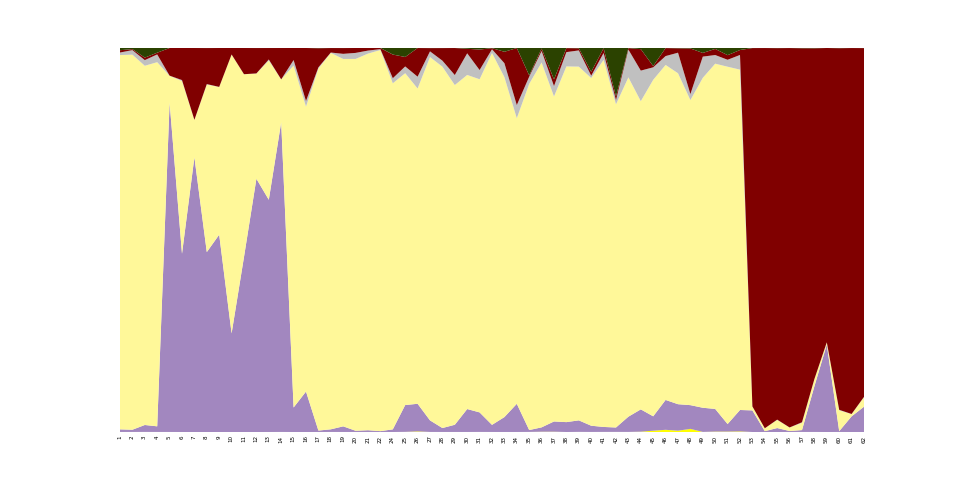

Supplement: Supplementary file 6 [file DataSheet1.ZIP › Supplemental_File_1_AviaryStudy1_TaxaSummaries/charts/fKHTIJ7J0KxWet5gcqmpIKXzKDWTz3.png]

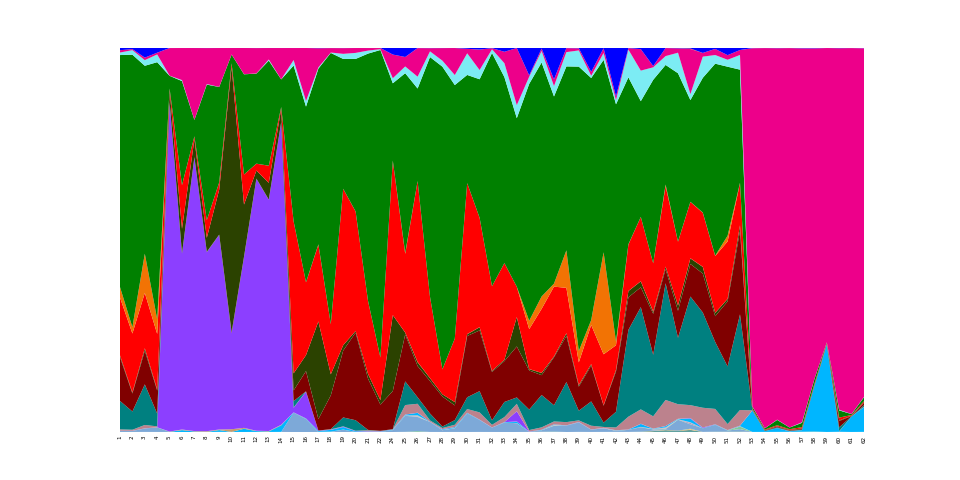

Supplement: Supplementary file 6 [file DataSheet1.ZIP › Supplemental_File_1_AviaryStudy1_TaxaSummaries/charts/G1ko2OFGDOjKe6ketUREdZOxNzyIzx.png]

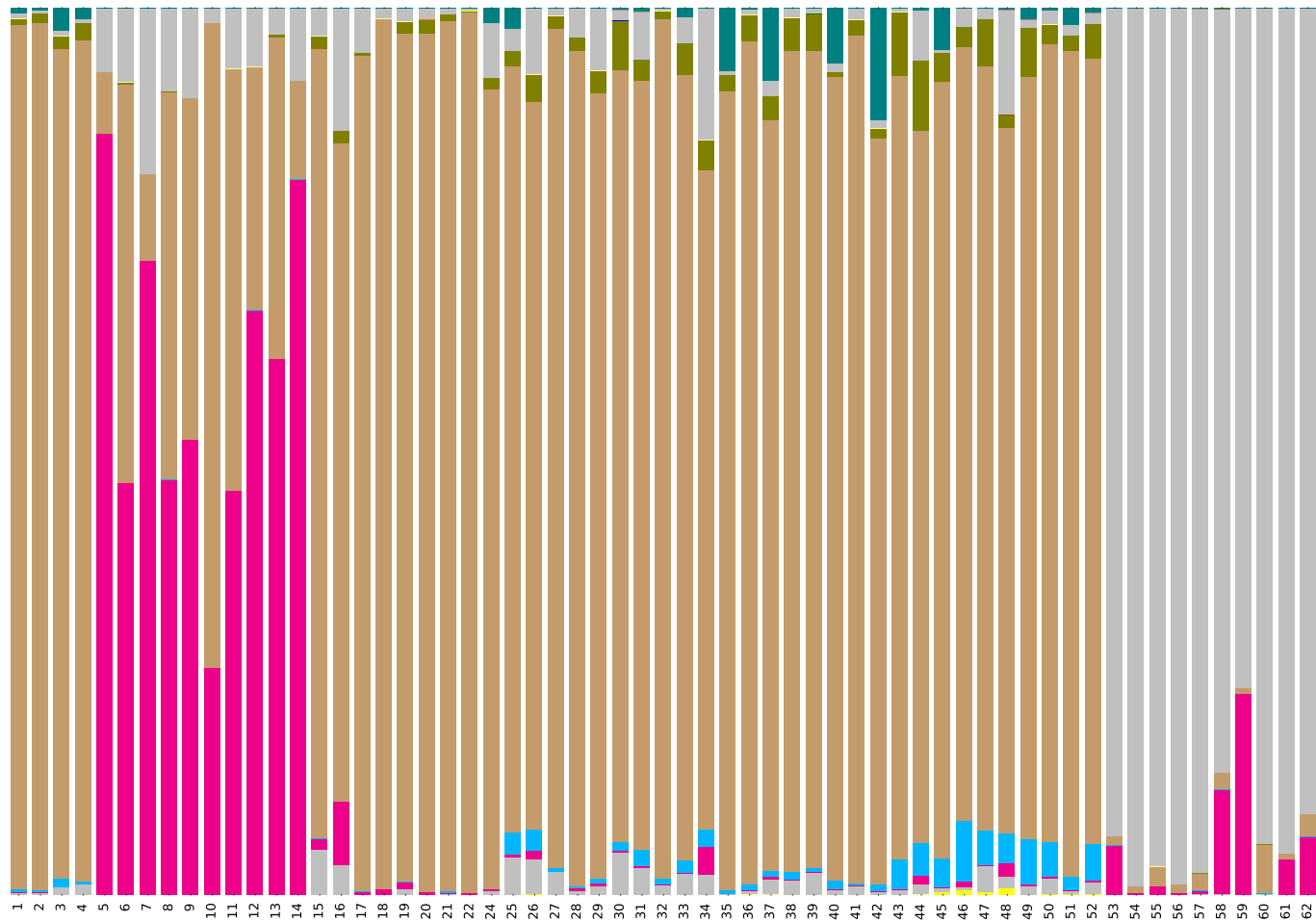

Supplement: Supplementary file 6 [file DataSheet1.ZIP › Supplemental_File_1_AviaryStudy1_TaxaSummaries/charts/g8xKXYzCkNNGdzFCdUS95m9nI6Zz4x.pdf]

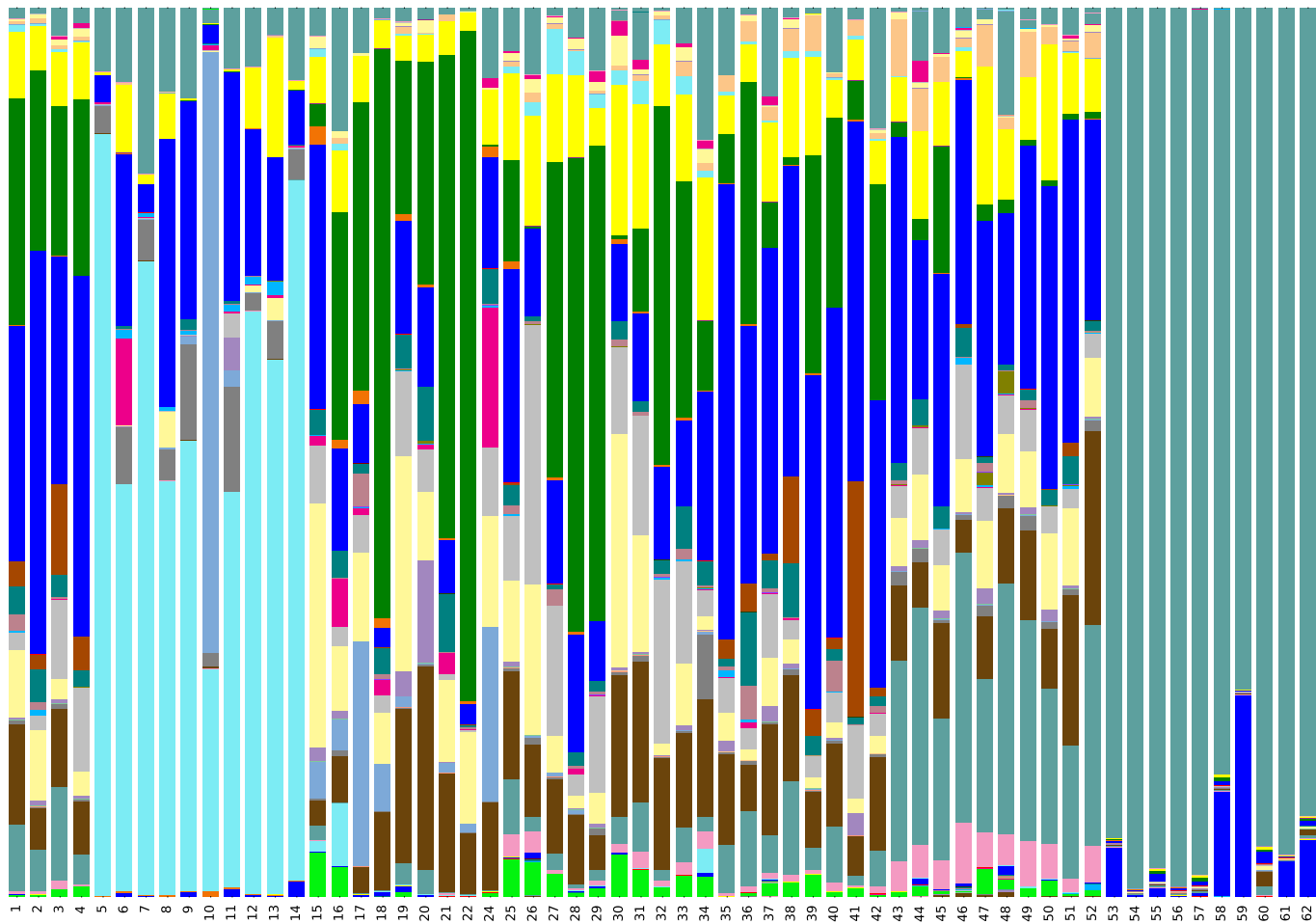

Supplement: Supplementary file 6 [file DataSheet1.ZIP › Supplemental_File_1_AviaryStudy1_TaxaSummaries/charts/MCni1wQbBWk0gPxBeE2oR8CmH0eUWD.pdf]

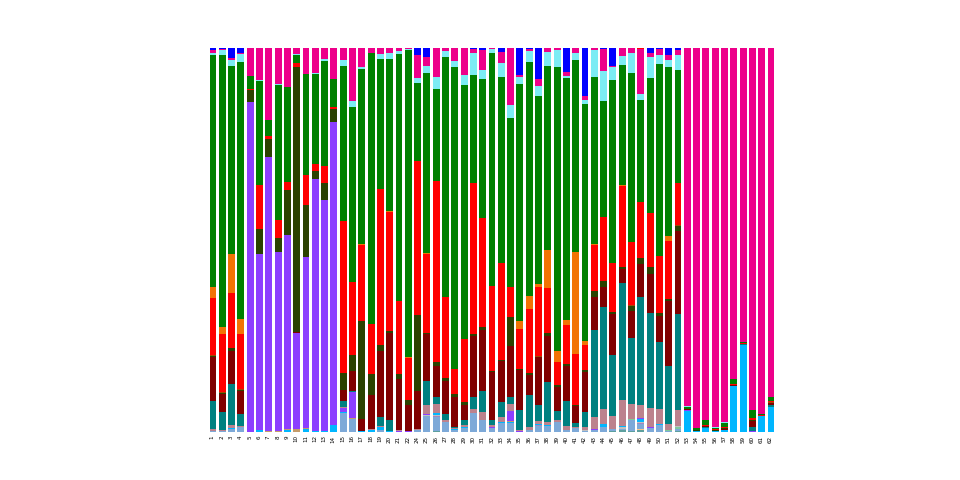

Supplement: Supplementary file 6 [file DataSheet1.ZIP › Supplemental_File_1_AviaryStudy1_TaxaSummaries/charts/MjkIazR8QLuUr1yJttyTUZceoyYkek.png]

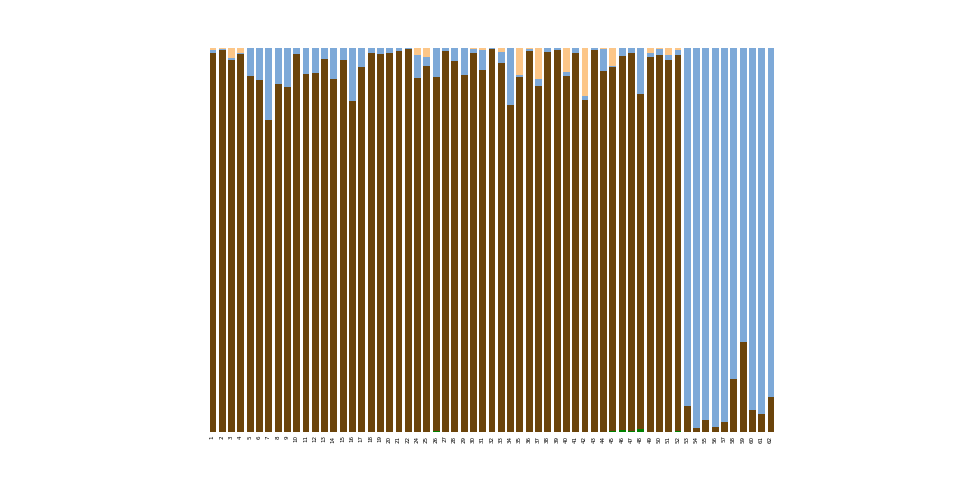

Supplement: Supplementary file 6 [file DataSheet1.ZIP › Supplemental_File_1_AviaryStudy1_TaxaSummaries/charts/OJqrfxWzroRz09ECNlHycxRU19DxKh.png]

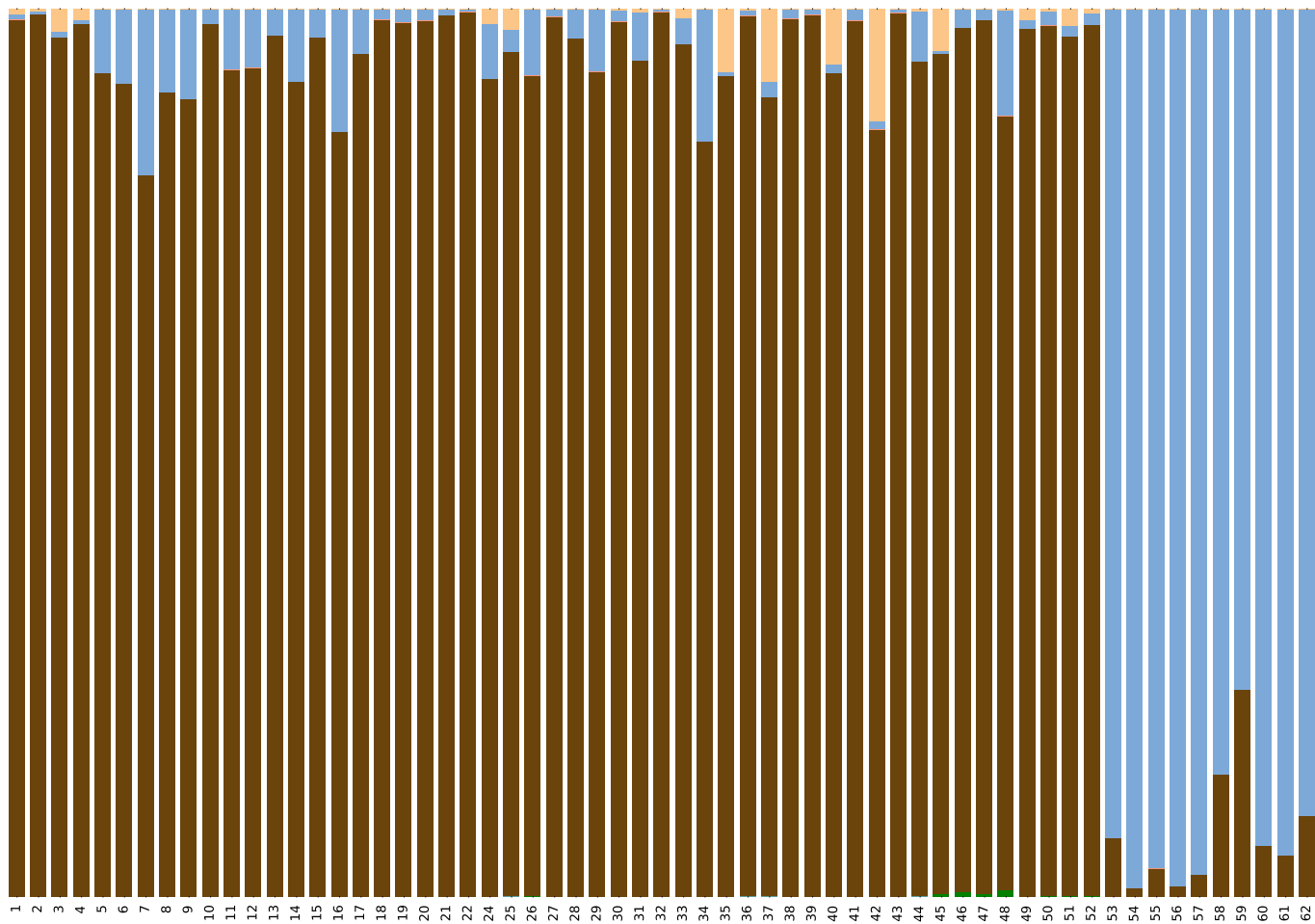

Supplement: Supplementary file 6 [file DataSheet1.ZIP › Supplemental_File_1_AviaryStudy1_TaxaSummaries/charts/PsqjzzTiSemznNa6iKIWO0z0Nk6dkZ.pdf]

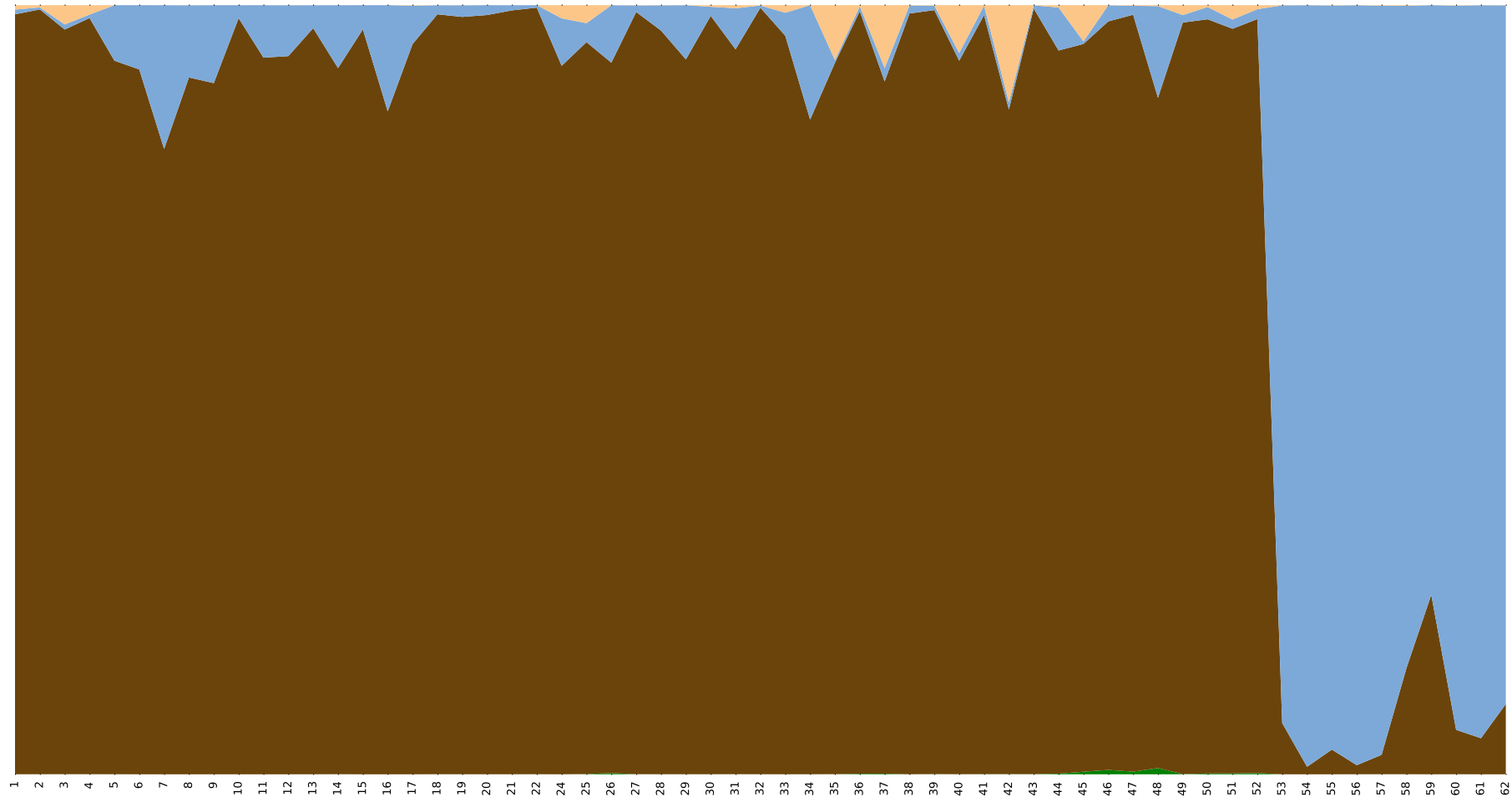

Supplement: Supplementary file 6 [file DataSheet1.ZIP › Supplemental_File_1_AviaryStudy1_TaxaSummaries/charts/pzeXlTrYcxng00U7uL33Ry6eIZtZ0y.pdf]

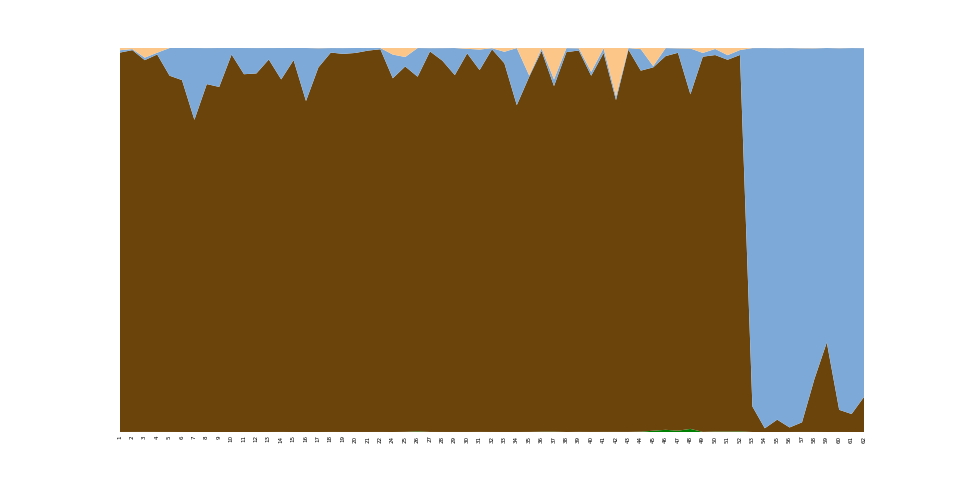

Supplement: Supplementary file 6 [file DataSheet1.ZIP › Supplemental_File_1_AviaryStudy1_TaxaSummaries/charts/pzg1lY9SZZUk3pMbuCp6yBCuXF2rCc.png]

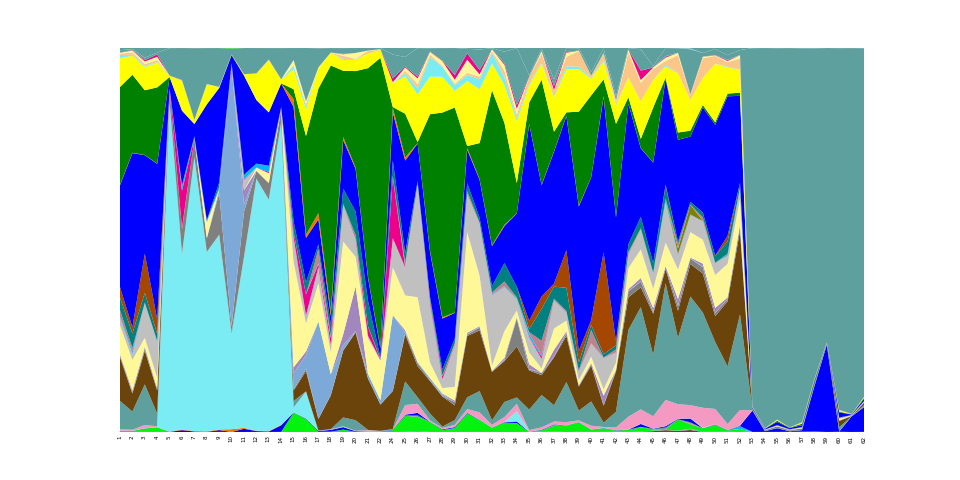

Supplement: Supplementary file 6 [file DataSheet1.ZIP › Supplemental_File_1_AviaryStudy1_TaxaSummaries/charts/sP2O0SwgerZ5YNTcuIBR5sr4Jh3xKa.png]

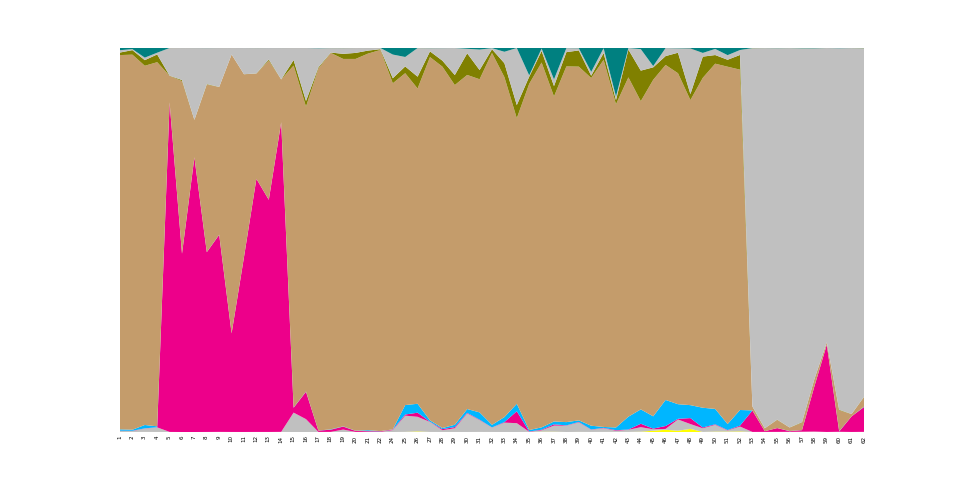

Supplement: Supplementary file 6 [file DataSheet1.ZIP › Supplemental_File_1_AviaryStudy1_TaxaSummaries/charts/TE1frk5w3QxHO7ZZp0ijYFMboZCoCd.png]

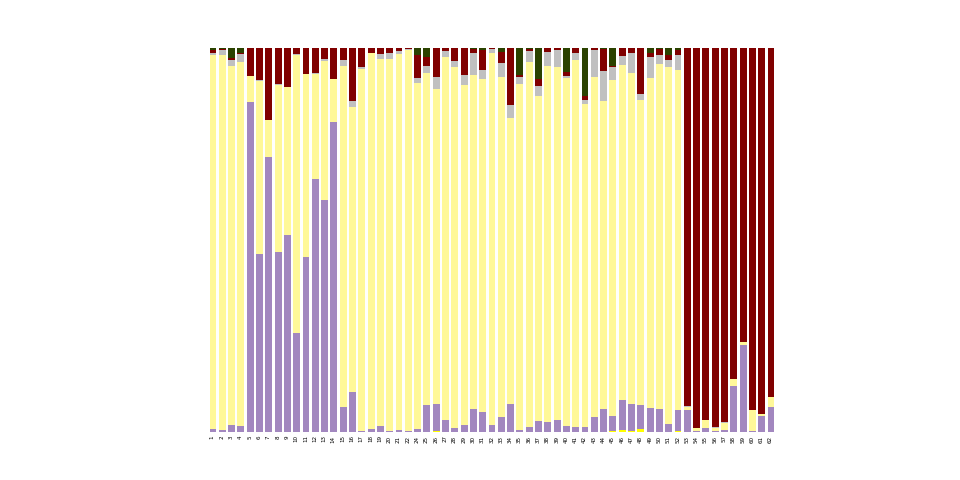

Supplement: Supplementary file 6 [file DataSheet1.ZIP › Supplemental_File_1_AviaryStudy1_TaxaSummaries/charts/Ua75umexZDAOr5xTCaK6ag53Qo73ps.png]

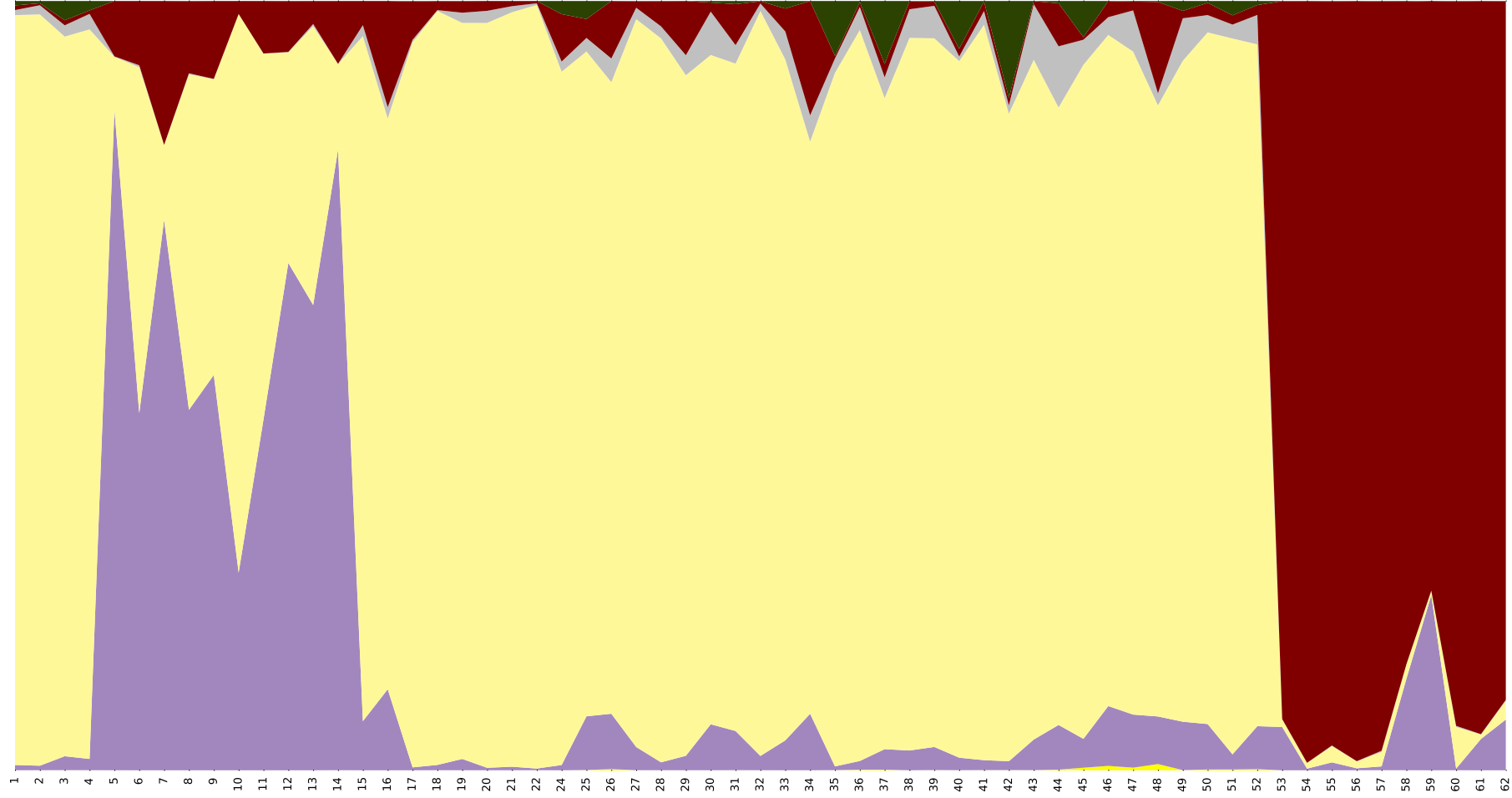

Supplement: Supplementary file 6 [file DataSheet1.ZIP › Supplemental_File_1_AviaryStudy1_TaxaSummaries/charts/uCW3mSGkhhABP658iO1AzgHpk4GzYM.pdf]

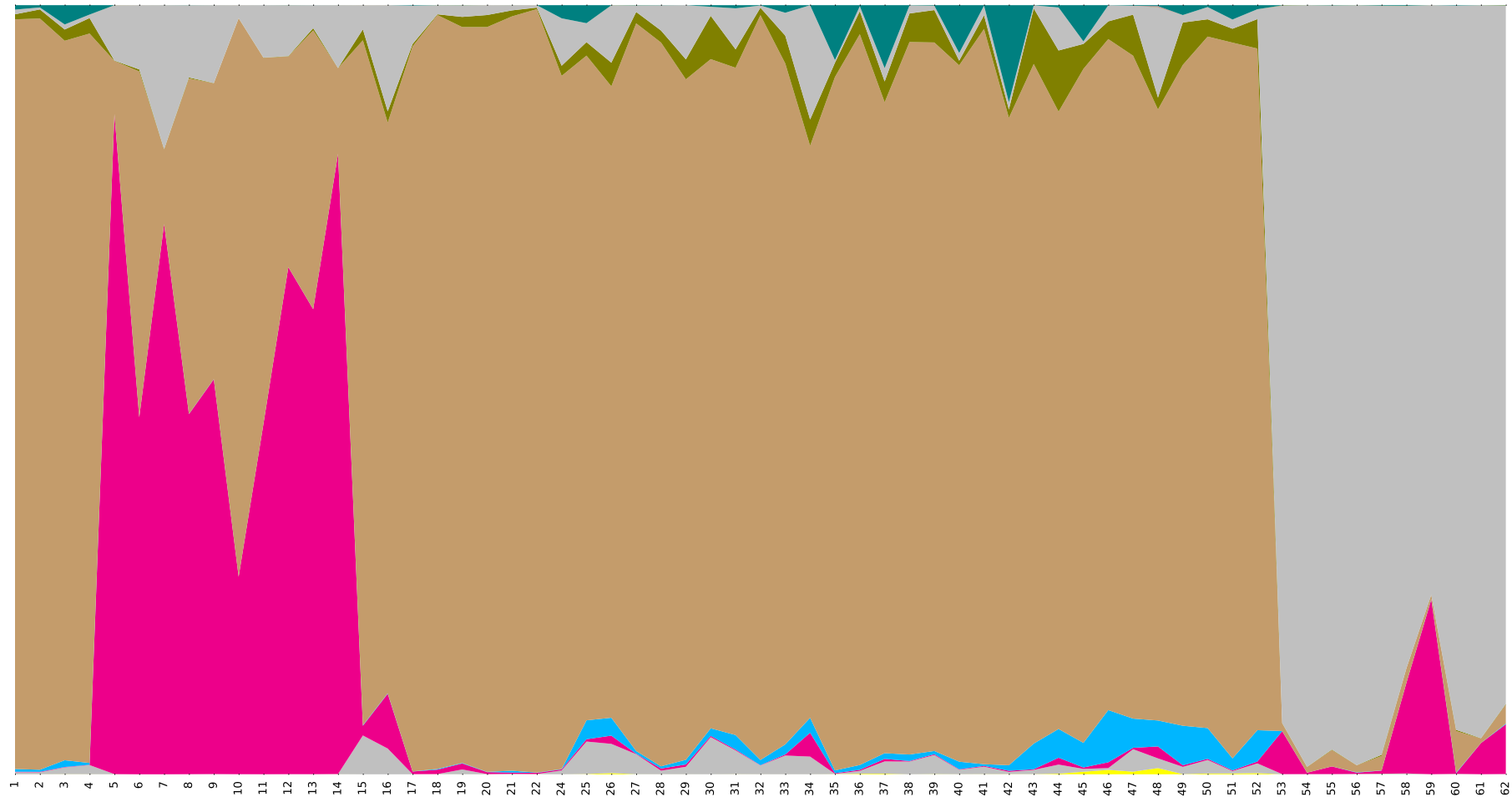

Supplement: Supplementary file 6 [file DataSheet1.ZIP › Supplemental_File_1_AviaryStudy1_TaxaSummaries/charts/UW11u49S7T95LK2MahEulz95yYZweZ.pdf]

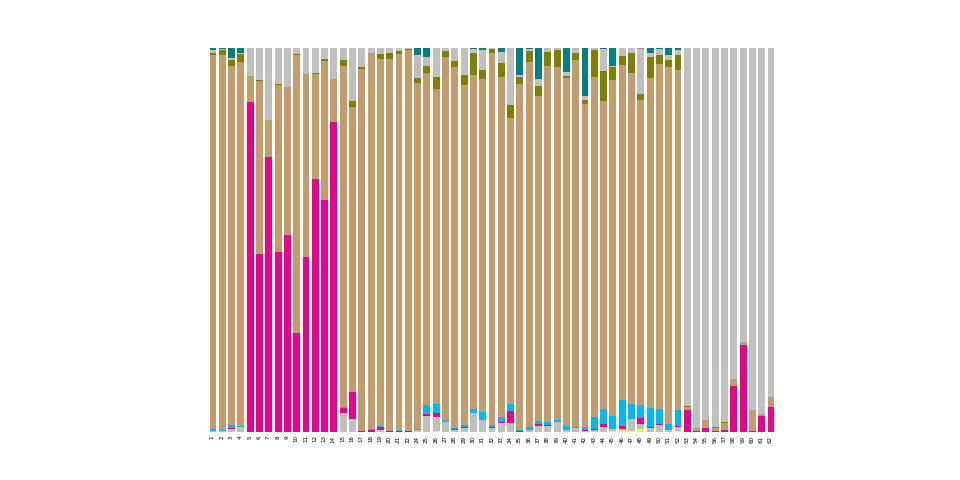

Supplement: Supplementary file 6 [file DataSheet1.ZIP › Supplemental_File_1_AviaryStudy1_TaxaSummaries/charts/zqZmOcz1xynAf4QB1N2UrzSN1FqZgM.png]

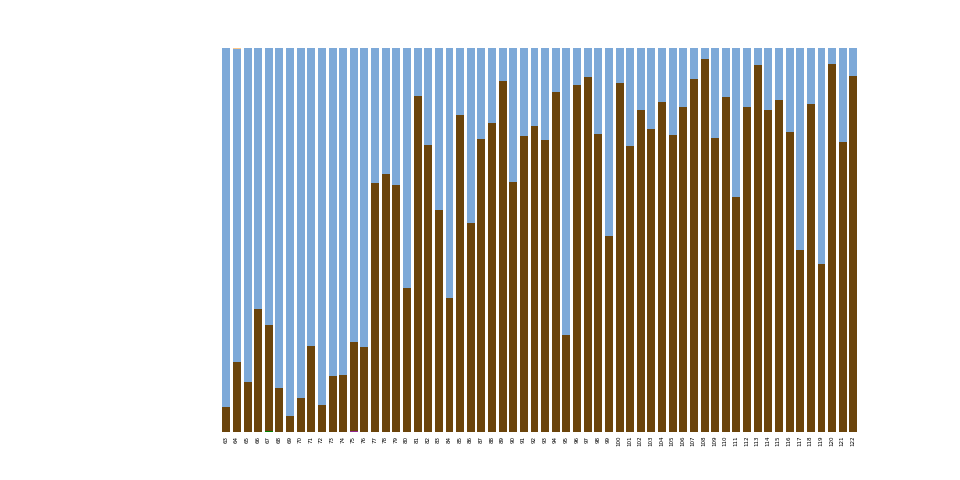

Supplement: Supplementary file 7 [file DataSheet2.ZIP › Supplemental_File_2_AviaryStudy2_TaxaSummaries/charts/47ZDh0AzcXwswz5EzzSZxlP4sFysrR.png]

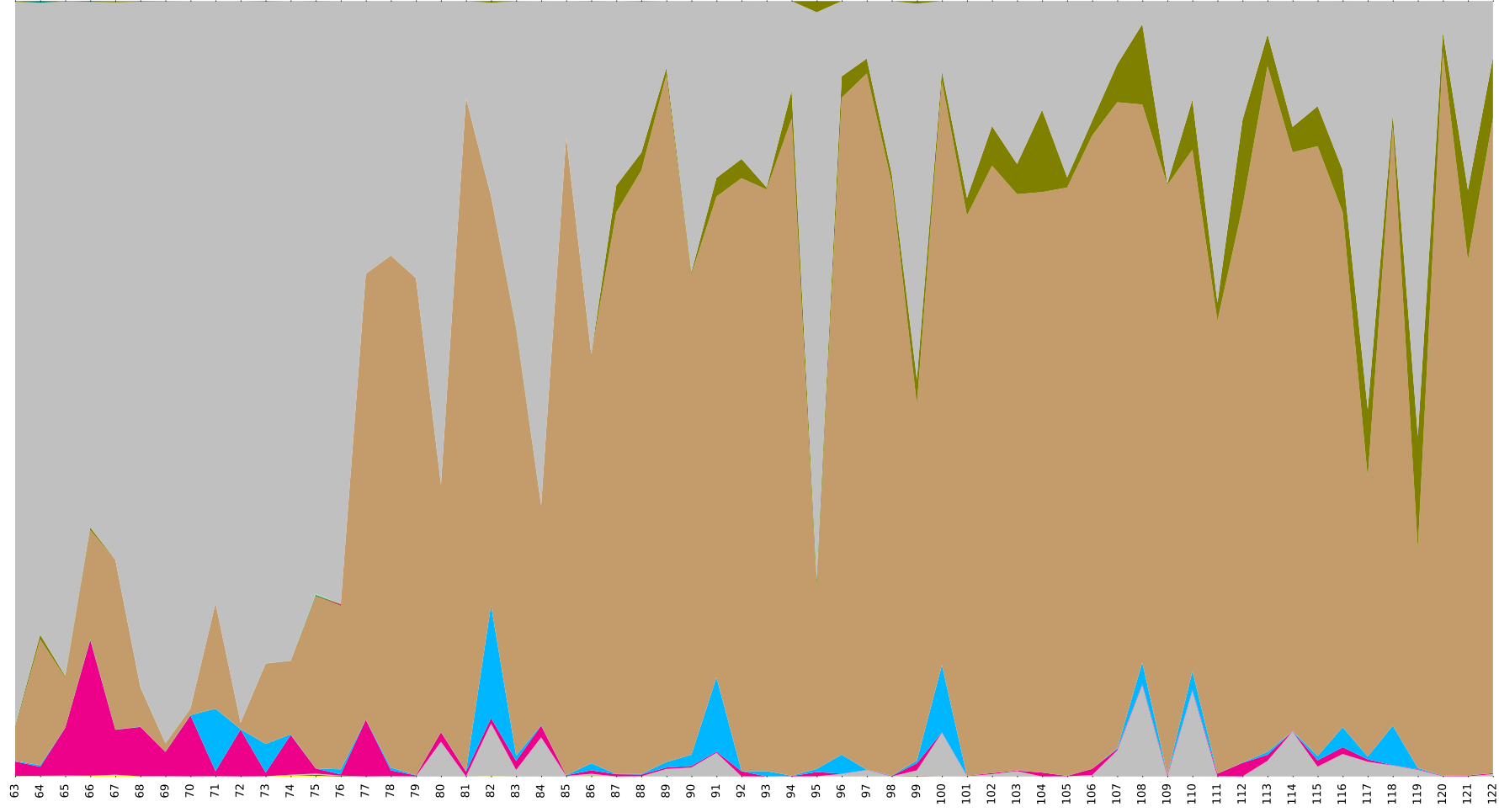

Supplement: Supplementary file 7 [file DataSheet2.ZIP › Supplemental_File_2_AviaryStudy2_TaxaSummaries/charts/53jLKZ2OUgu7ZPbZmzPzcdMzzDRA8x.pdf]

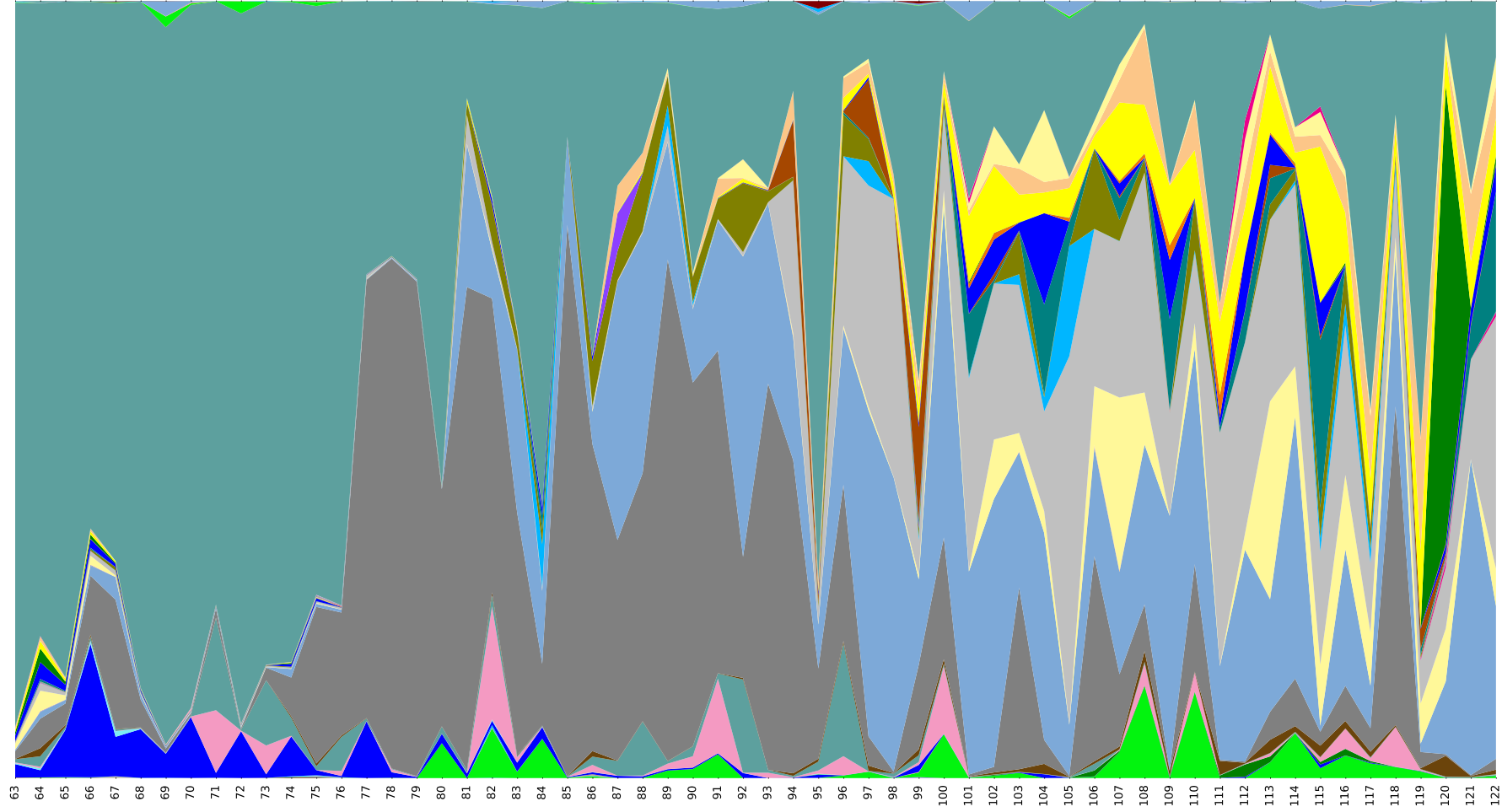

Supplement: Supplementary file 7 [file DataSheet2.ZIP › Supplemental_File_2_AviaryStudy2_TaxaSummaries/charts/6RmulM6ut7sJrgulMNUltFLrri0m9P.pdf]

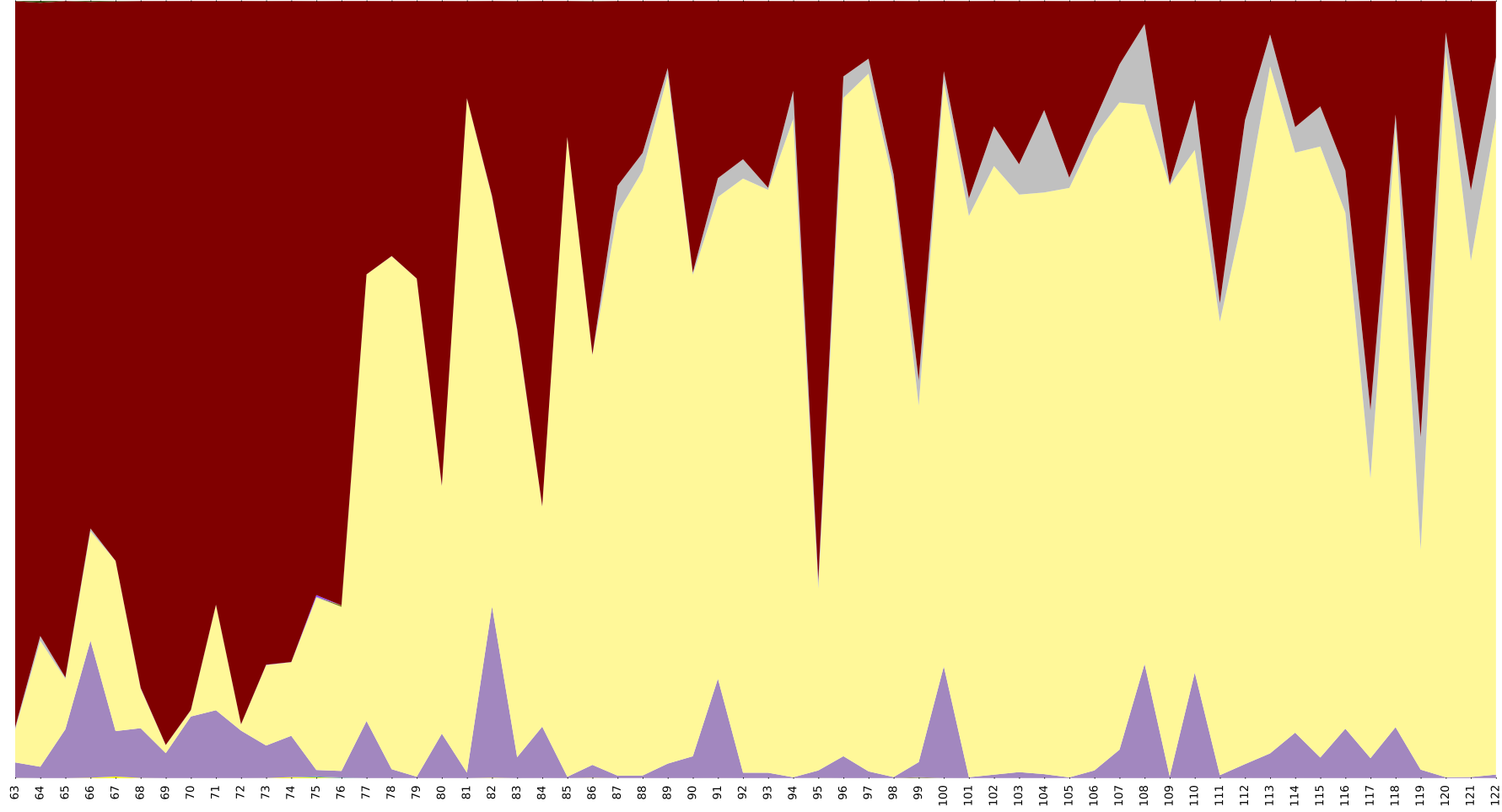

Supplement: Supplementary file 7 [file DataSheet2.ZIP › Supplemental_File_2_AviaryStudy2_TaxaSummaries/charts/7zRoHBHmHrorl93zZs1IzdYhJCZLfs.pdf]

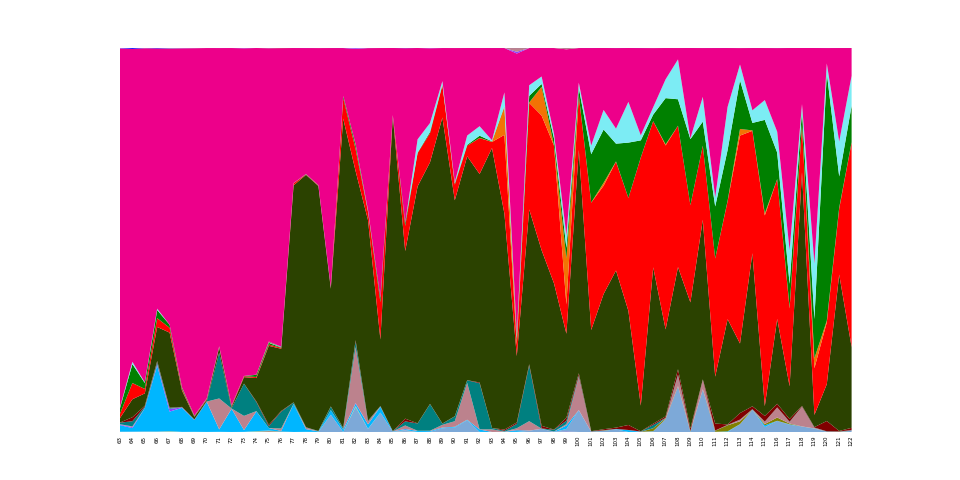

Supplement: Supplementary file 7 [file DataSheet2.ZIP › Supplemental_File_2_AviaryStudy2_TaxaSummaries/charts/8ZUtFLuxyb2zaP8D6H0q2sBa40cC0D.png]

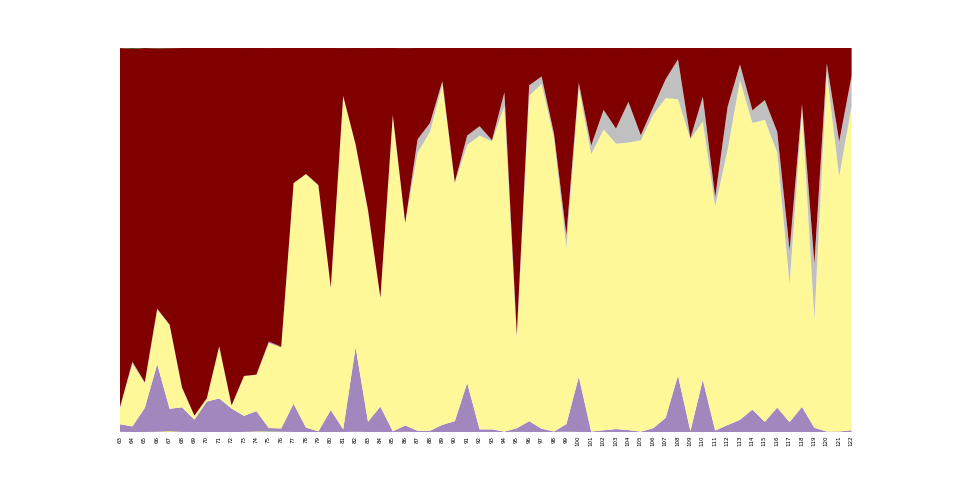

Supplement: Supplementary file 7 [file DataSheet2.ZIP › Supplemental_File_2_AviaryStudy2_TaxaSummaries/charts/AEoqbCMmCzummb7dRDhcgg0KhfW7bS.png]

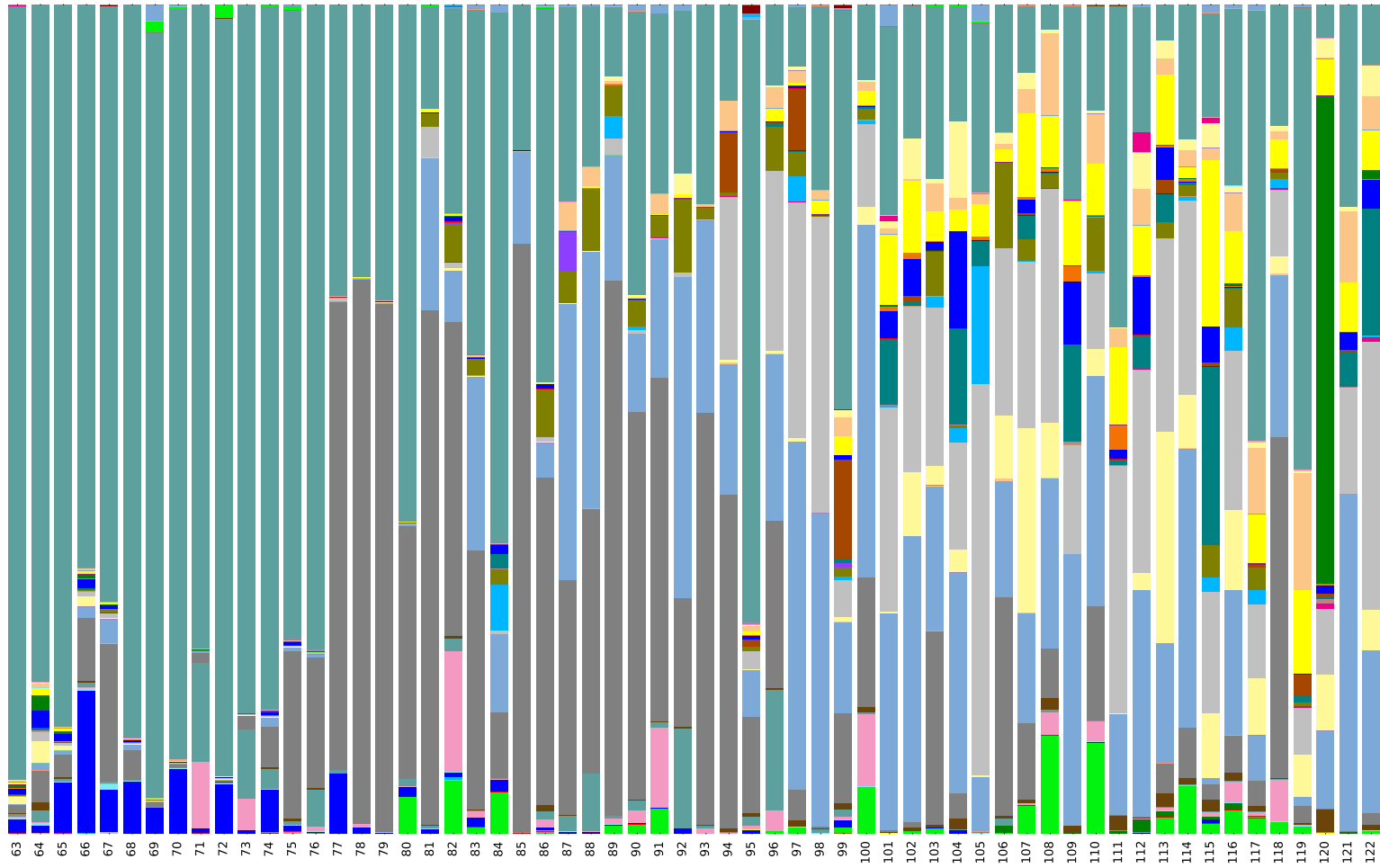

Supplement: Supplementary file 7 [file DataSheet2.ZIP › Supplemental_File_2_AviaryStudy2_TaxaSummaries/charts/BUkyGYyExk53842bfz0oCqSNcIMskz.pdf]

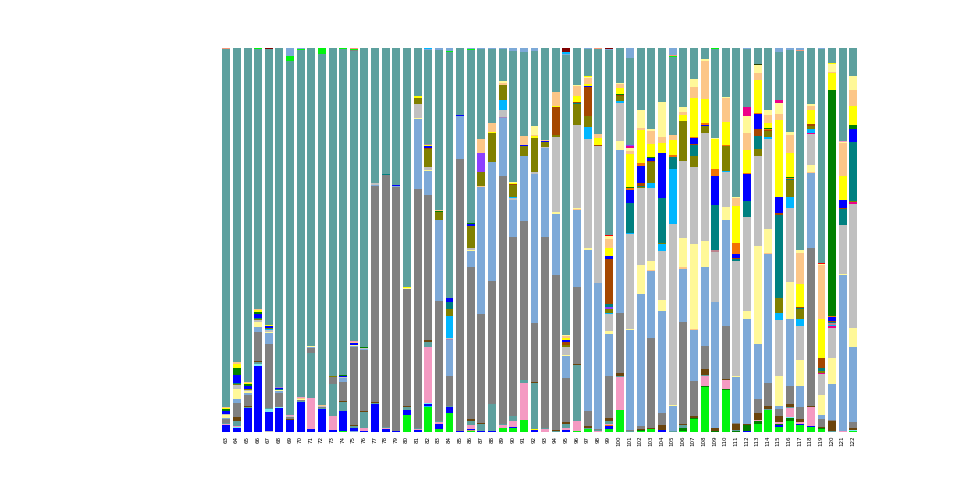

Supplement: Supplementary file 7 [file DataSheet2.ZIP › Supplemental_File_2_AviaryStudy2_TaxaSummaries/charts/cL5J2WlmtDP4HB1ScYZH9UtUtpeo99.png]

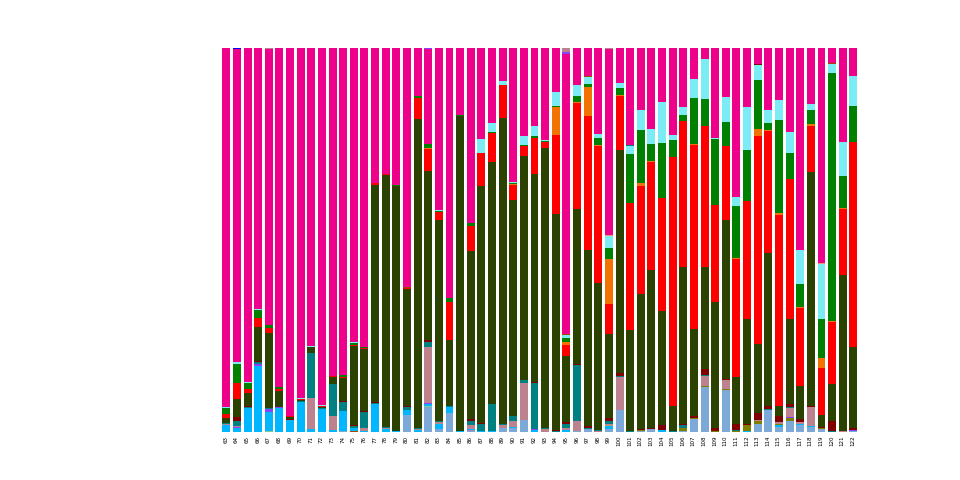

Supplement: Supplementary file 7 [file DataSheet2.ZIP › Supplemental_File_2_AviaryStudy2_TaxaSummaries/charts/FUr3aZHt48YW1i7qHIKQuyQgBz3rgn.png]

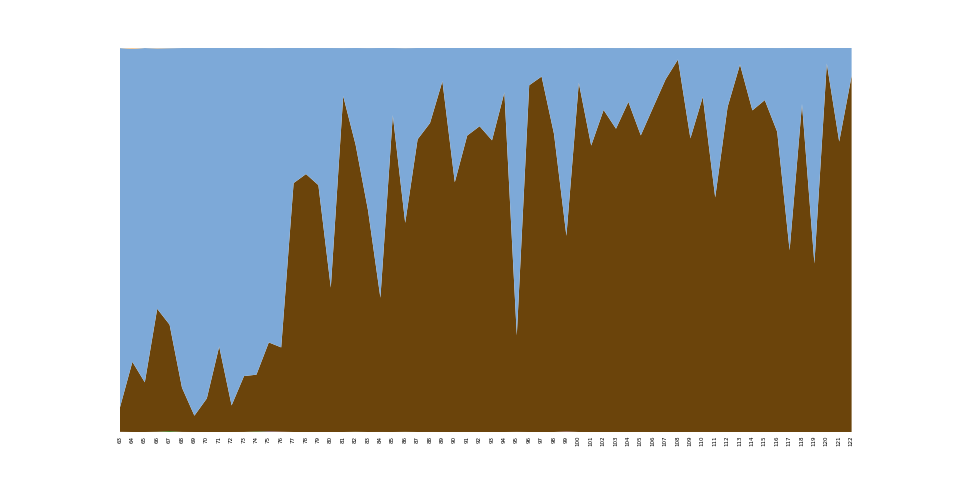

Supplement: Supplementary file 7 [file DataSheet2.ZIP › Supplemental_File_2_AviaryStudy2_TaxaSummaries/charts/nkrUEZpoidWz2OM0zlPIJsROMk0fyL.png]

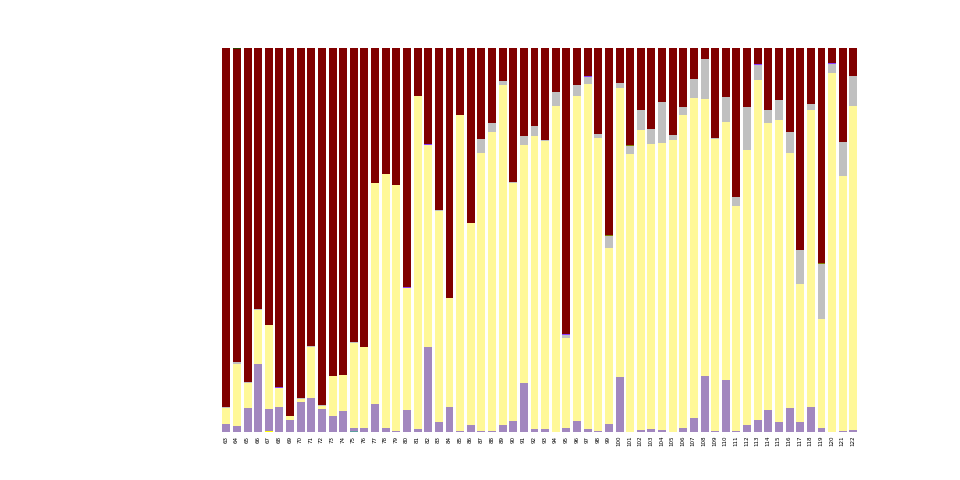

Supplement: Supplementary file 7 [file DataSheet2.ZIP › Supplemental_File_2_AviaryStudy2_TaxaSummaries/charts/Nqp2OQyNwbgmBQwT1lpCM7zEIL4ddH.png]

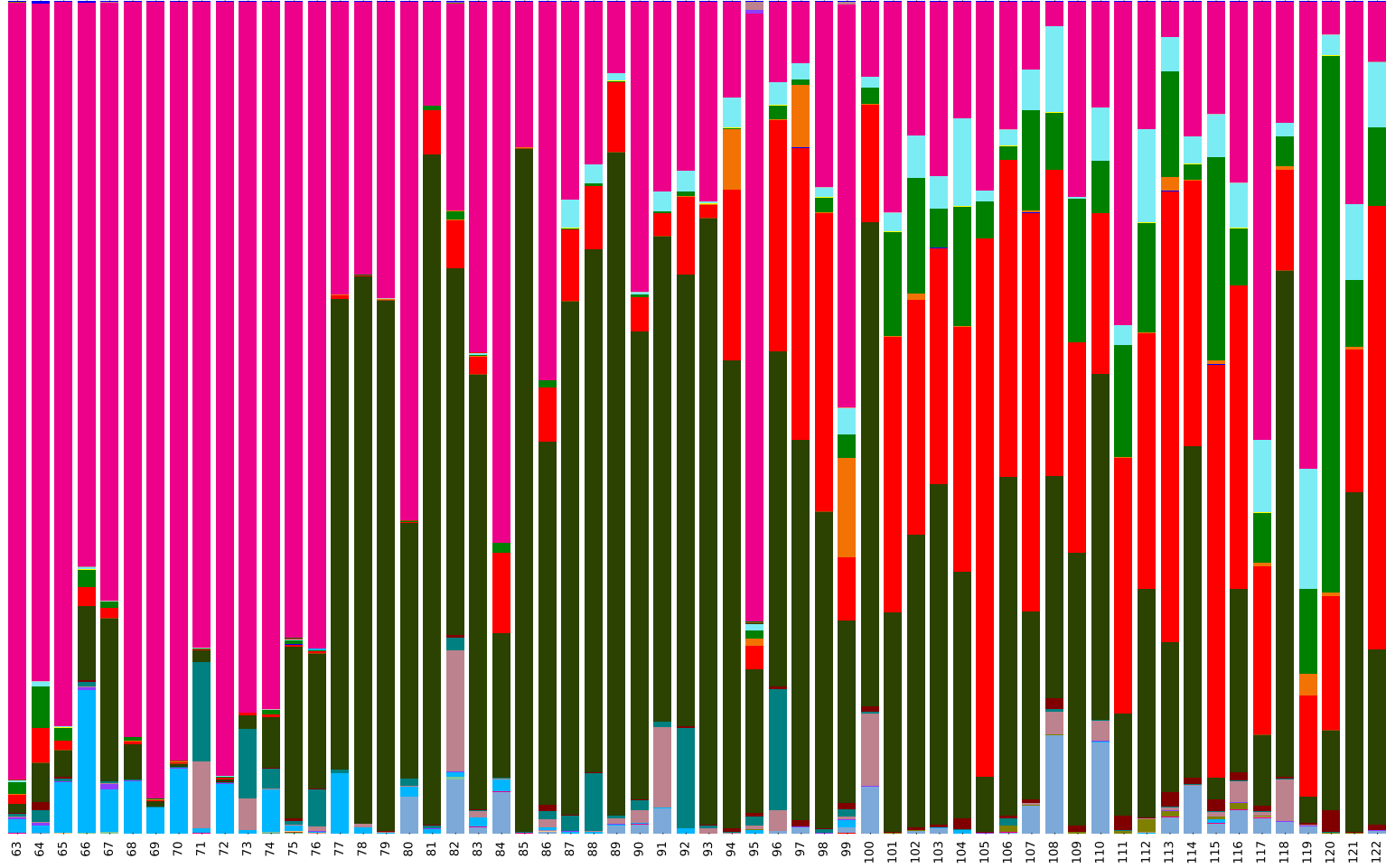

Supplement: Supplementary file 7 [file DataSheet2.ZIP › Supplemental_File_2_AviaryStudy2_TaxaSummaries/charts/NSDFZ00PUotK1kELkoi8n0IFw2waY2.pdf]

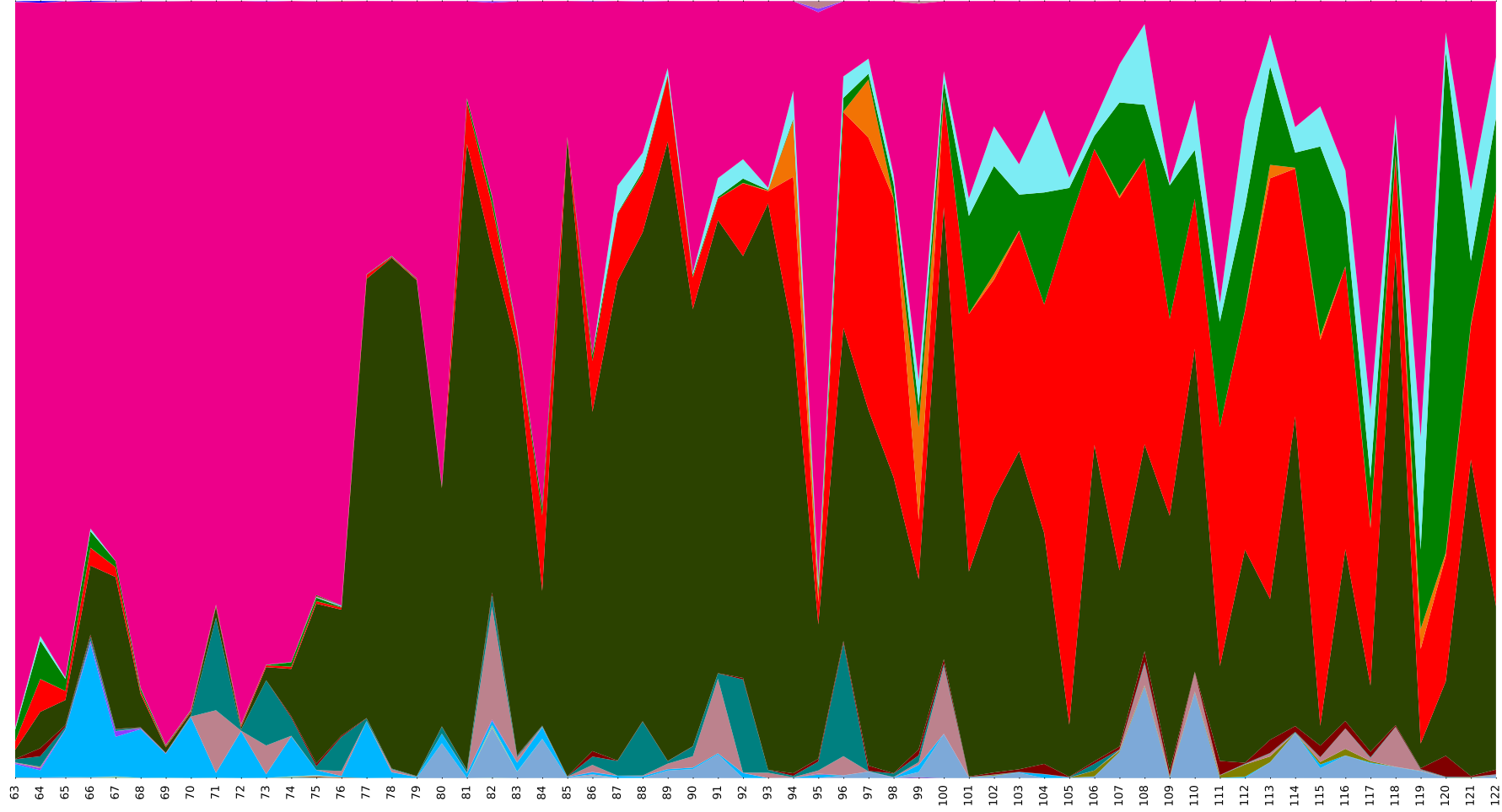

Supplement: Supplementary file 7 [file DataSheet2.ZIP › Supplemental_File_2_AviaryStudy2_TaxaSummaries/charts/qPF3w1zabgHWLhY7tCcYcW0tZq8eS6.pdf]

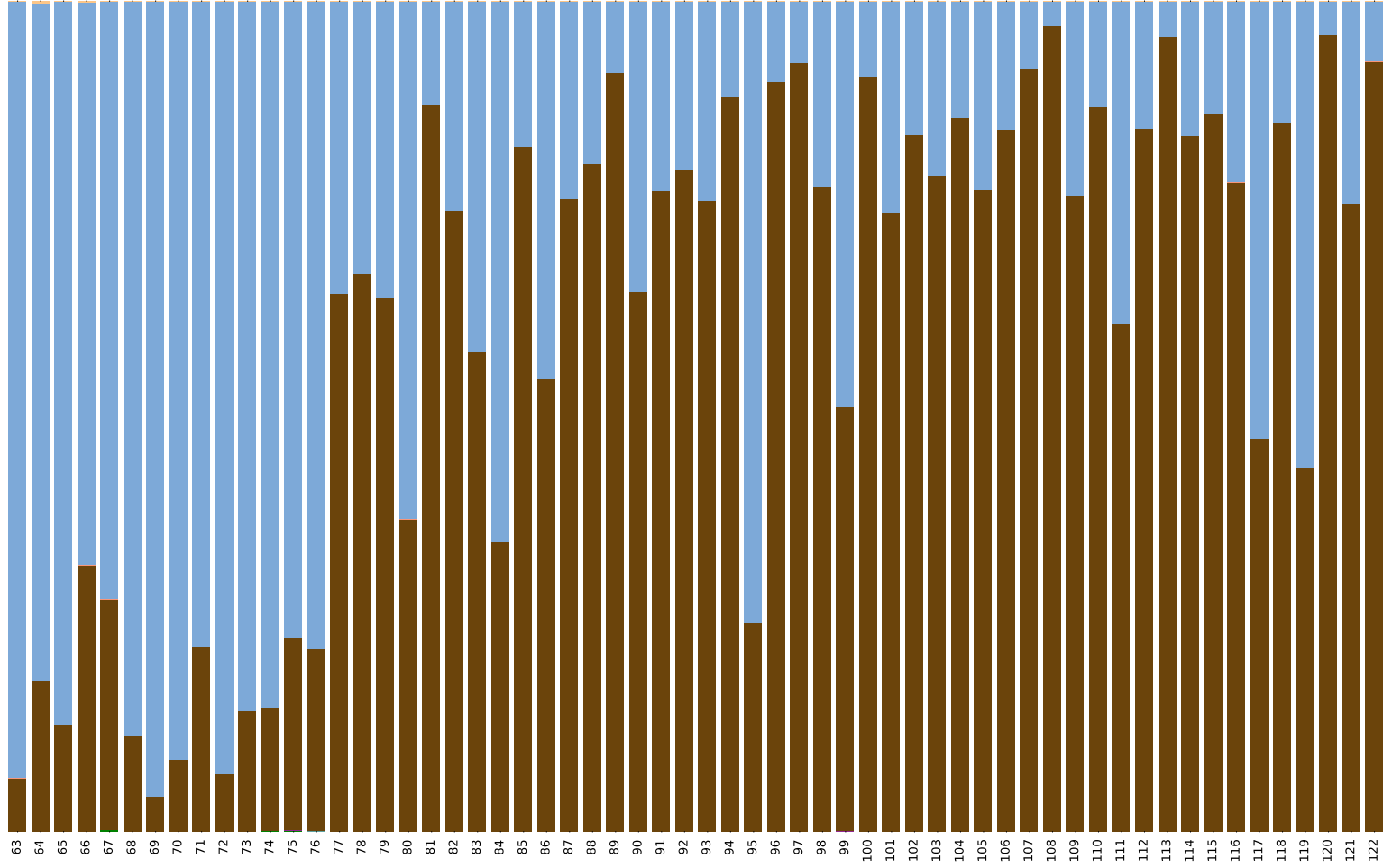

Supplement: Supplementary file 7 [file DataSheet2.ZIP › Supplemental_File_2_AviaryStudy2_TaxaSummaries/charts/RMyLpj7FP1U4HsBC9Pym70dPpIYeq5.pdf]

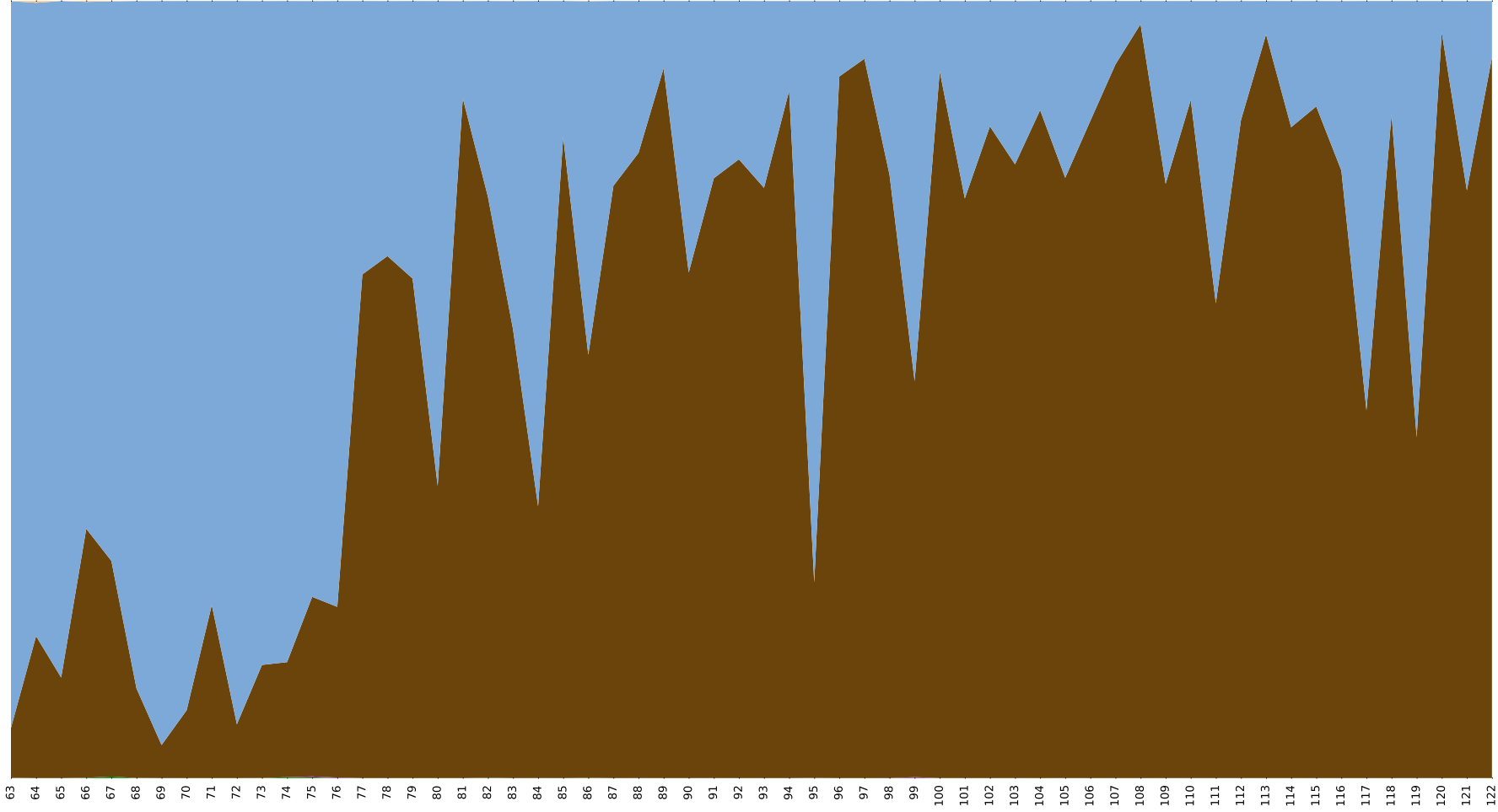

Supplement: Supplementary file 7 [file DataSheet2.ZIP › Supplemental_File_2_AviaryStudy2_TaxaSummaries/charts/sAsd0bzf1znWJ5iIJ30RANrGwptdQS.pdf]

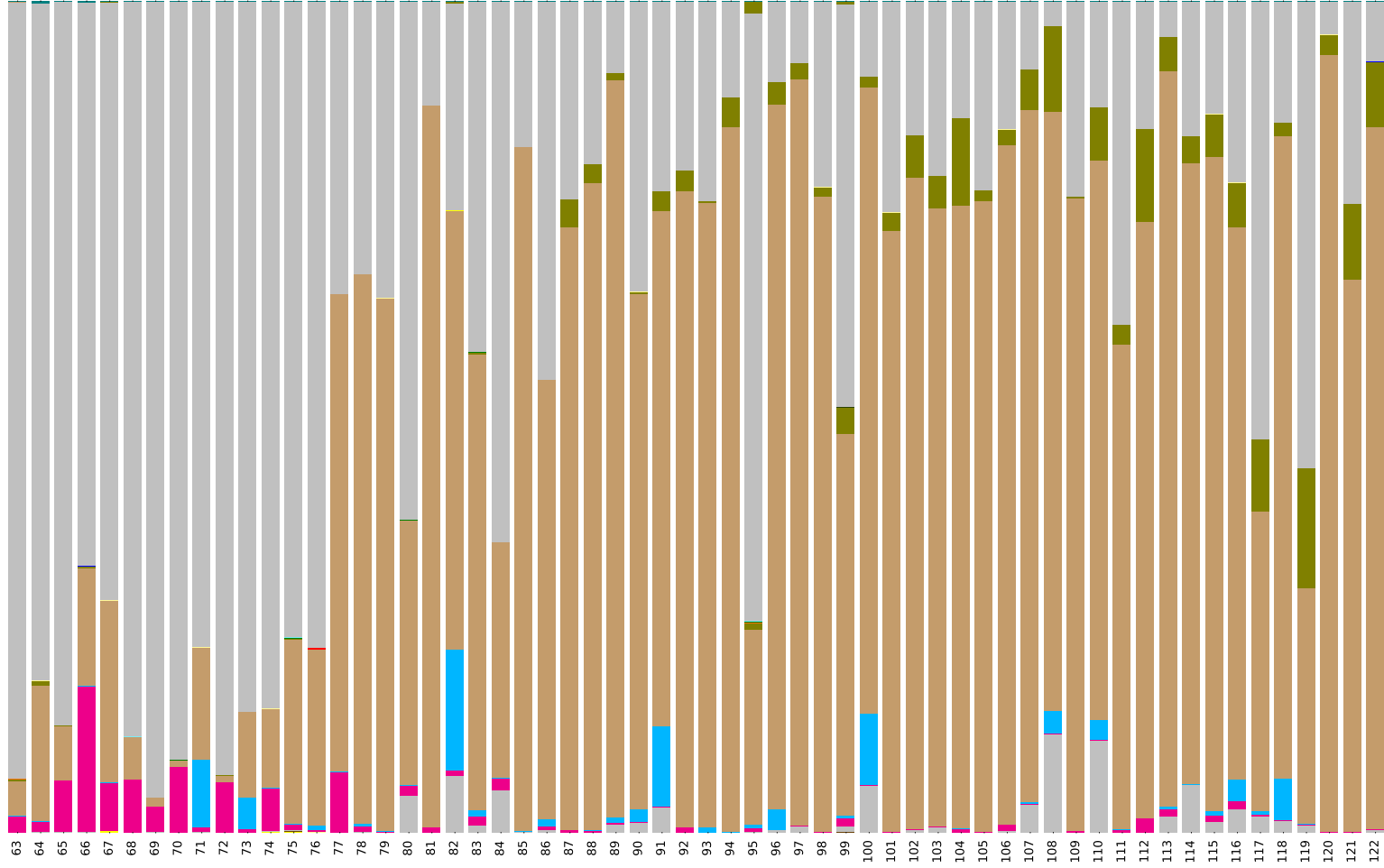

Supplement: Supplementary file 7 [file DataSheet2.ZIP › Supplemental_File_2_AviaryStudy2_TaxaSummaries/charts/sLYpsSUhtEcP68QSY4X1tikdTIZYDx.pdf]

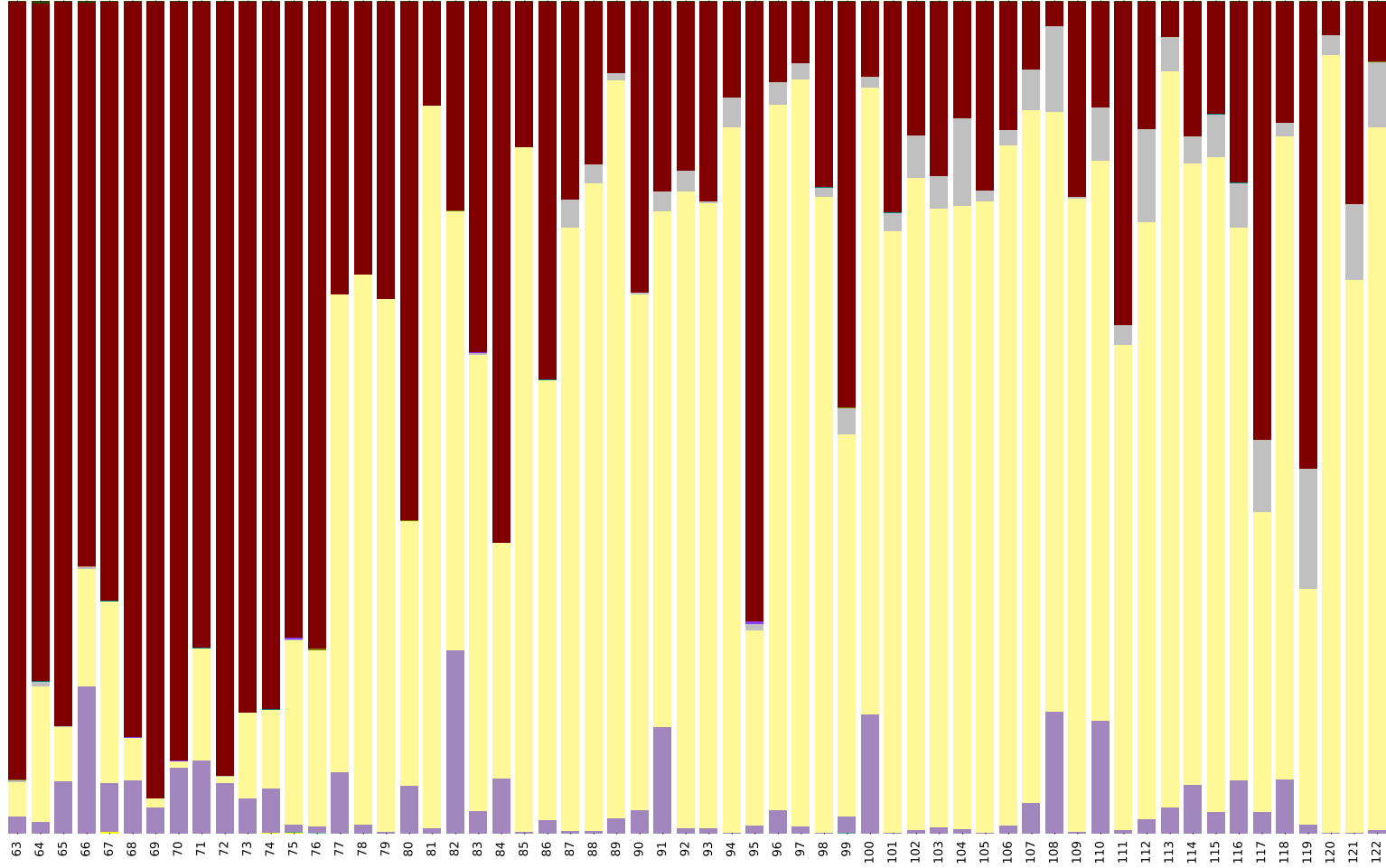

Supplement: Supplementary file 7 [file DataSheet2.ZIP › Supplemental_File_2_AviaryStudy2_TaxaSummaries/charts/t0J7UkihFZjsqQa2Y5Z16B9W3zDnHh.pdf]

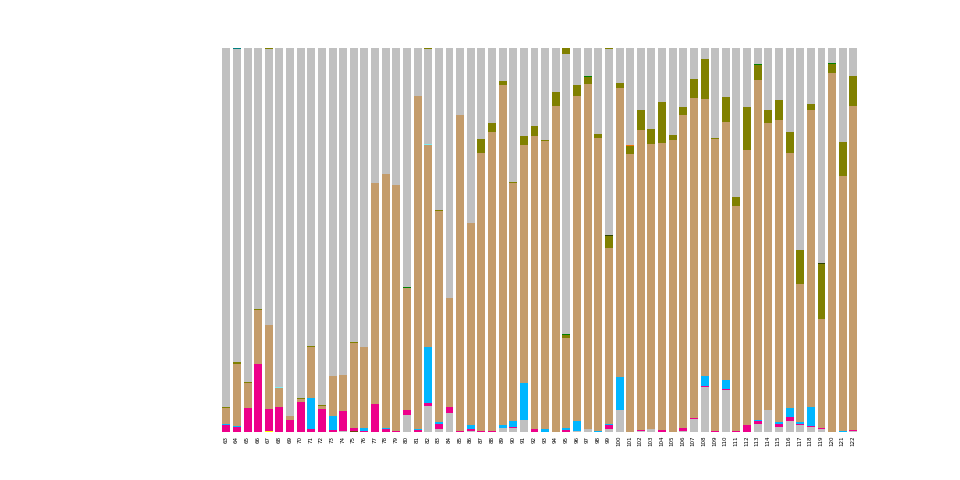

Supplement: Supplementary file 7 [file DataSheet2.ZIP › Supplemental_File_2_AviaryStudy2_TaxaSummaries/charts/taX472TNERZLGY5iD8JkD7KZbFNOpZ.png]

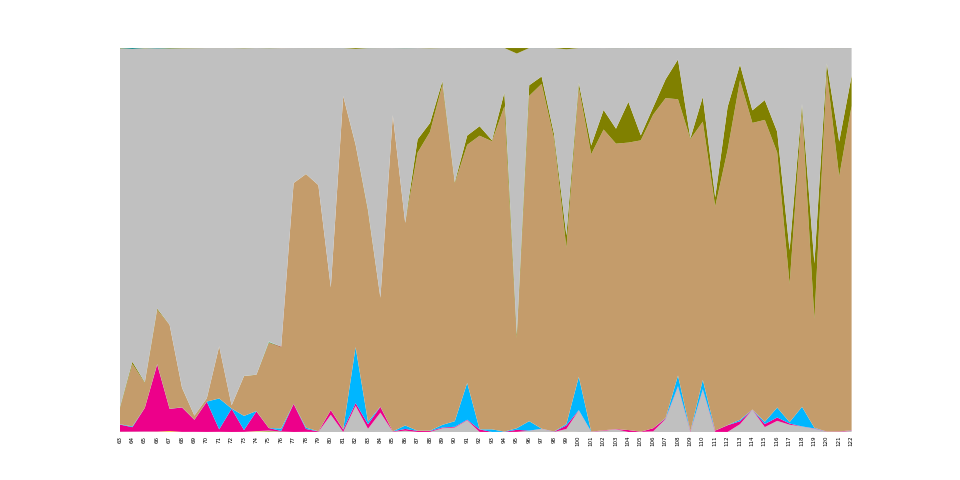

Supplement: Supplementary file 7 [file DataSheet2.ZIP › Supplemental_File_2_AviaryStudy2_TaxaSummaries/charts/u8iZCZYt9Jhe81z1L6d2xa3xoxoWxL.png]

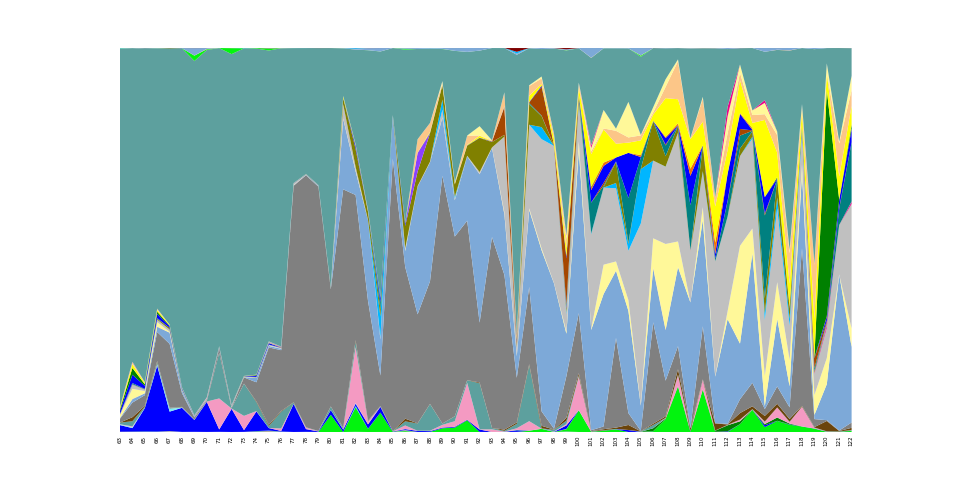

Supplement: Supplementary file 7 [file DataSheet2.ZIP › Supplemental_File_2_AviaryStudy2_TaxaSummaries/charts/yjz3xkFoZPEh0rHXWlZzGntRgpDpFA.png]

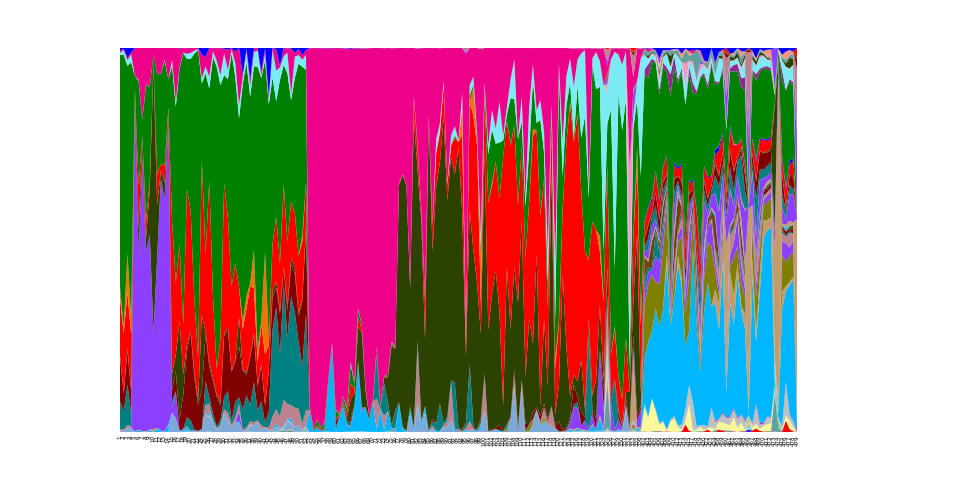

Supplement: Supplementary file 8 [file DataSheet3.ZIP › Supplemental_File_3_AviaryBarn_TaxaSummaries/charts/1oyW7TWKkmsqlFsEEwb65sf0bCzn7u.png]

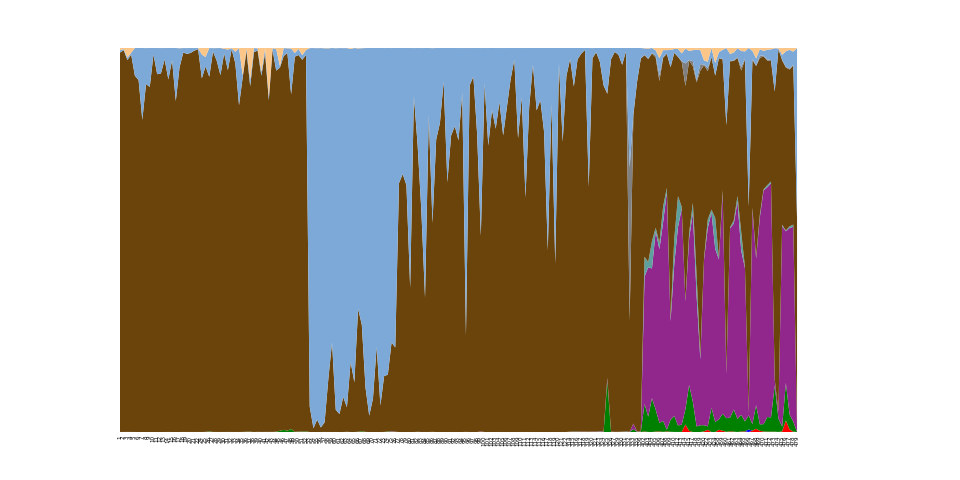

Supplement: Supplementary file 8 [file DataSheet3.ZIP › Supplemental_File_3_AviaryBarn_TaxaSummaries/charts/4FKzb00UWYsEegUSMqIg0i0qNNzrez.png]

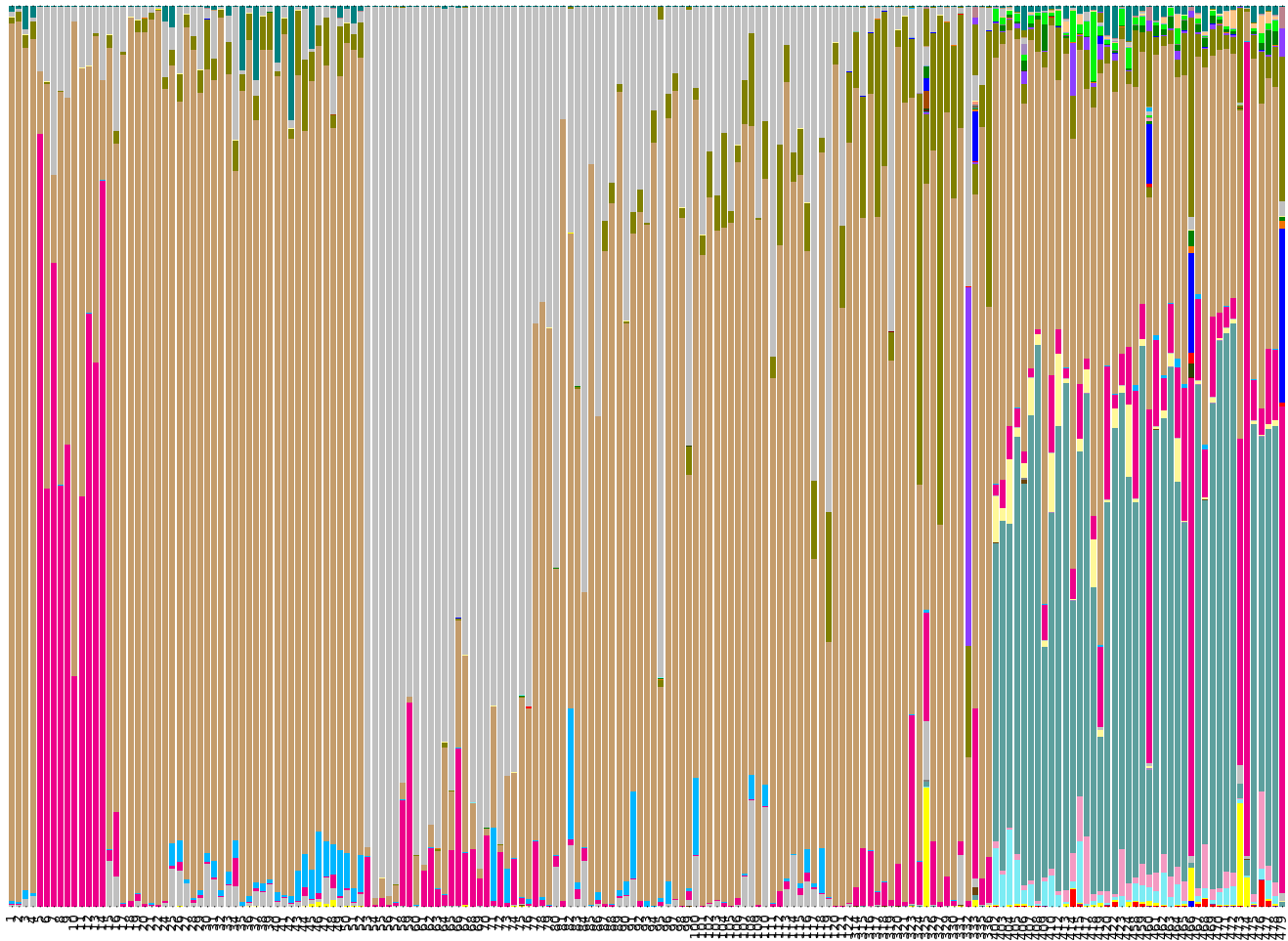

Supplement: Supplementary file 8 [file DataSheet3.ZIP › Supplemental_File_3_AviaryBarn_TaxaSummaries/charts/CeMotKihhsAs9MYy250U05b94MQ86W.pdf]

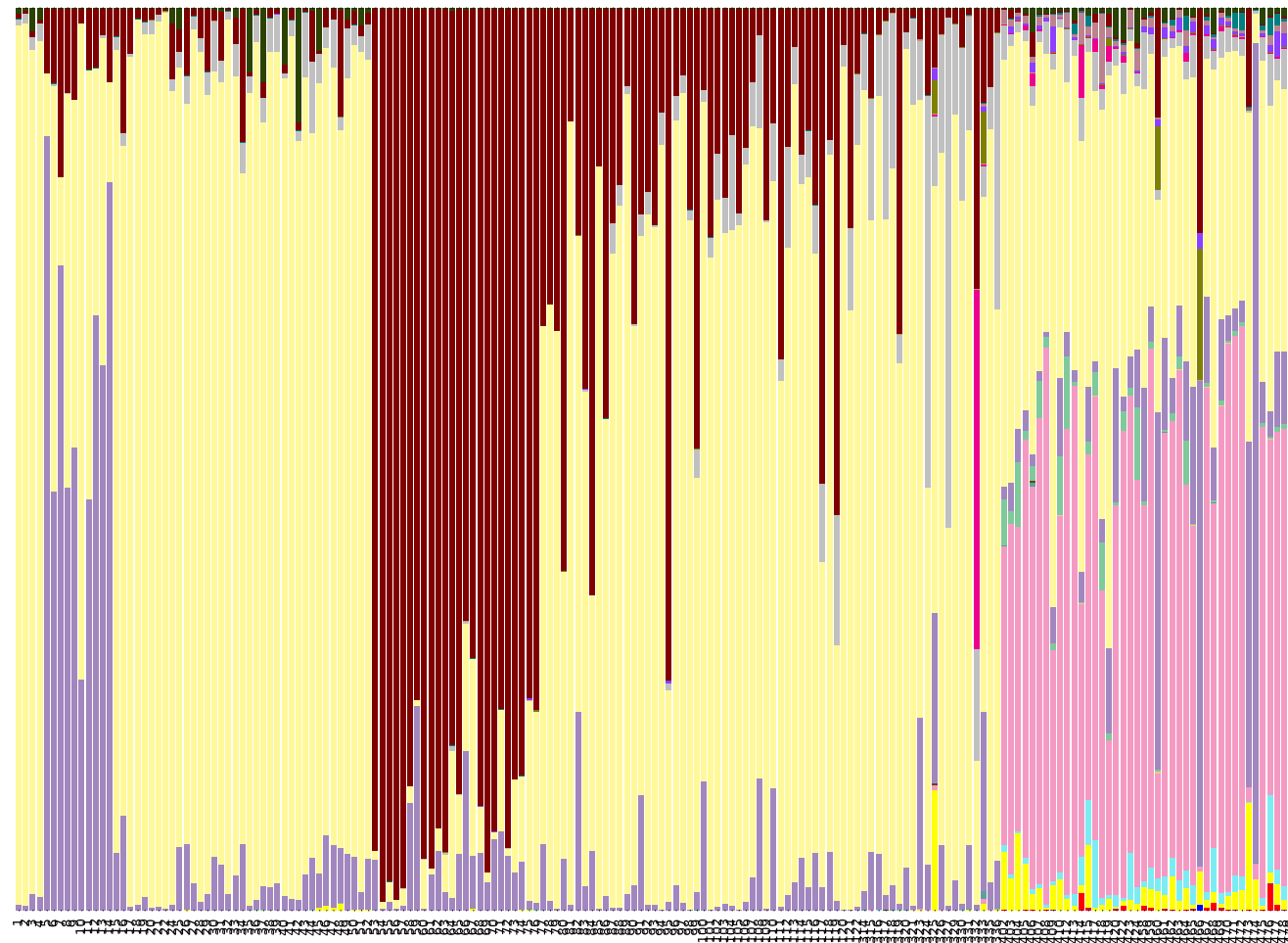

Supplement: Supplementary file 8 [file DataSheet3.ZIP › Supplemental_File_3_AviaryBarn_TaxaSummaries/charts/Dcd1znSMaQWOhIBRUbwP3bBdHH0Qmr.pdf]

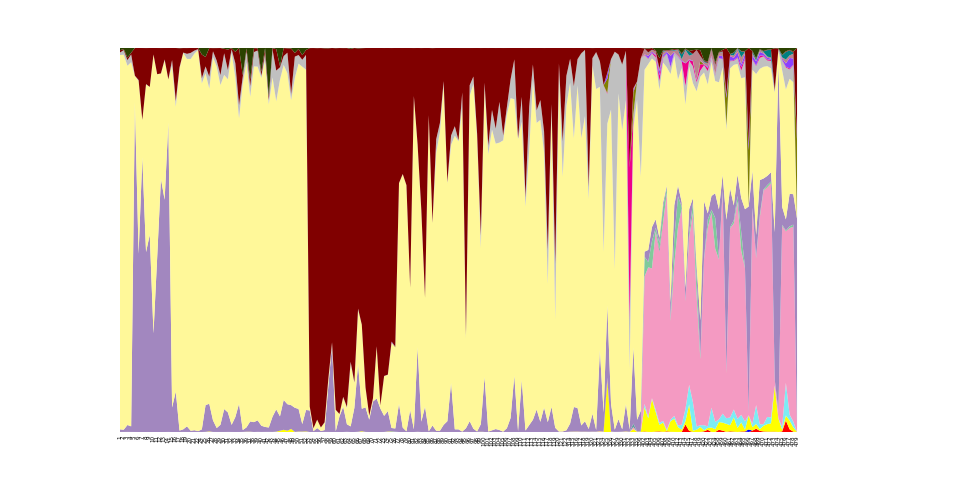

Supplement: Supplementary file 8 [file DataSheet3.ZIP › Supplemental_File_3_AviaryBarn_TaxaSummaries/charts/EHABeym4QunA8Zy2pkCIEzbqy3Z33z.png]

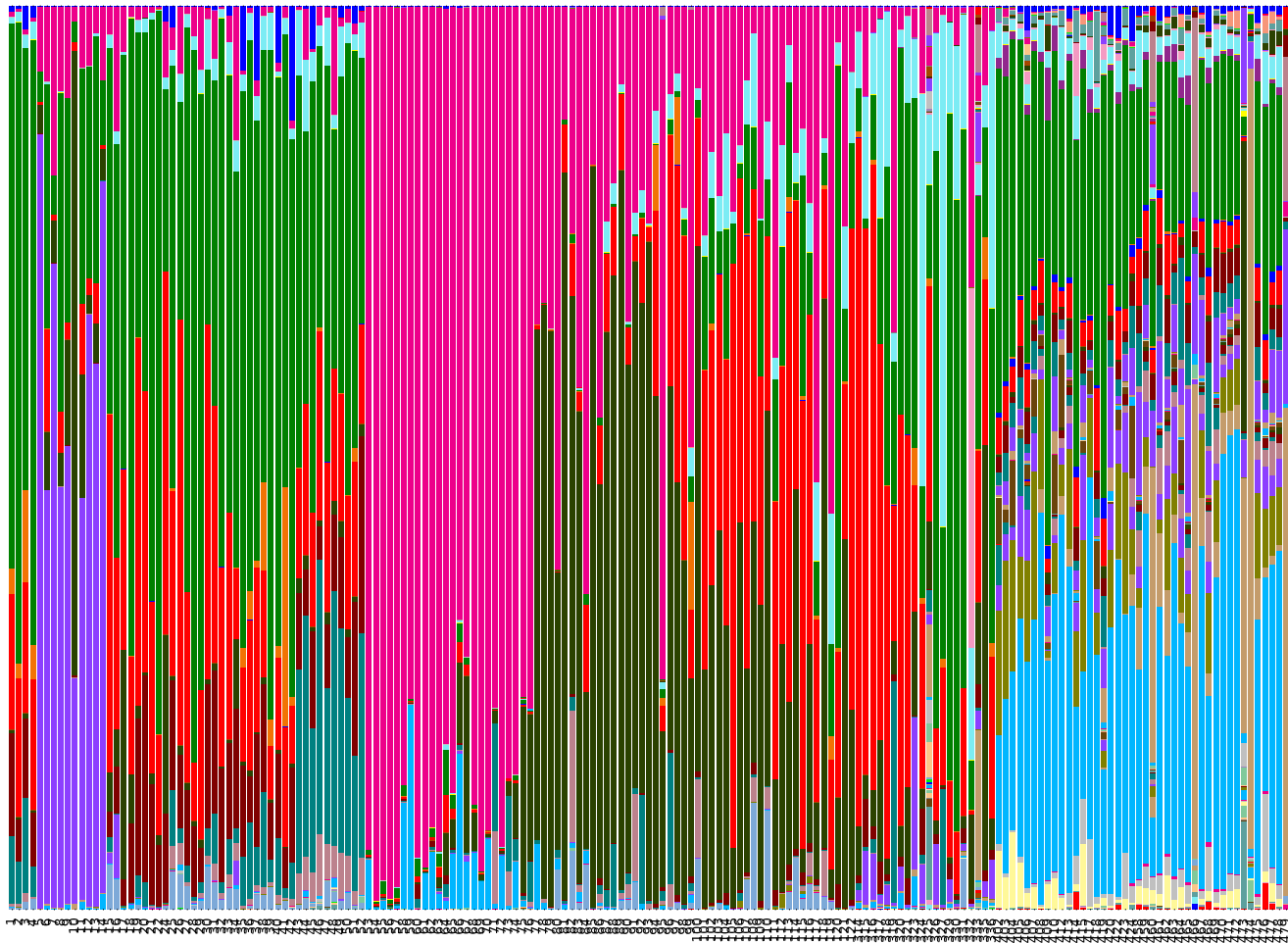

Supplement: Supplementary file 8 [file DataSheet3.ZIP › Supplemental_File_3_AviaryBarn_TaxaSummaries/charts/fb5f75JyyzzYd31oU05rrs2wDZlfPg.pdf]

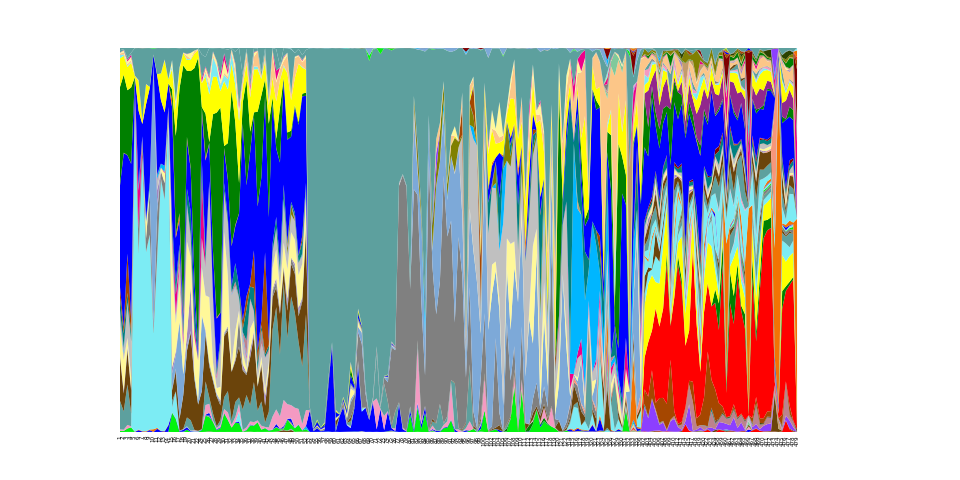

Supplement: Supplementary file 8 [file DataSheet3.ZIP › Supplemental_File_3_AviaryBarn_TaxaSummaries/charts/fKiZW5SGNhMGo1jPyha9tEZ38AGpIj.png]

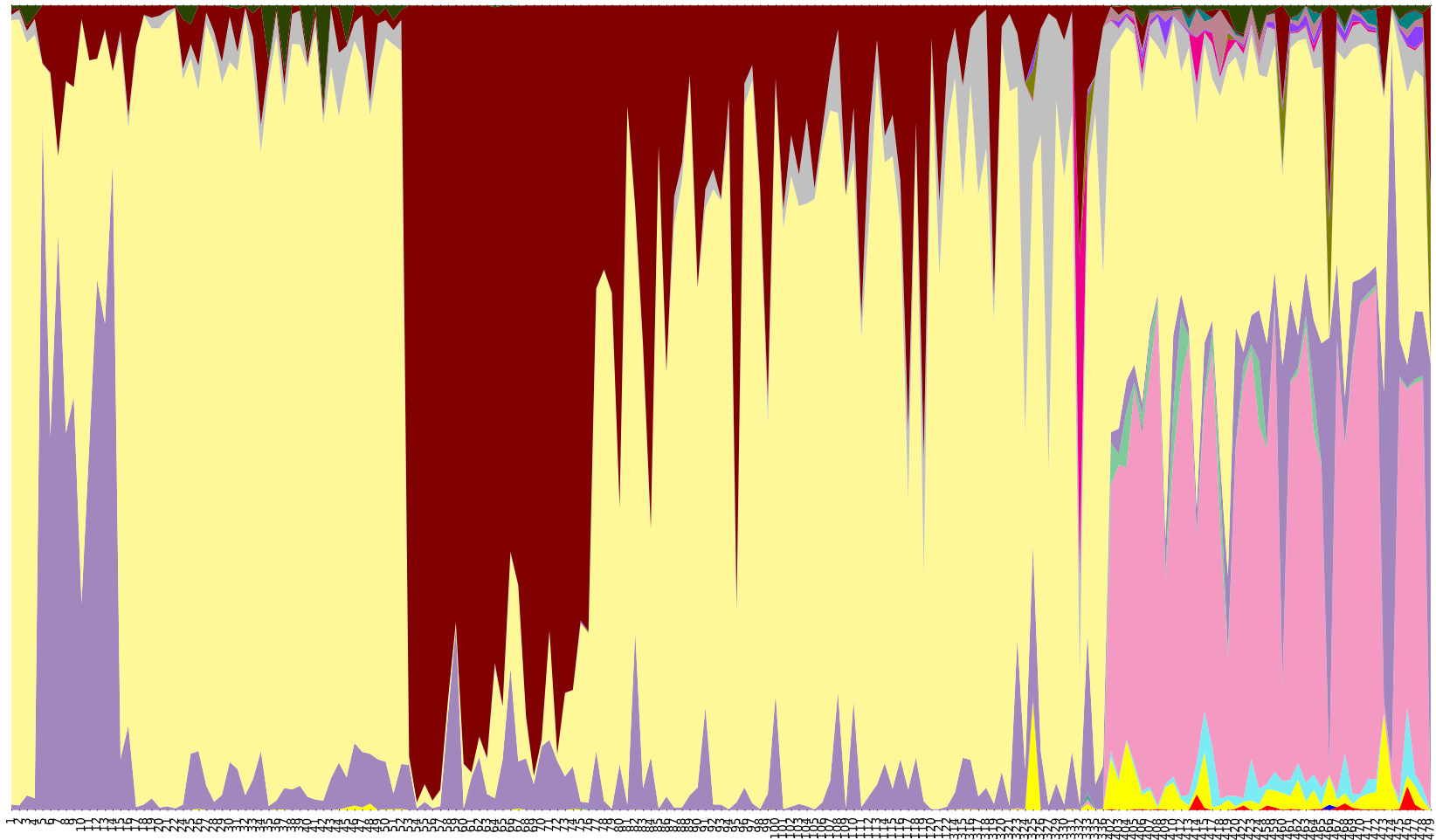

Supplement: Supplementary file 8 [file DataSheet3.ZIP › Supplemental_File_3_AviaryBarn_TaxaSummaries/charts/hlEuoBXSM2mkEZJZ5NhFJFHdbHNUO3.pdf]

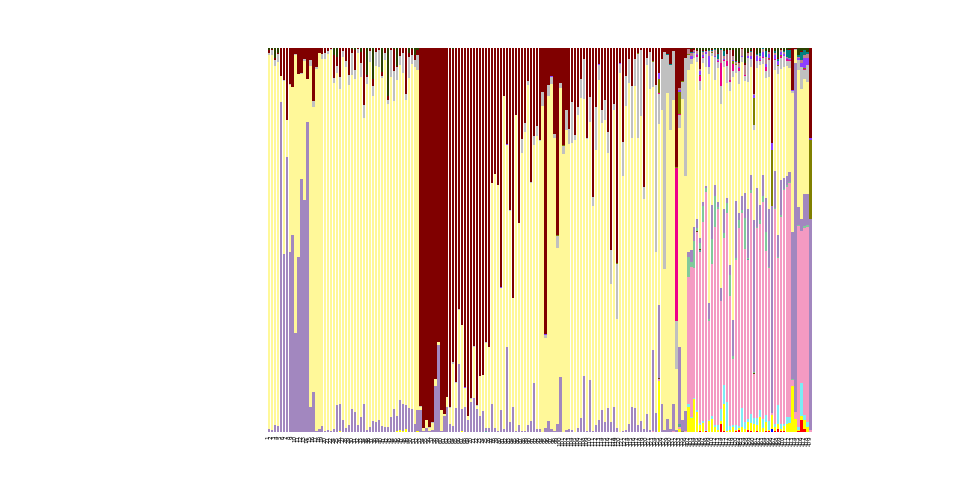

Supplement: Supplementary file 8 [file DataSheet3.ZIP › Supplemental_File_3_AviaryBarn_TaxaSummaries/charts/iJ7FCaeFZ0rBgg1T8SH09CBph0OXY6.png]

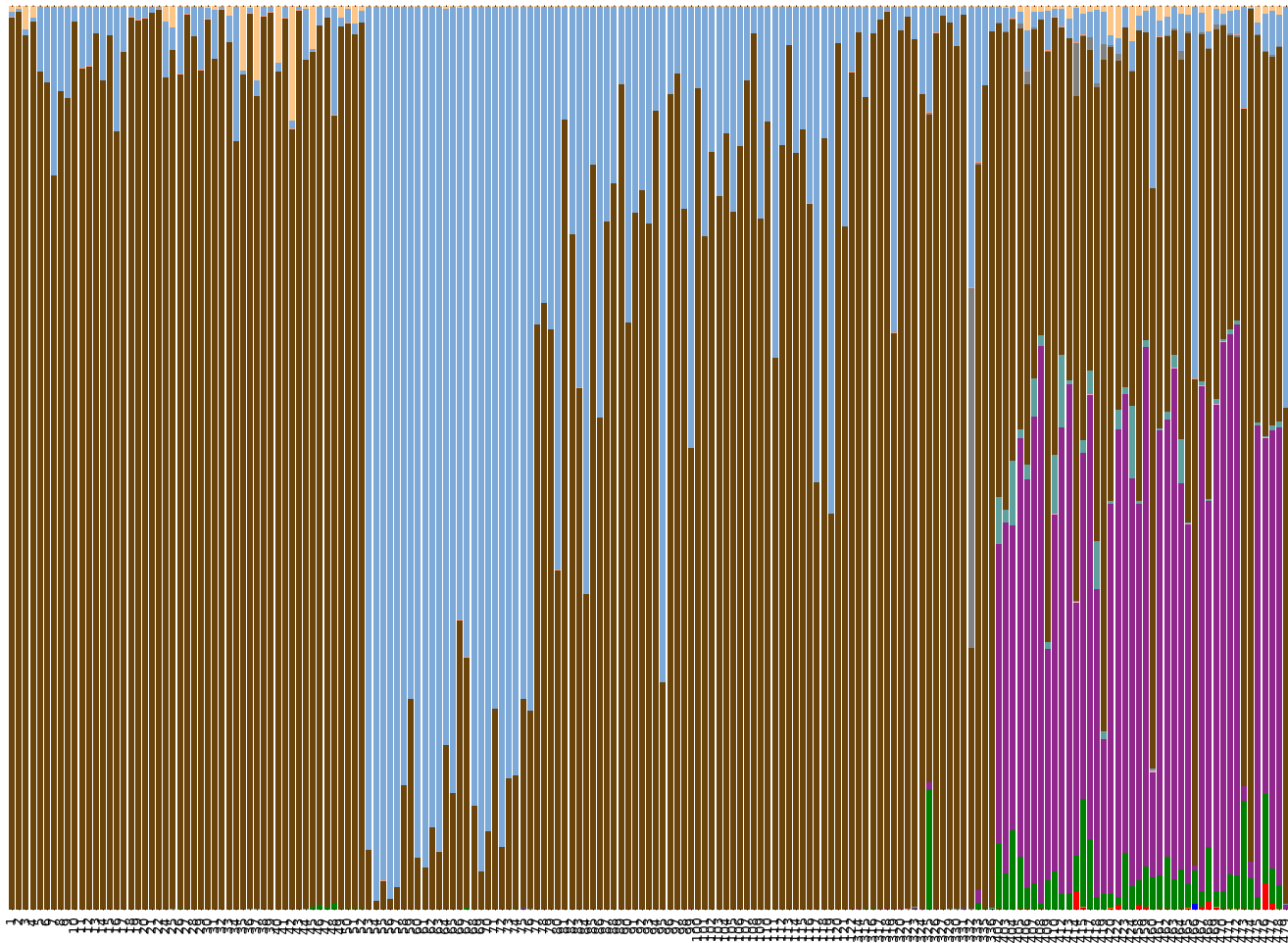

Supplement: Supplementary file 8 [file DataSheet3.ZIP › Supplemental_File_3_AviaryBarn_TaxaSummaries/charts/MNByZINQIdkA1xFZz6Pzz00jwRj0P3.pdf]

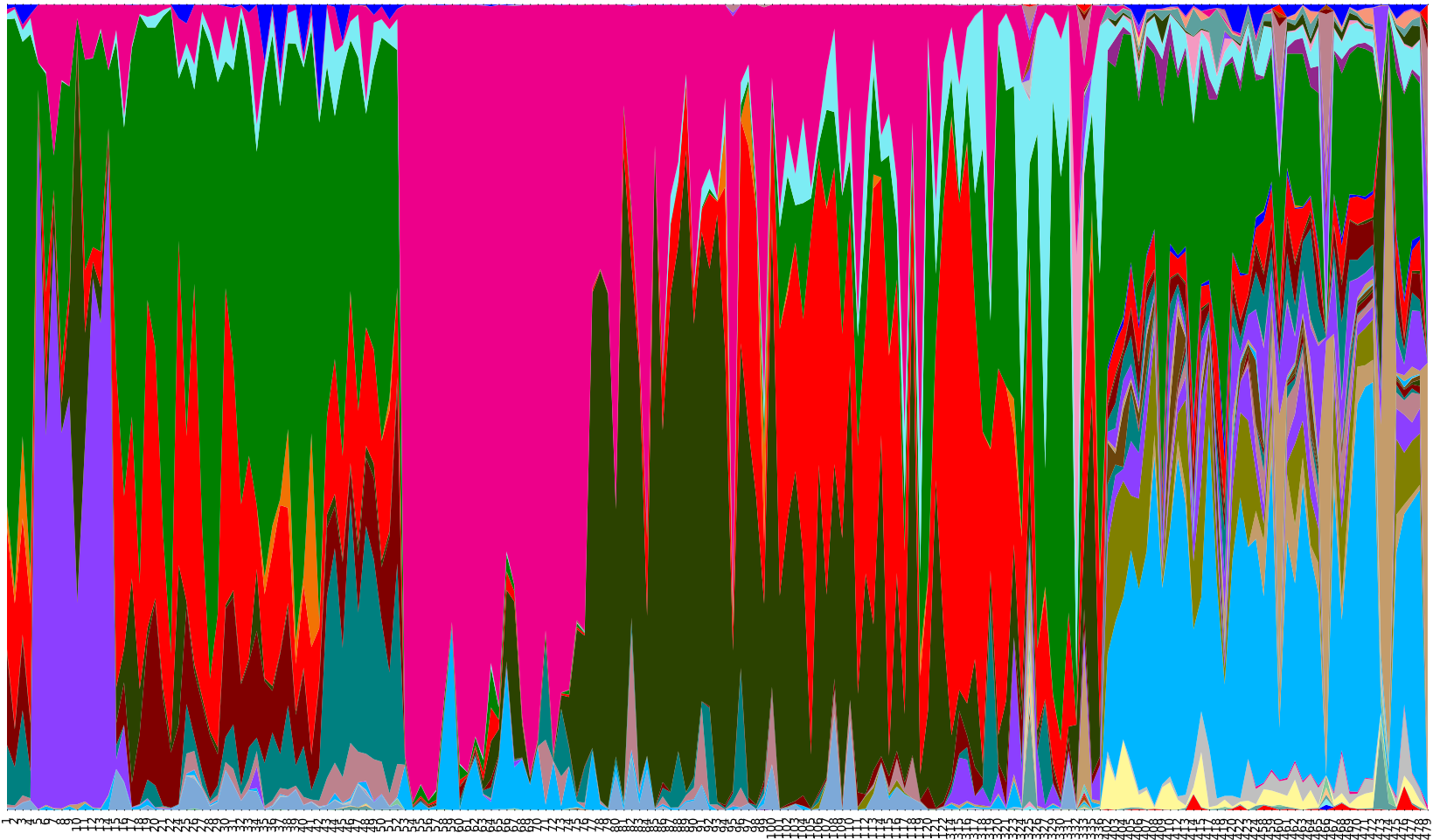

Supplement: Supplementary file 8 [file DataSheet3.ZIP › Supplemental_File_3_AviaryBarn_TaxaSummaries/charts/OELs9Id0dUbgLX09s155zp62OzFYEW.pdf]

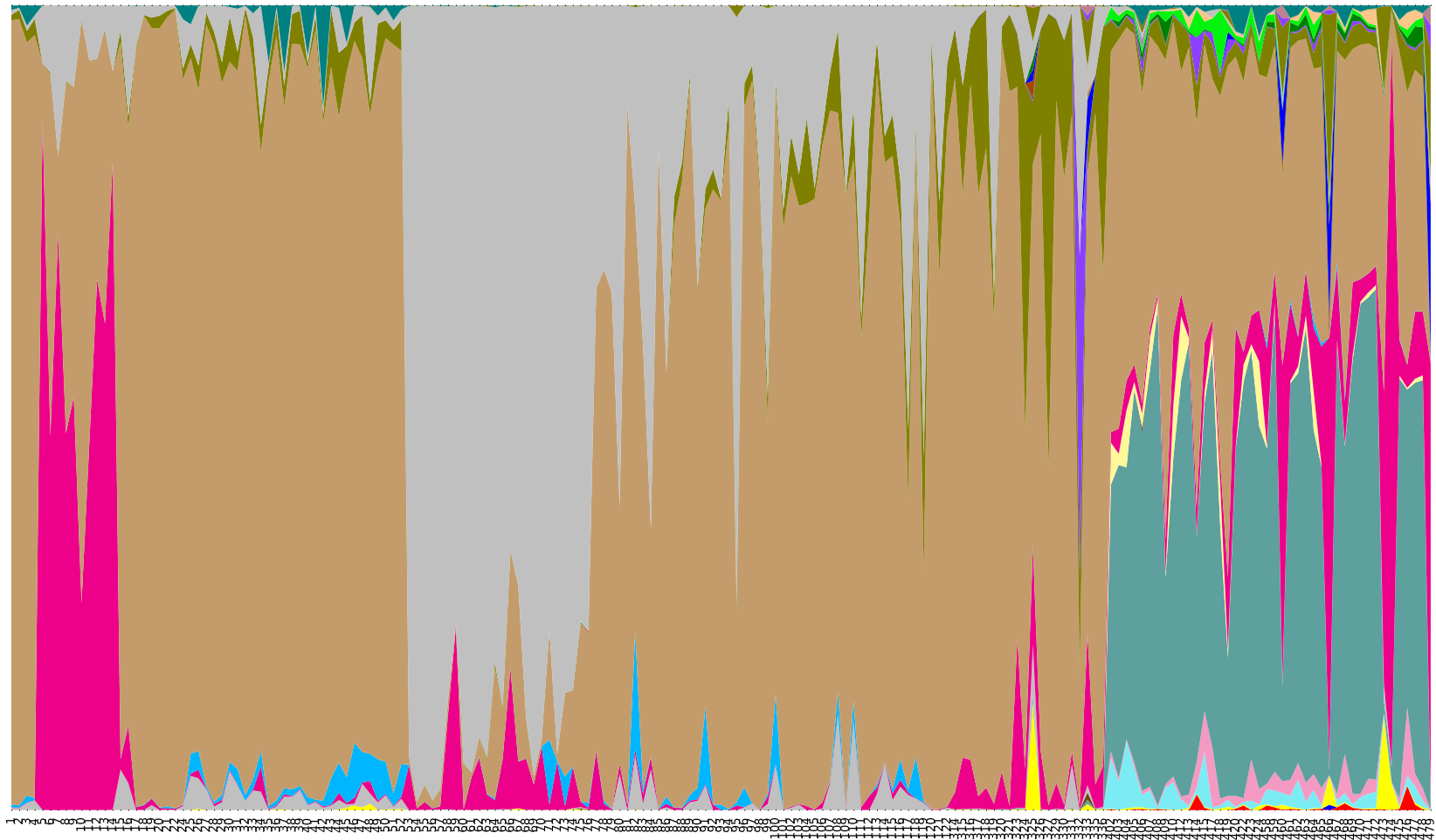

Supplement: Supplementary file 8 [file DataSheet3.ZIP › Supplemental_File_3_AviaryBarn_TaxaSummaries/charts/PQZXa5JMijtIie0kMdgqTHZ8Xh1y0g.pdf]

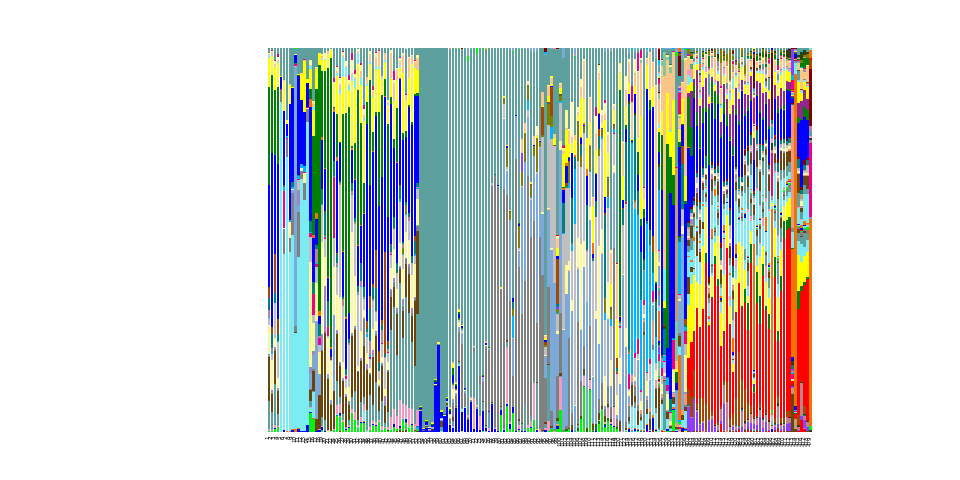

Supplement: Supplementary file 8 [file DataSheet3.ZIP › Supplemental_File_3_AviaryBarn_TaxaSummaries/charts/qPbzMR1hZtSYwMgamnsP5oSfXKycaX.png]

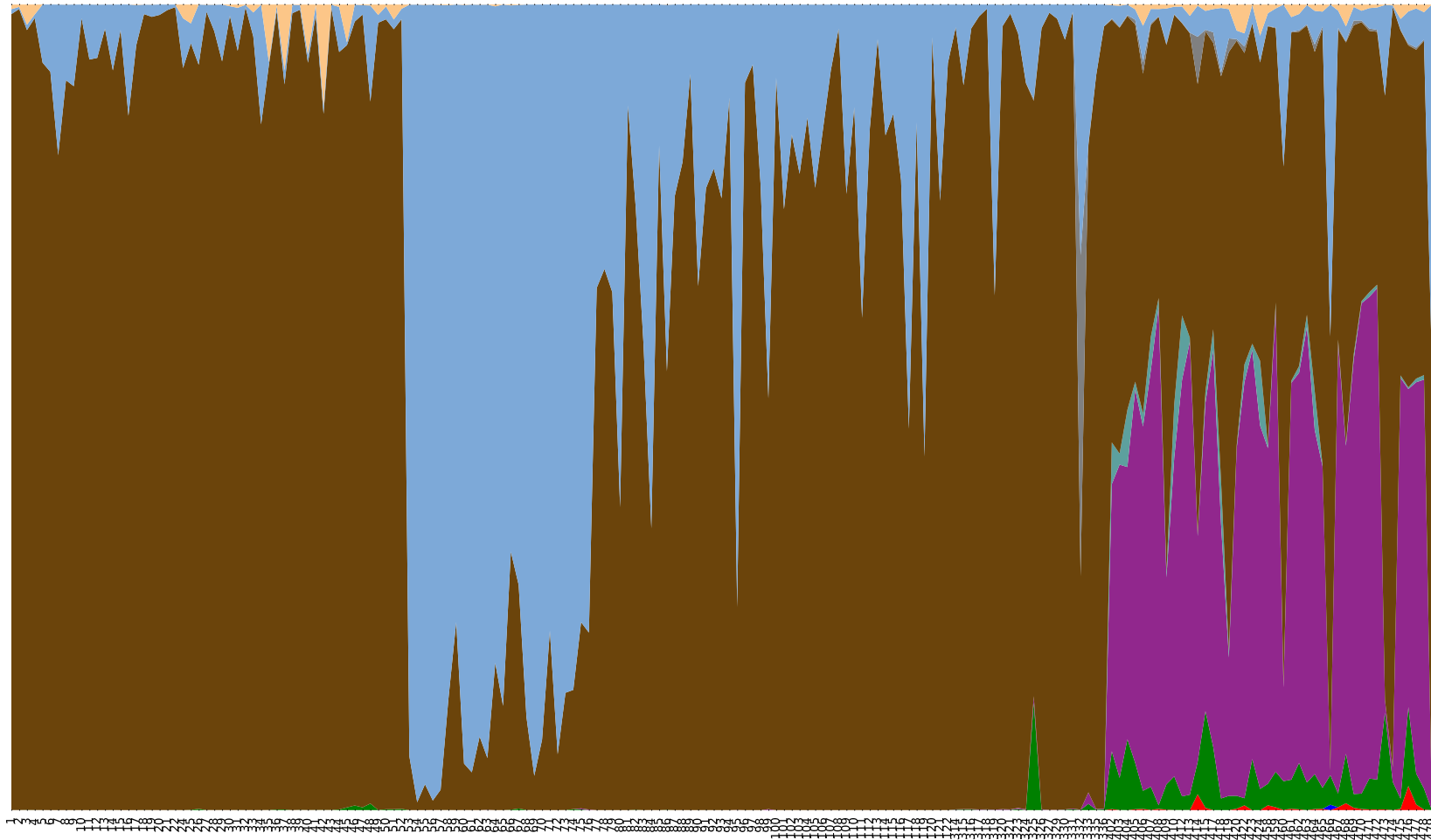

Supplement: Supplementary file 8 [file DataSheet3.ZIP › Supplemental_File_3_AviaryBarn_TaxaSummaries/charts/QRNcZ4Bzzpp2RMgXJCkzHZTUhNlKHo.pdf]

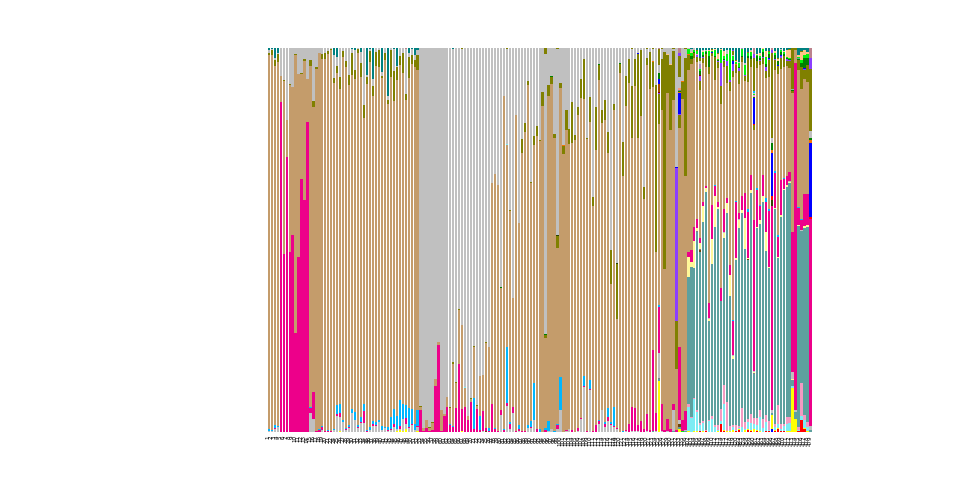

Supplement: Supplementary file 8 [file DataSheet3.ZIP › Supplemental_File_3_AviaryBarn_TaxaSummaries/charts/SBU0JlzeWfKOluZTiIsUR8lskUi9k0.png]

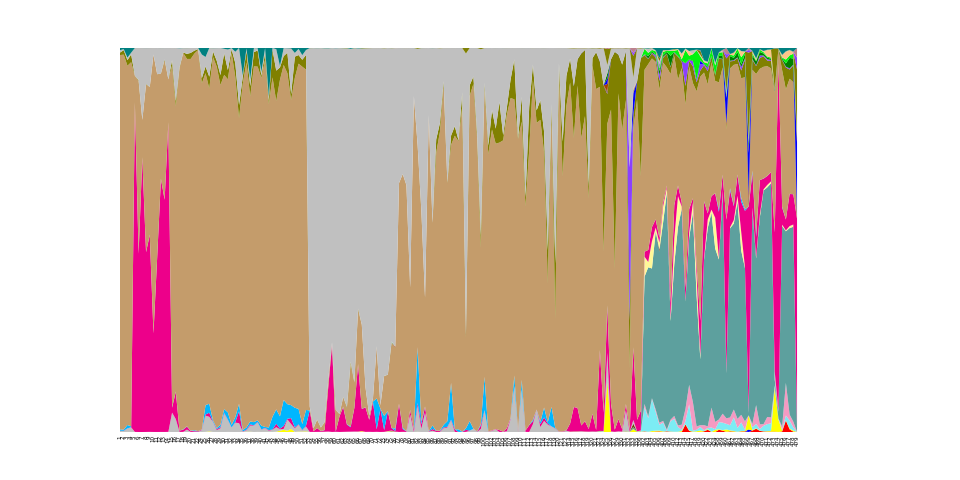

Supplement: Supplementary file 8 [file DataSheet3.ZIP › Supplemental_File_3_AviaryBarn_TaxaSummaries/charts/Tf50QWZUS3xuRd75oNZbAgW9eC308z.png]

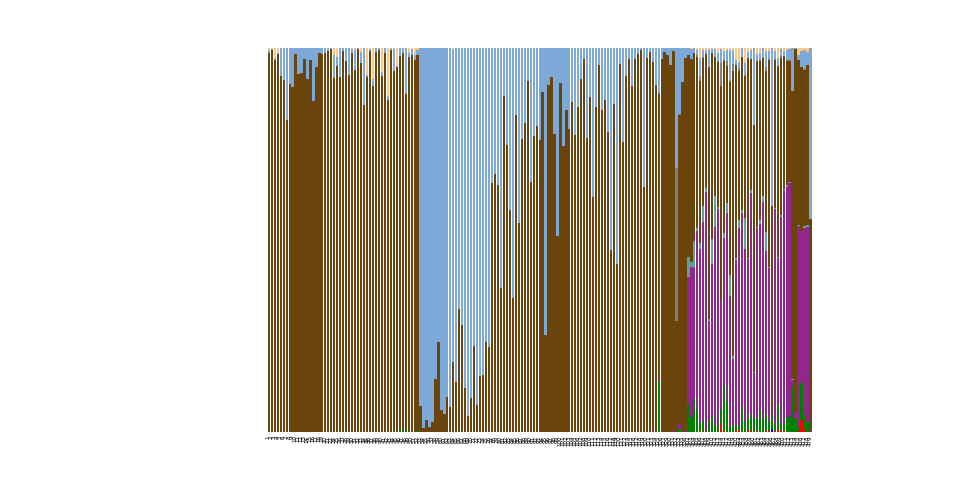

Supplement: Supplementary file 8 [file DataSheet3.ZIP › Supplemental_File_3_AviaryBarn_TaxaSummaries/charts/wW7jDQoT2c6bLld1WF0oMpd06ACAjB.png]

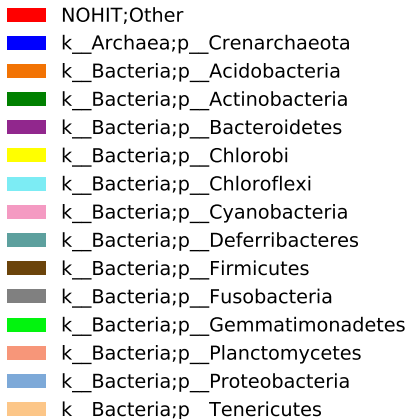

Supplement: Supplementary file 8 [file DataSheet3.ZIP › Supplemental_File_3_AviaryBarn_TaxaSummaries/charts/wW7jDQoT2c6bLld1WF0oMpd06ACAjB_legend.pdf]

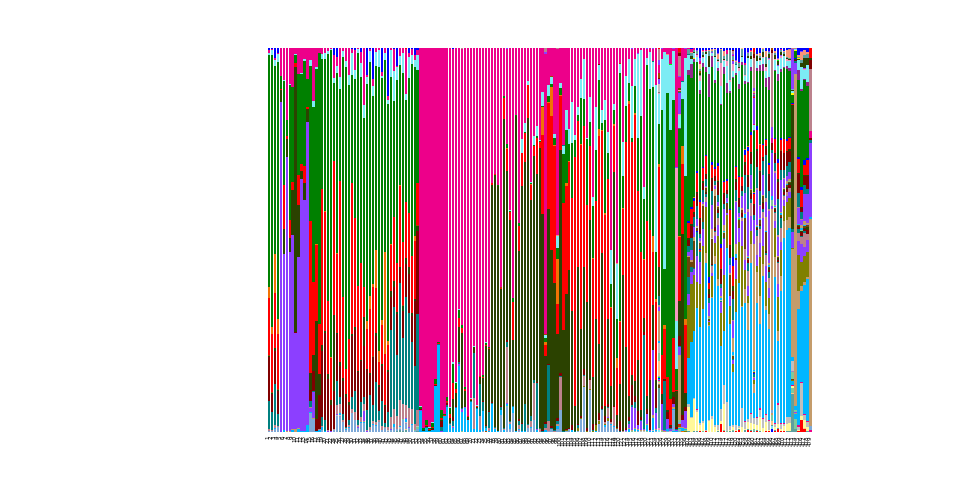

Supplement: Supplementary file 8 [file DataSheet3.ZIP › Supplemental_File_3_AviaryBarn_TaxaSummaries/charts/Y4kzSuDGzwTzDfnXBluRfNDGKQzslf.png]

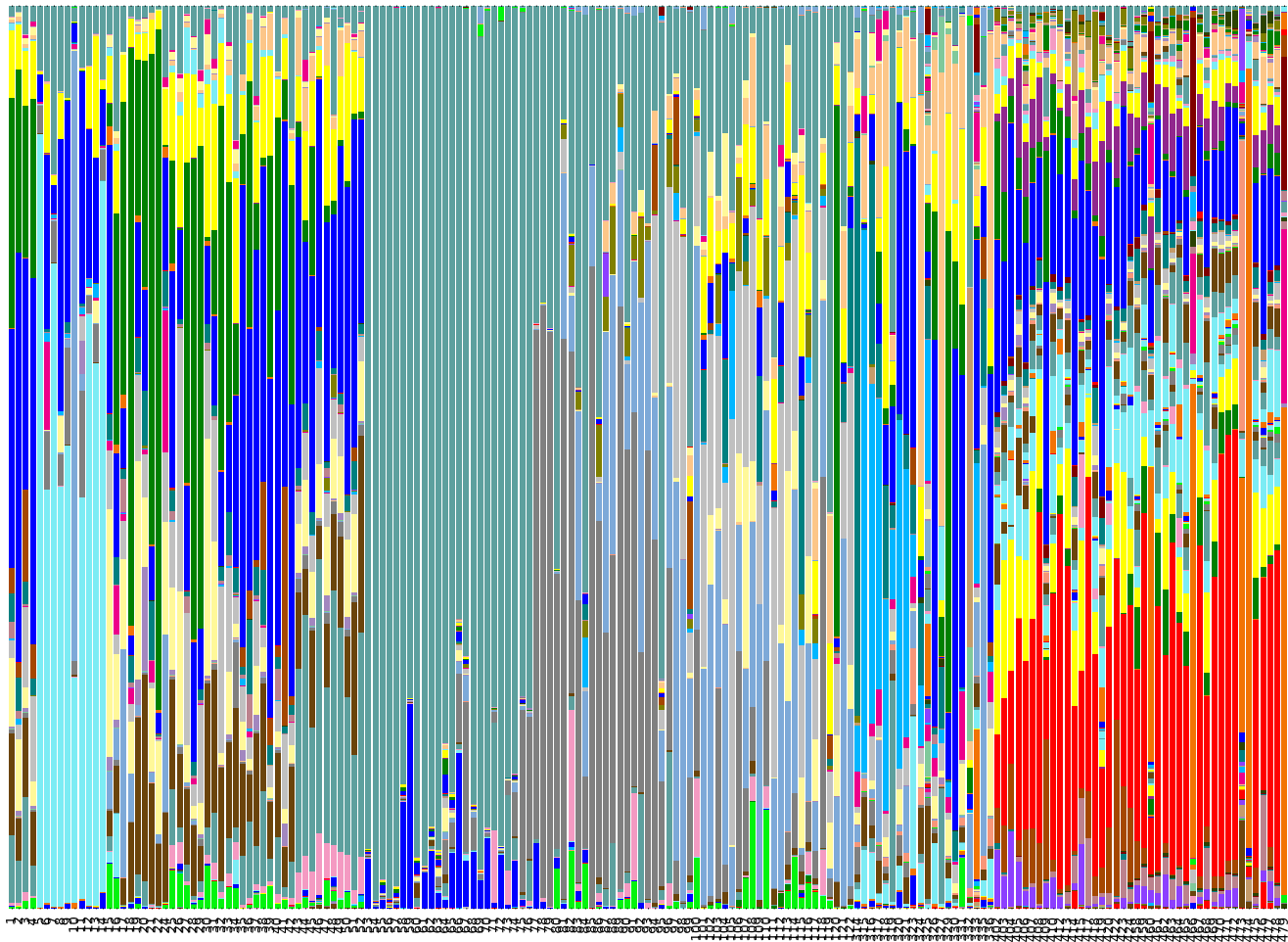

Supplement: Supplementary file 8 [file DataSheet3.ZIP › Supplemental_File_3_AviaryBarn_TaxaSummaries/charts/zD1sK1nFG2mY0OajJIJ0ToiwFOtwii.pdf]

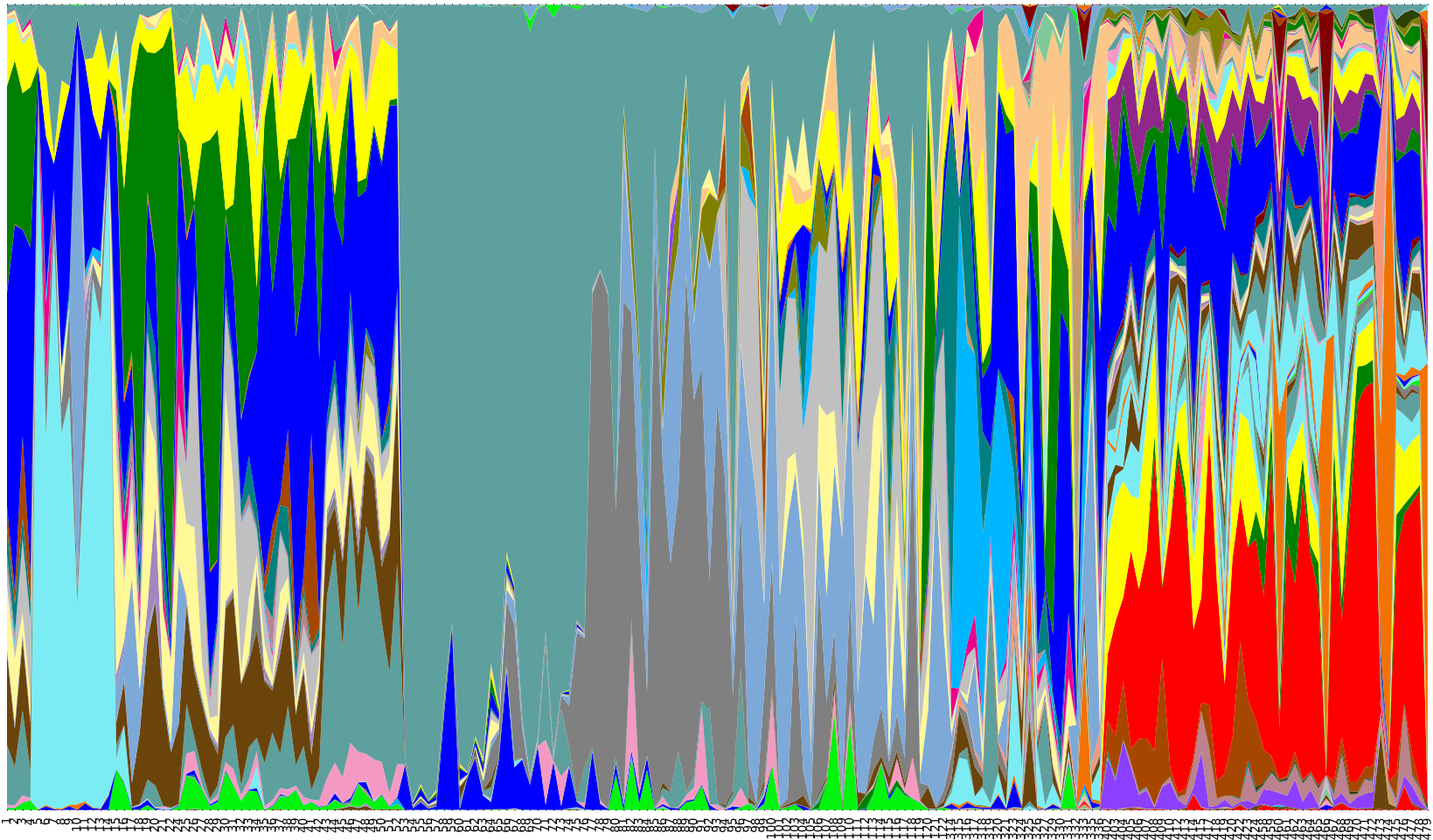

Supplement: Supplementary file 8 [file DataSheet3.ZIP › Supplemental_File_3_AviaryBarn_TaxaSummaries/charts/ZHeiB4GwnABue3kzFpgMbxH0lUrkc4.pdf]

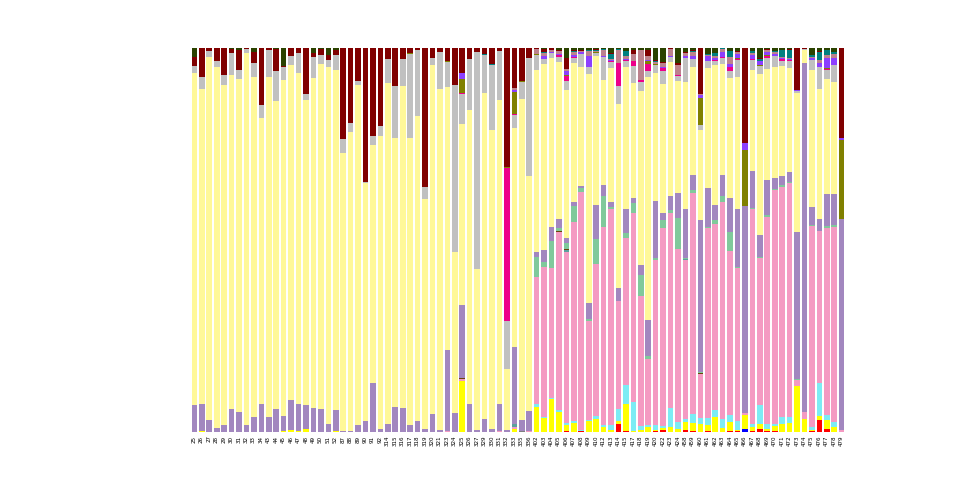

Supplement: Supplementary file 9 [file DataSheet4.ZIP › Supplemental_File_4_AviaryBarn_EnviroAgeGrp_TaxaSummaries/charts/0NCHndIUmLr0oGtsDxOrKwu0SeP1th.png]

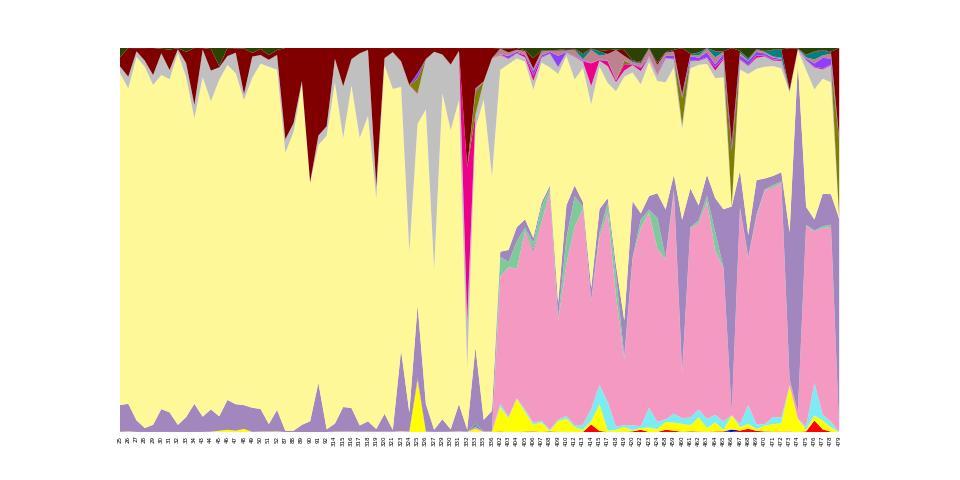

Supplement: Supplementary file 9 [file DataSheet4.ZIP › Supplemental_File_4_AviaryBarn_EnviroAgeGrp_TaxaSummaries/charts/2JsMRCmTmUdwylz1x0lFL1OjkWt2zD.png]

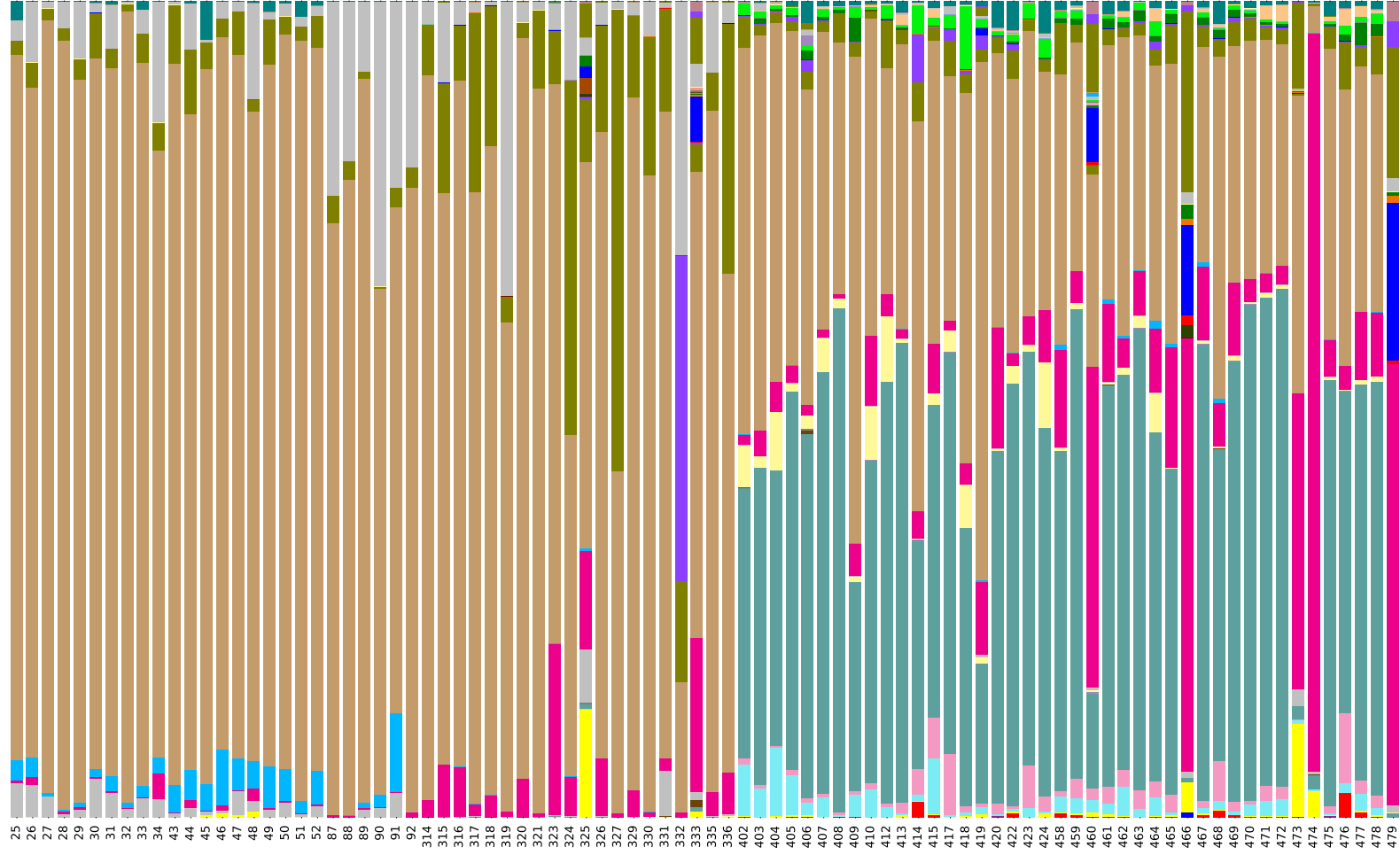

Supplement: Supplementary file 9 [file DataSheet4.ZIP › Supplemental_File_4_AviaryBarn_EnviroAgeGrp_TaxaSummaries/charts/Cc7cBpx9hhX81LOCdblinnnJ488w6X.pdf]

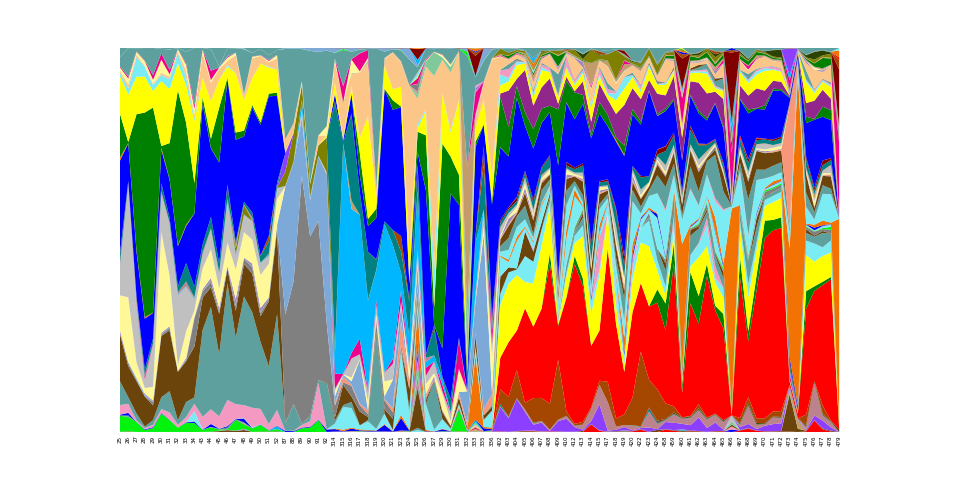

Supplement: Supplementary file 9 [file DataSheet4.ZIP › Supplemental_File_4_AviaryBarn_EnviroAgeGrp_TaxaSummaries/charts/Cdk8rQ0HjQBZCoxenii5NNNDeKTP3s.png]

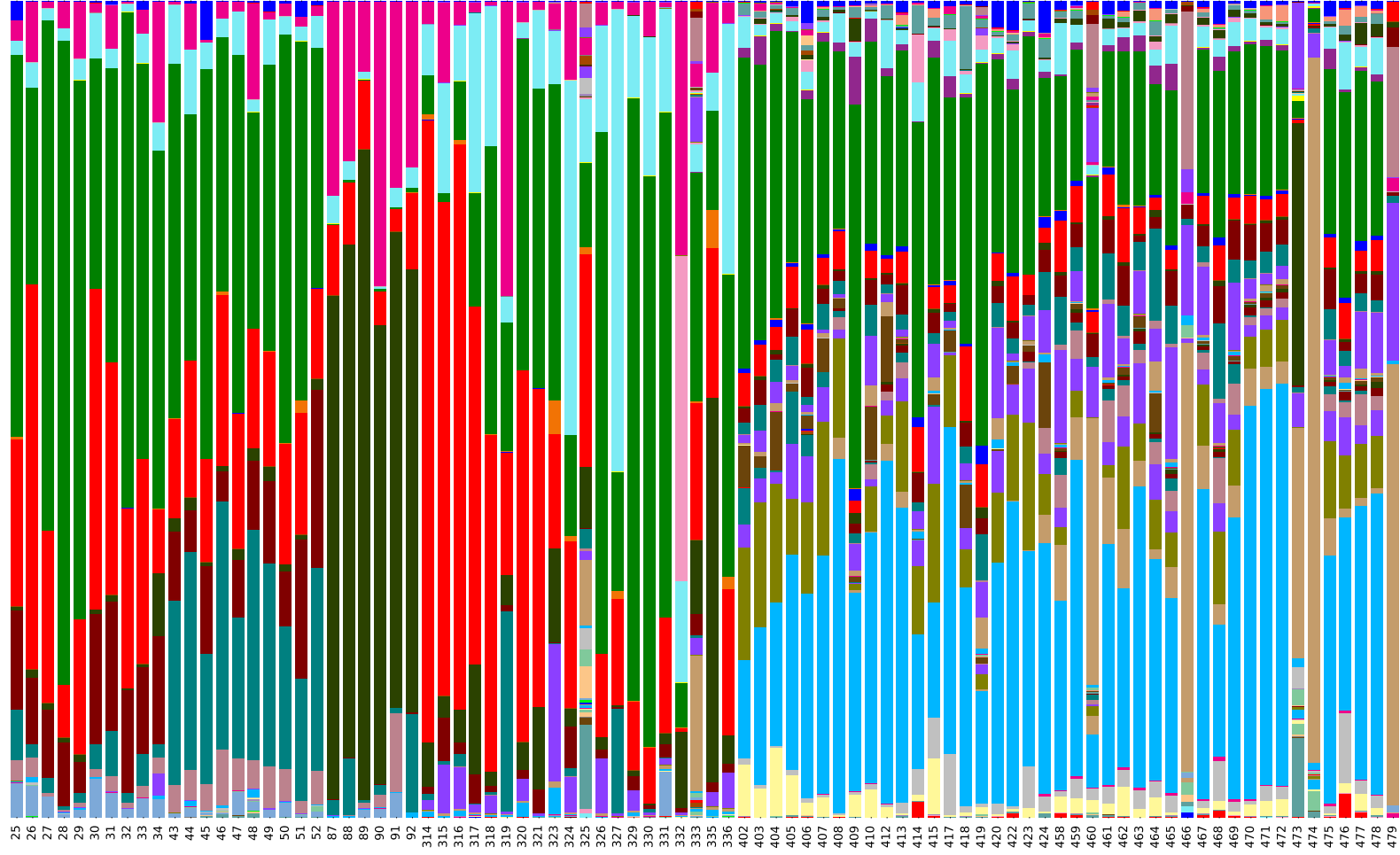

Supplement: Supplementary file 9 [file DataSheet4.ZIP › Supplemental_File_4_AviaryBarn_EnviroAgeGrp_TaxaSummaries/charts/dN6fdkmADgMd2jgGs53ynloo5pWQFd.pdf]

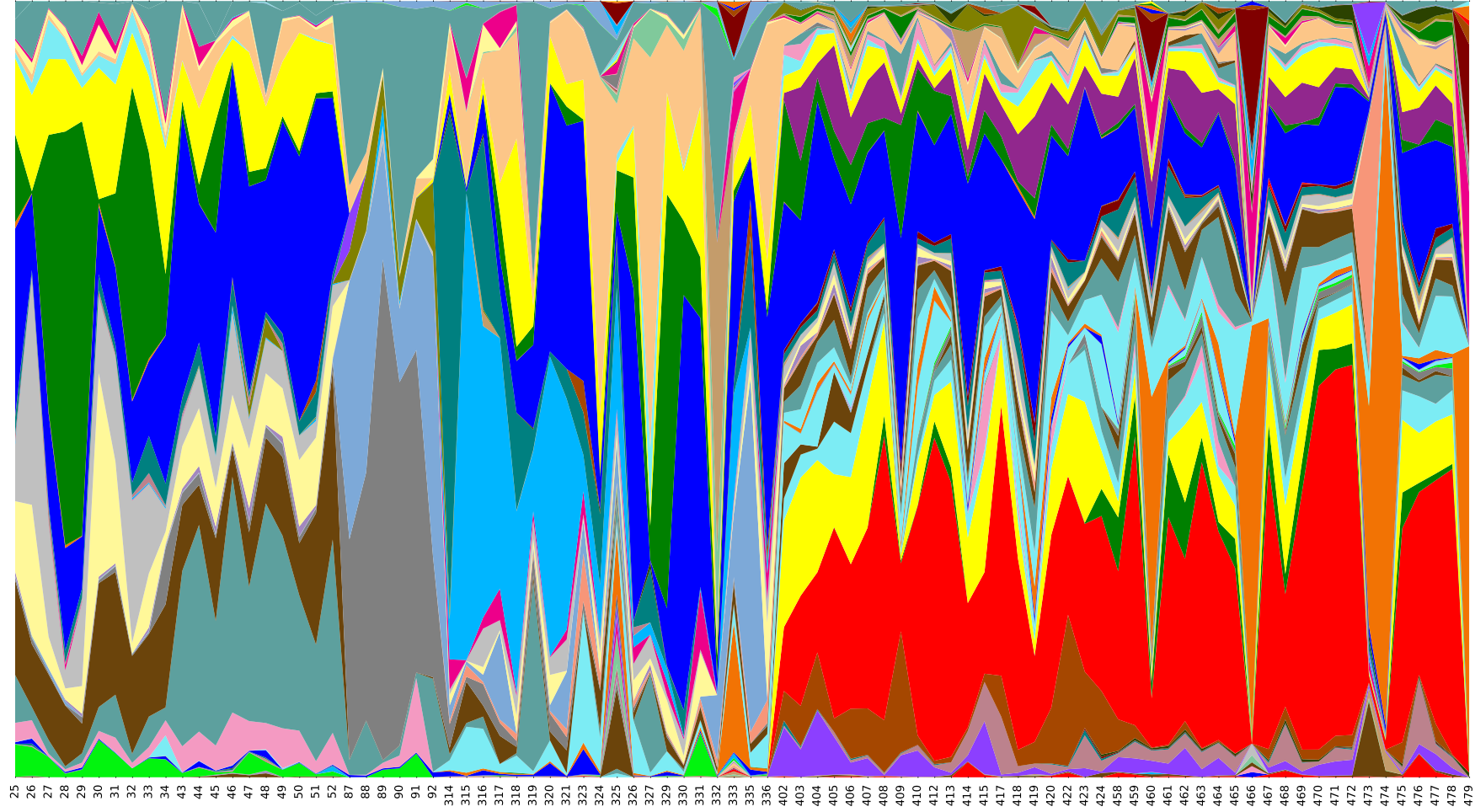

Supplement: Supplementary file 9 [file DataSheet4.ZIP › Supplemental_File_4_AviaryBarn_EnviroAgeGrp_TaxaSummaries/charts/HAPZxPw6H95Dm0ggn7Mzwd11jzo0id.pdf]

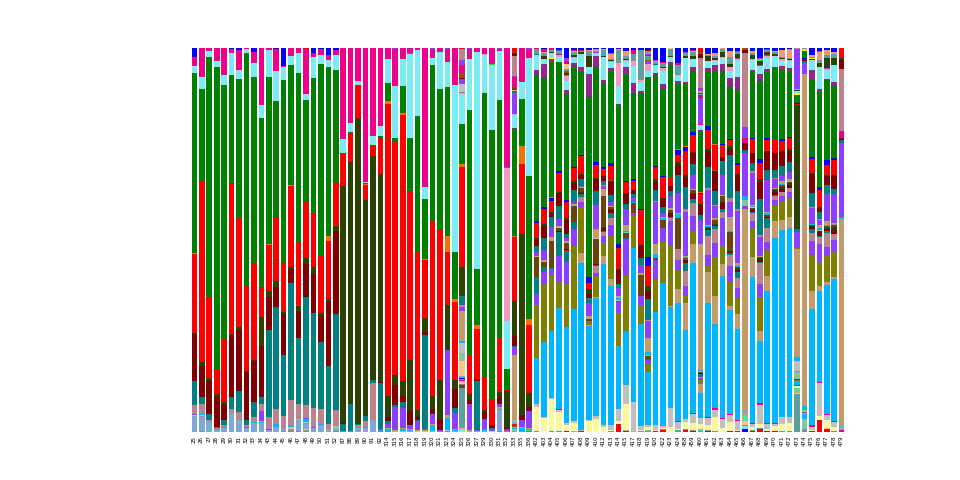

Supplement: Supplementary file 9 [file DataSheet4.ZIP › Supplemental_File_4_AviaryBarn_EnviroAgeGrp_TaxaSummaries/charts/hHaqX9DLDOFJ5LdCW7DWGXS5G89n5z.png]

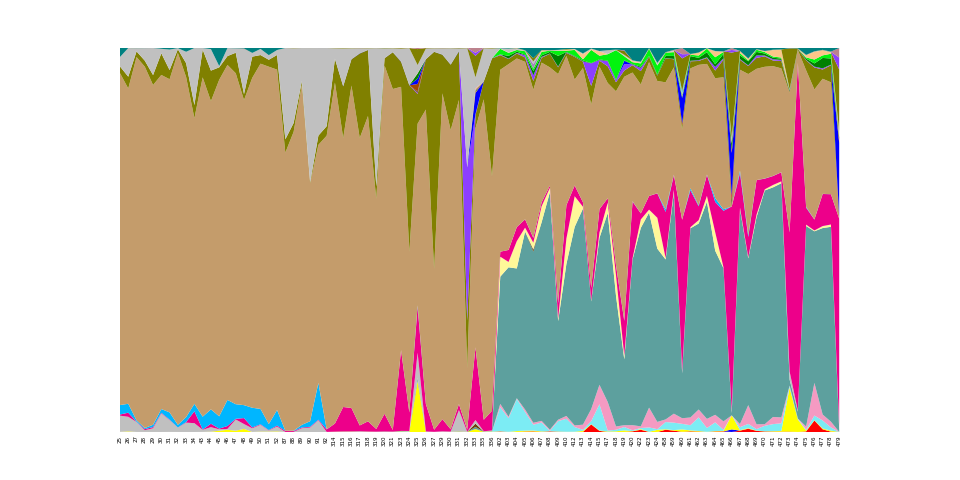

Supplement: Supplementary file 9 [file DataSheet4.ZIP › Supplemental_File_4_AviaryBarn_EnviroAgeGrp_TaxaSummaries/charts/iIczjm0JrJjNgEzRrnrRWPghzzxgpn.png]

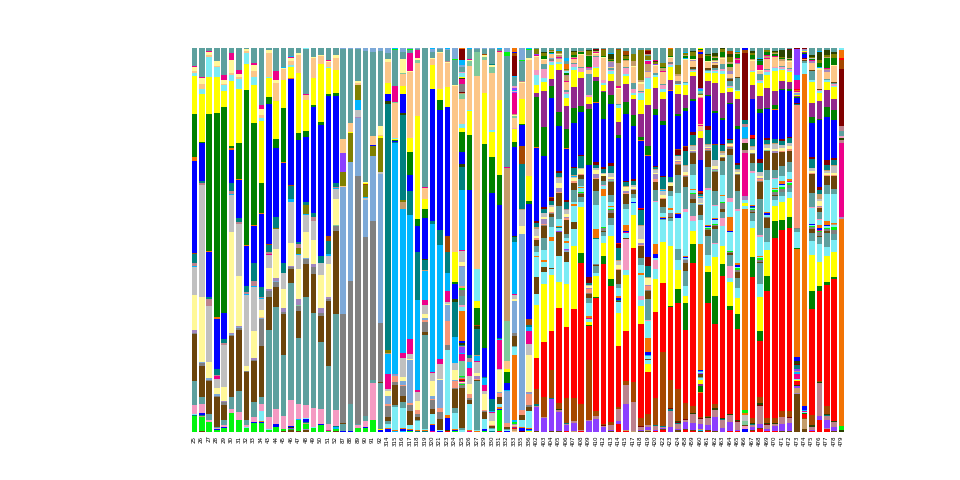

Supplement: Supplementary file 9 [file DataSheet4.ZIP › Supplemental_File_4_AviaryBarn_EnviroAgeGrp_TaxaSummaries/charts/ijApQ8HWowurzxpTLSForGuhJtchJc.png]

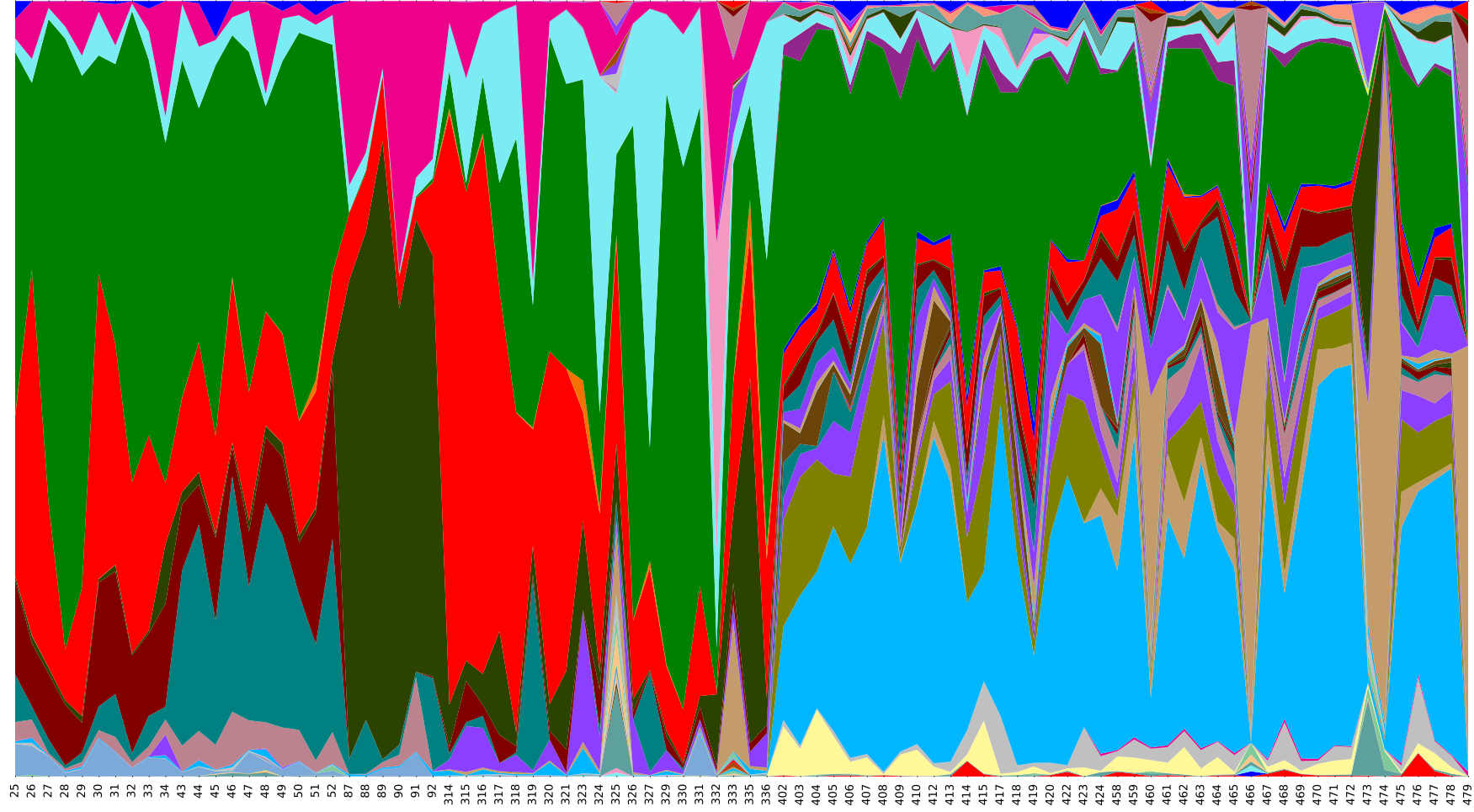

Supplement: Supplementary file 9 [file DataSheet4.ZIP › Supplemental_File_4_AviaryBarn_EnviroAgeGrp_TaxaSummaries/charts/iniPk8CaDguLU5e8C9t8M4cD29R3zf.pdf]

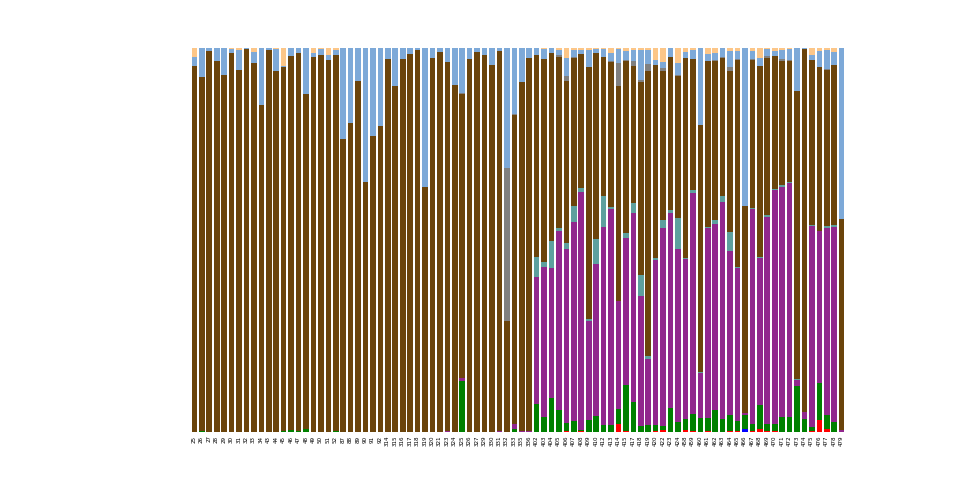

Supplement: Supplementary file 9 [file DataSheet4.ZIP › Supplemental_File_4_AviaryBarn_EnviroAgeGrp_TaxaSummaries/charts/kOzZ3DSa8SfXhFZwPlacmZaicNST0s.png]

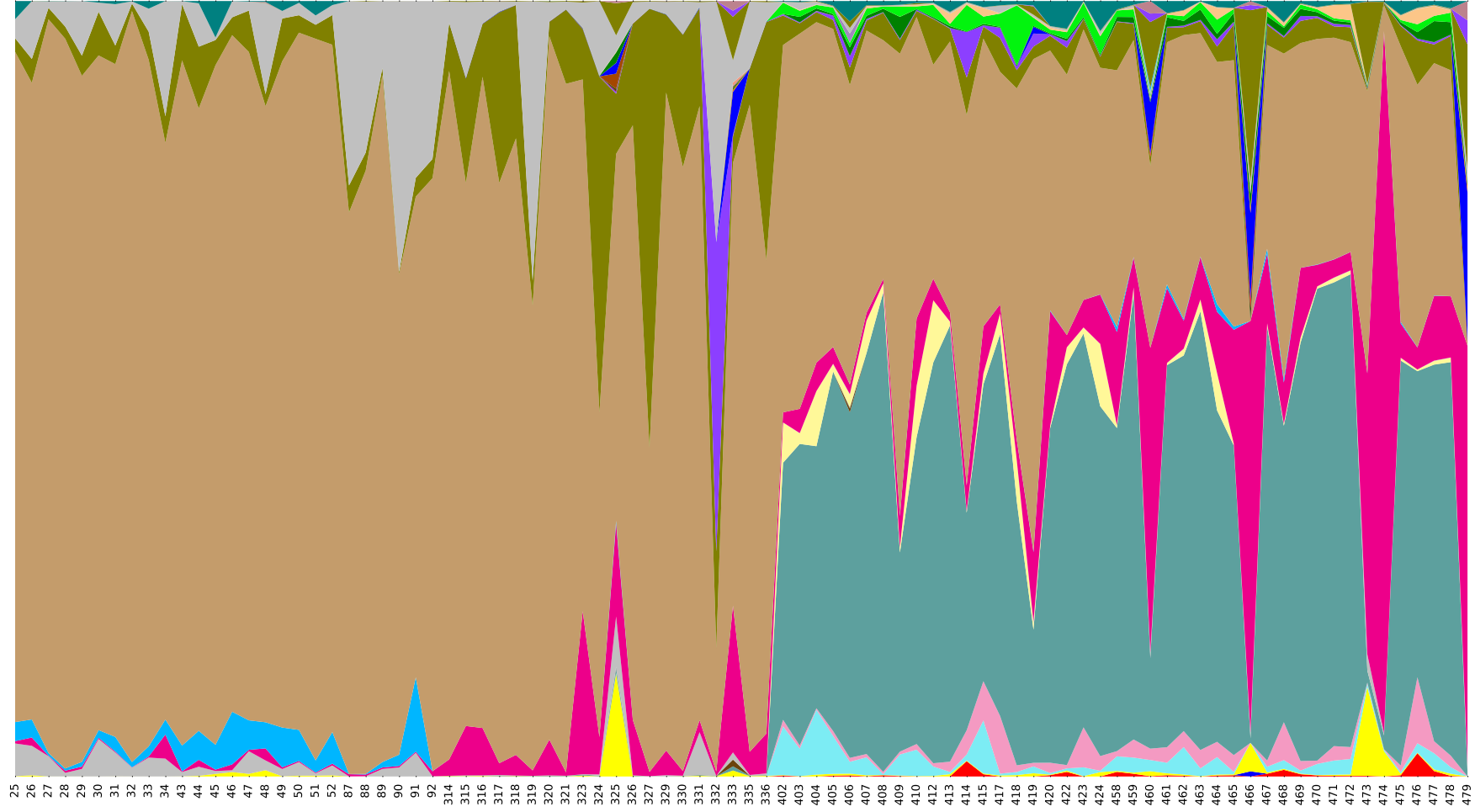

Supplement: Supplementary file 9 [file DataSheet4.ZIP › Supplemental_File_4_AviaryBarn_EnviroAgeGrp_TaxaSummaries/charts/PjzH7yNH3tyTOKLe9DT38cyQlOSR3A.pdf]

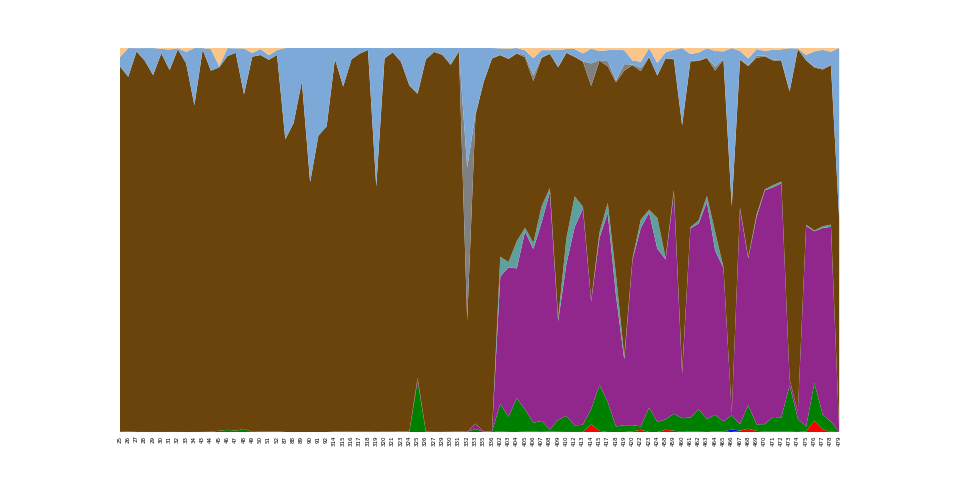

Supplement: Supplementary file 9 [file DataSheet4.ZIP › Supplemental_File_4_AviaryBarn_EnviroAgeGrp_TaxaSummaries/charts/QpI1KyZKNdTzW00owO0y5KZGALA7JF.png]

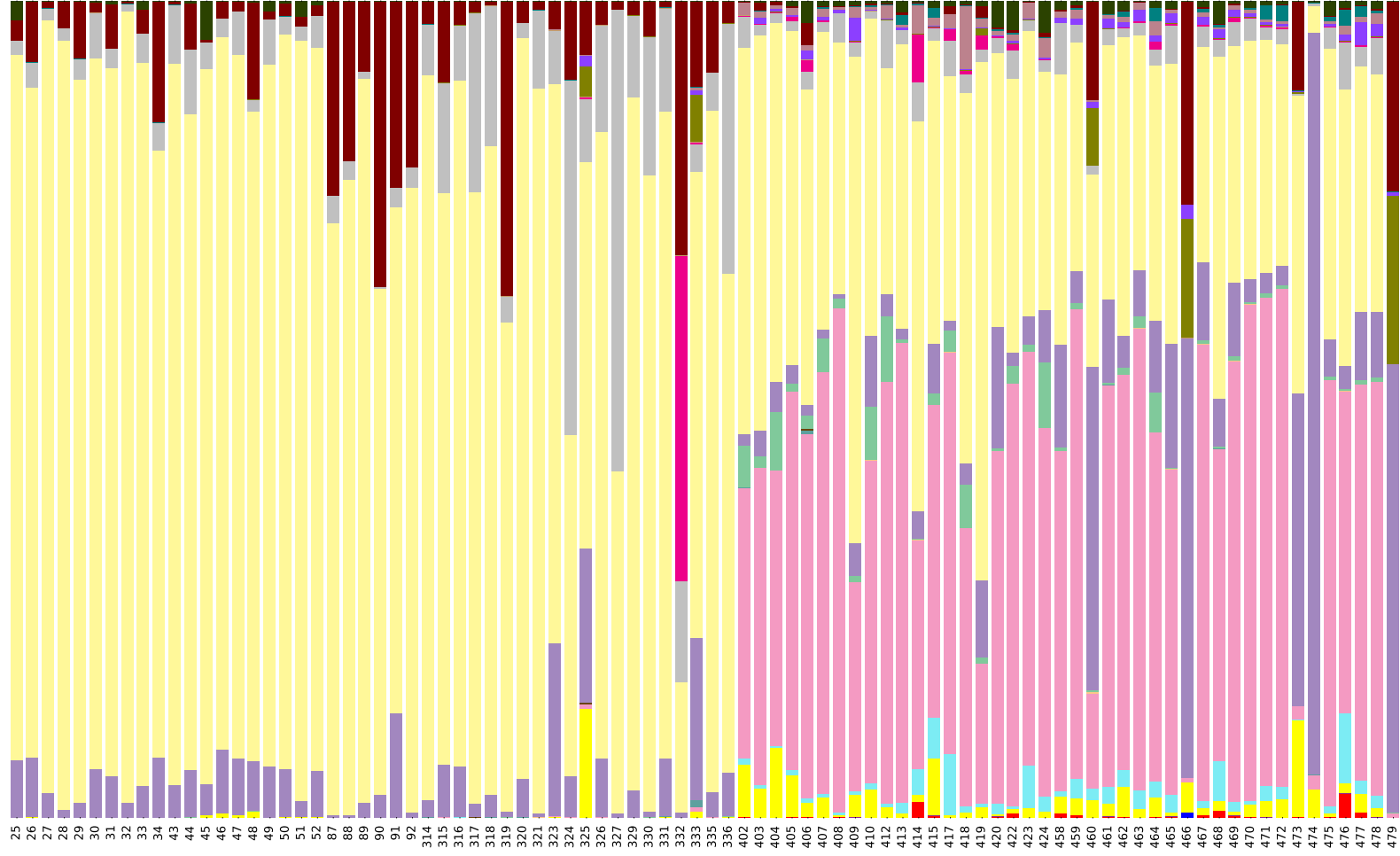

Supplement: Supplementary file 9 [file DataSheet4.ZIP › Supplemental_File_4_AviaryBarn_EnviroAgeGrp_TaxaSummaries/charts/ReUs0DTFeeJG2oK0jEPsP3egZMabiB.pdf]

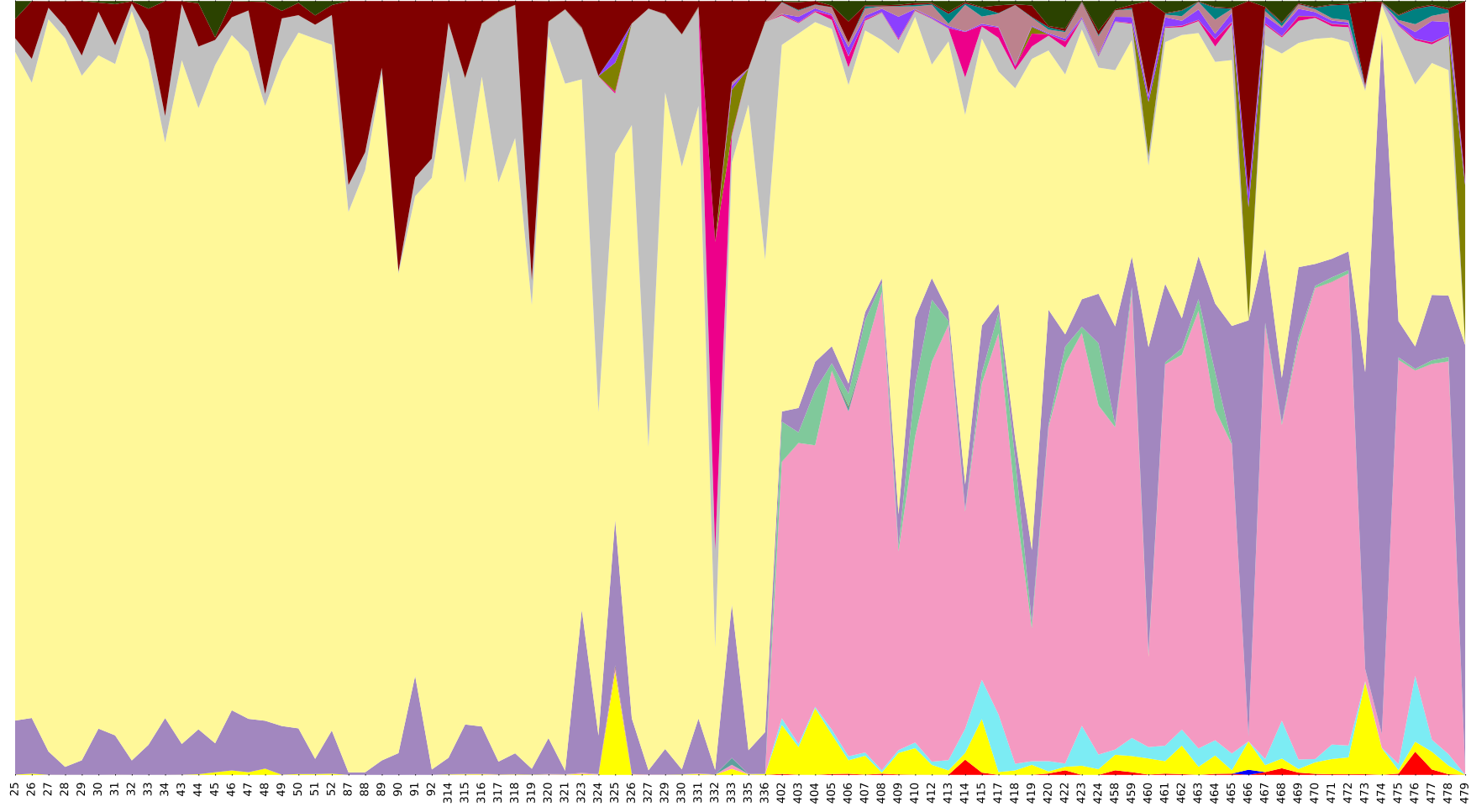

Supplement: Supplementary file 9 [file DataSheet4.ZIP › Supplemental_File_4_AviaryBarn_EnviroAgeGrp_TaxaSummaries/charts/Wi2WJz4YarsDBuO4U88aA7O1Wg1dLH.pdf]

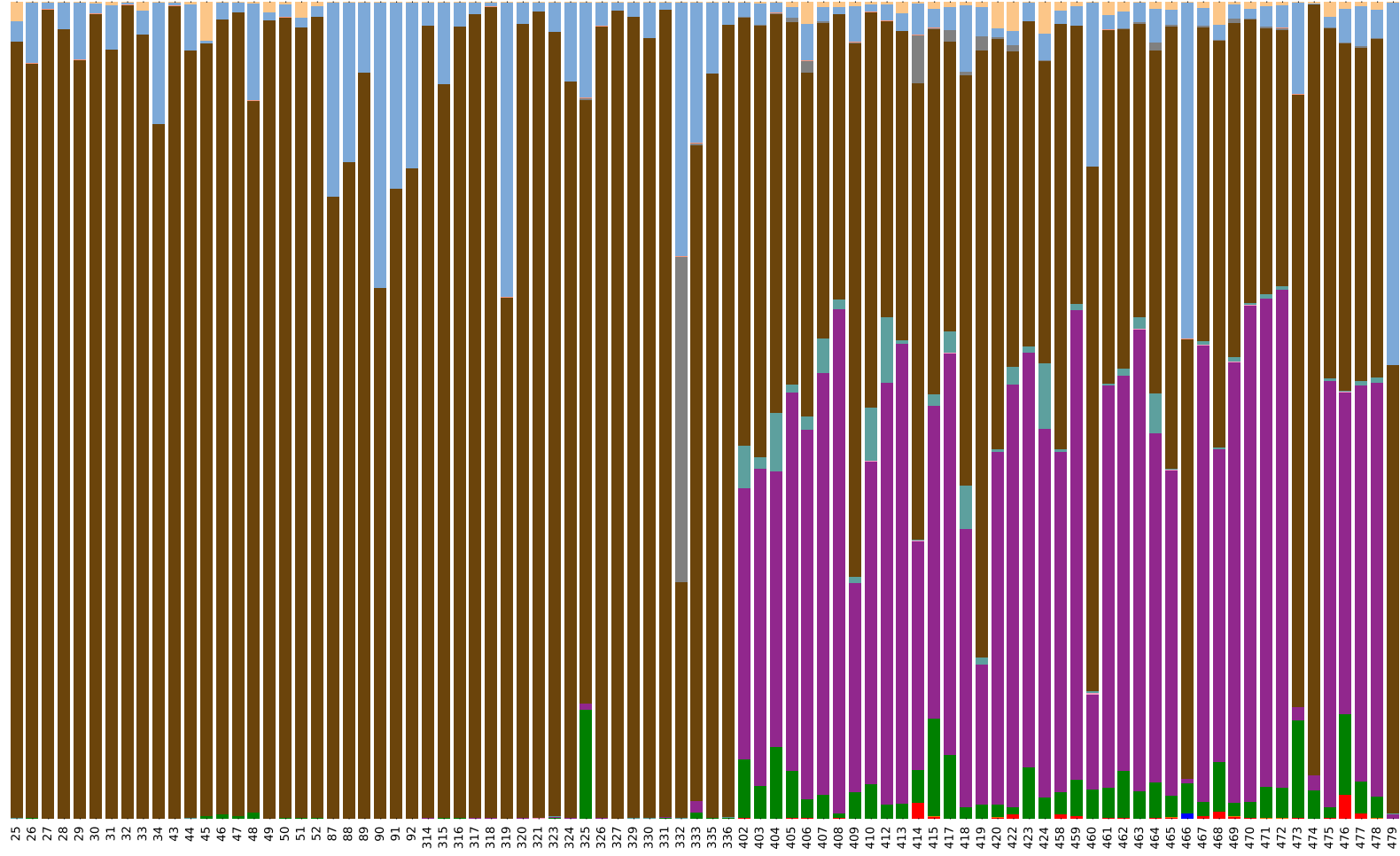

Supplement: Supplementary file 9 [file DataSheet4.ZIP › Supplemental_File_4_AviaryBarn_EnviroAgeGrp_TaxaSummaries/charts/y5195zIx0tT1cr2ttSoEaZkxEtOCR7.pdf]

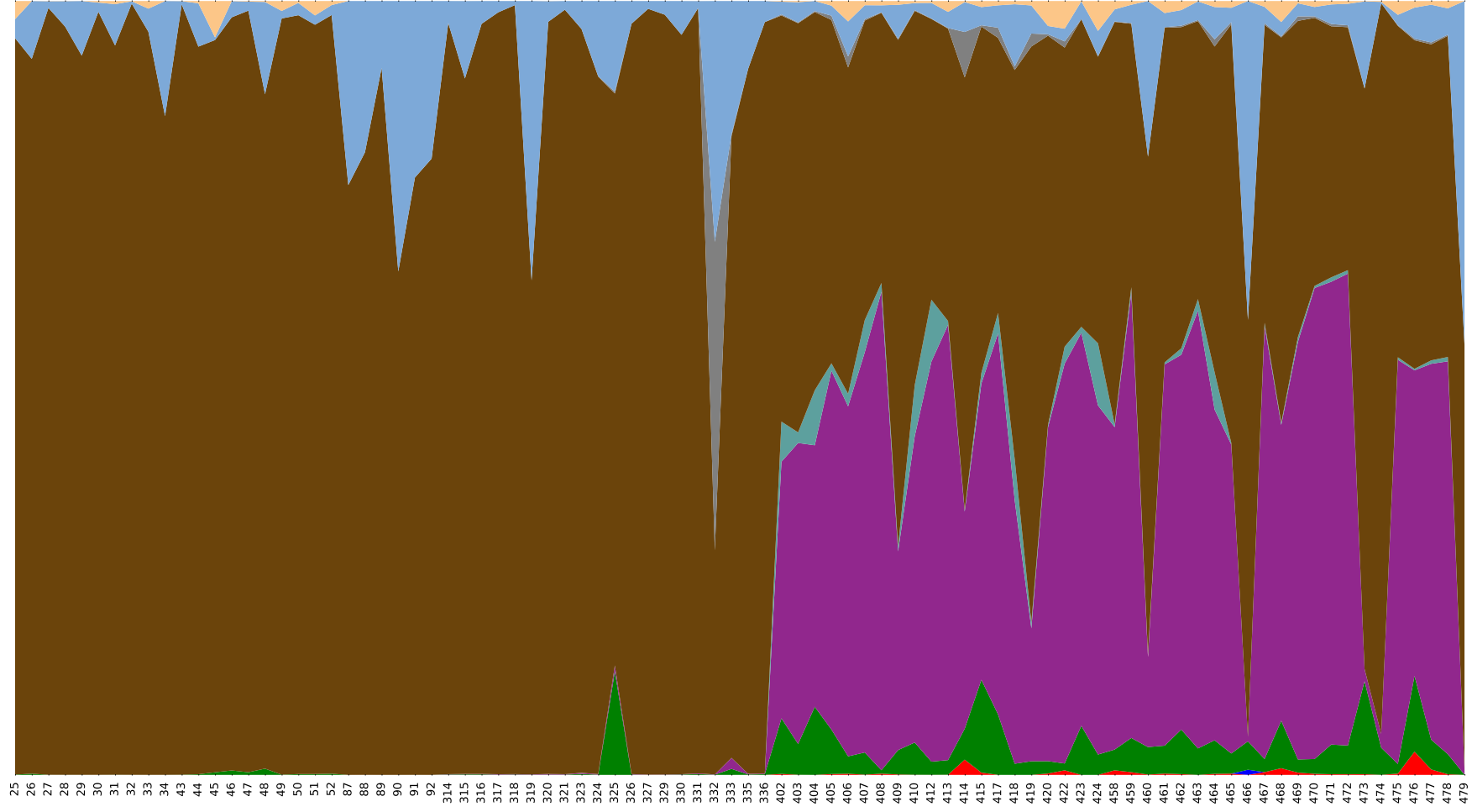

Supplement: Supplementary file 9 [file DataSheet4.ZIP › Supplemental_File_4_AviaryBarn_EnviroAgeGrp_TaxaSummaries/charts/YhHzipN1DY3xtJsgDIwIga7GuGZi6P.pdf]

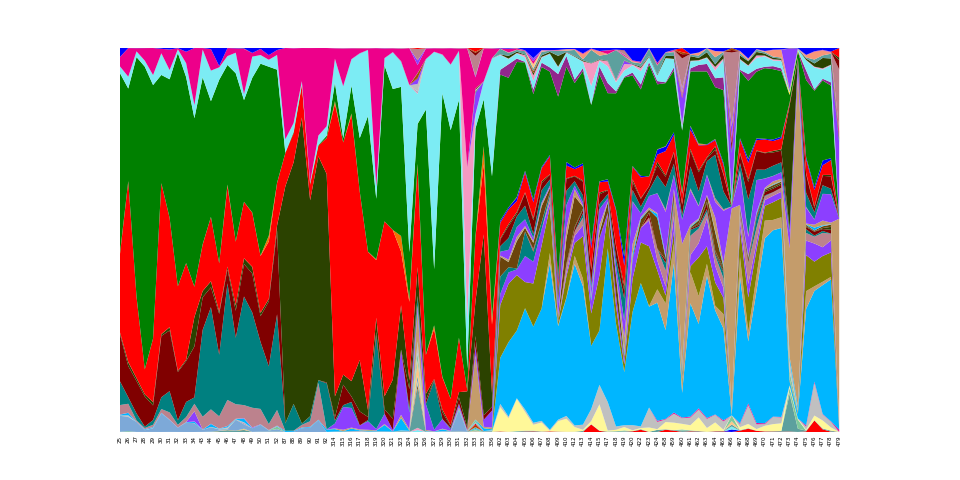

Supplement: Supplementary file 9 [file DataSheet4.ZIP › Supplemental_File_4_AviaryBarn_EnviroAgeGrp_TaxaSummaries/charts/YSkdFRr3byBZrolNmeFqSo937WHy9O.png]

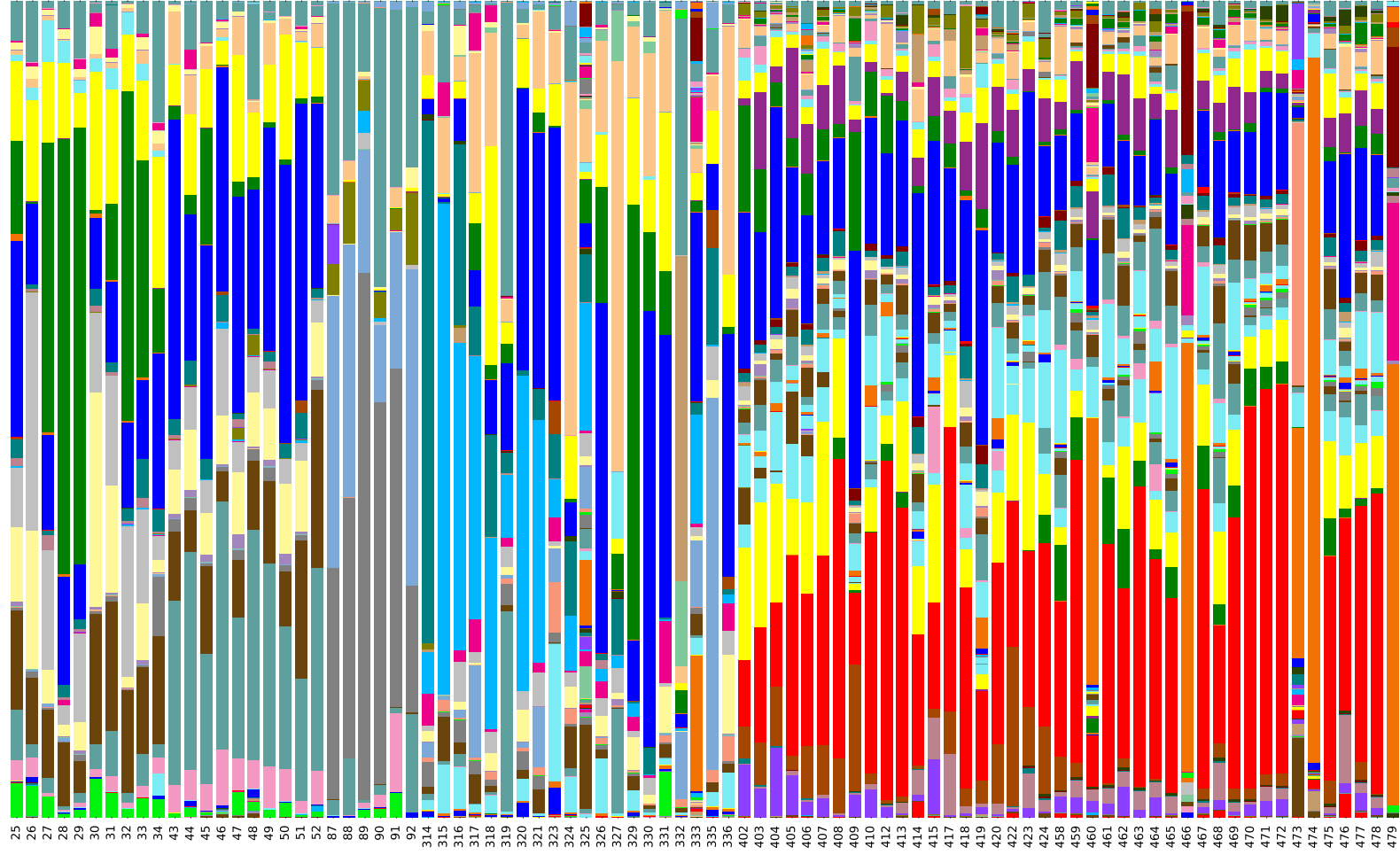

Supplement: Supplementary file 9 [file DataSheet4.ZIP › Supplemental_File_4_AviaryBarn_EnviroAgeGrp_TaxaSummaries/charts/ZgcmmMyDoO9oBJzoNMGjkYBILxy0Kf.pdf]

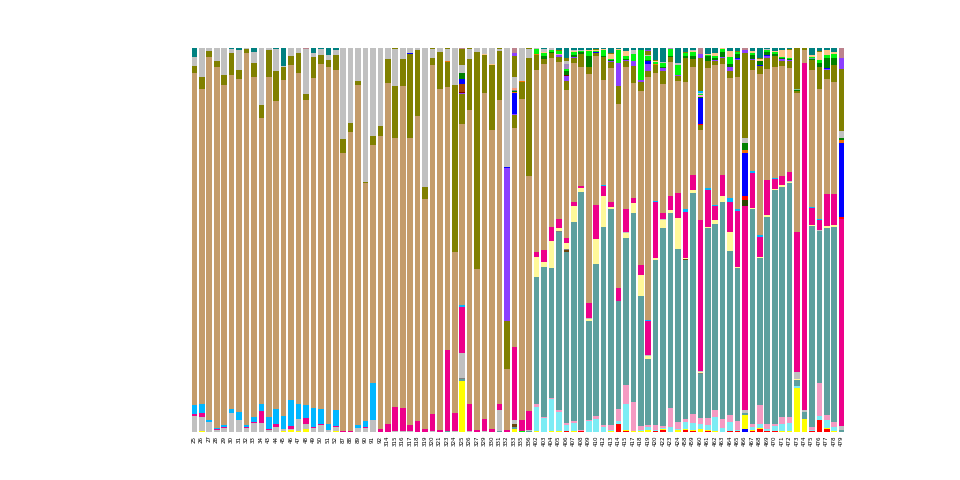

Supplement: Supplementary file 9 [file DataSheet4.ZIP › Supplemental_File_4_AviaryBarn_EnviroAgeGrp_TaxaSummaries/charts/zMBwDAZi7QzgzYpPsx6JUAKpSfuruY.png]

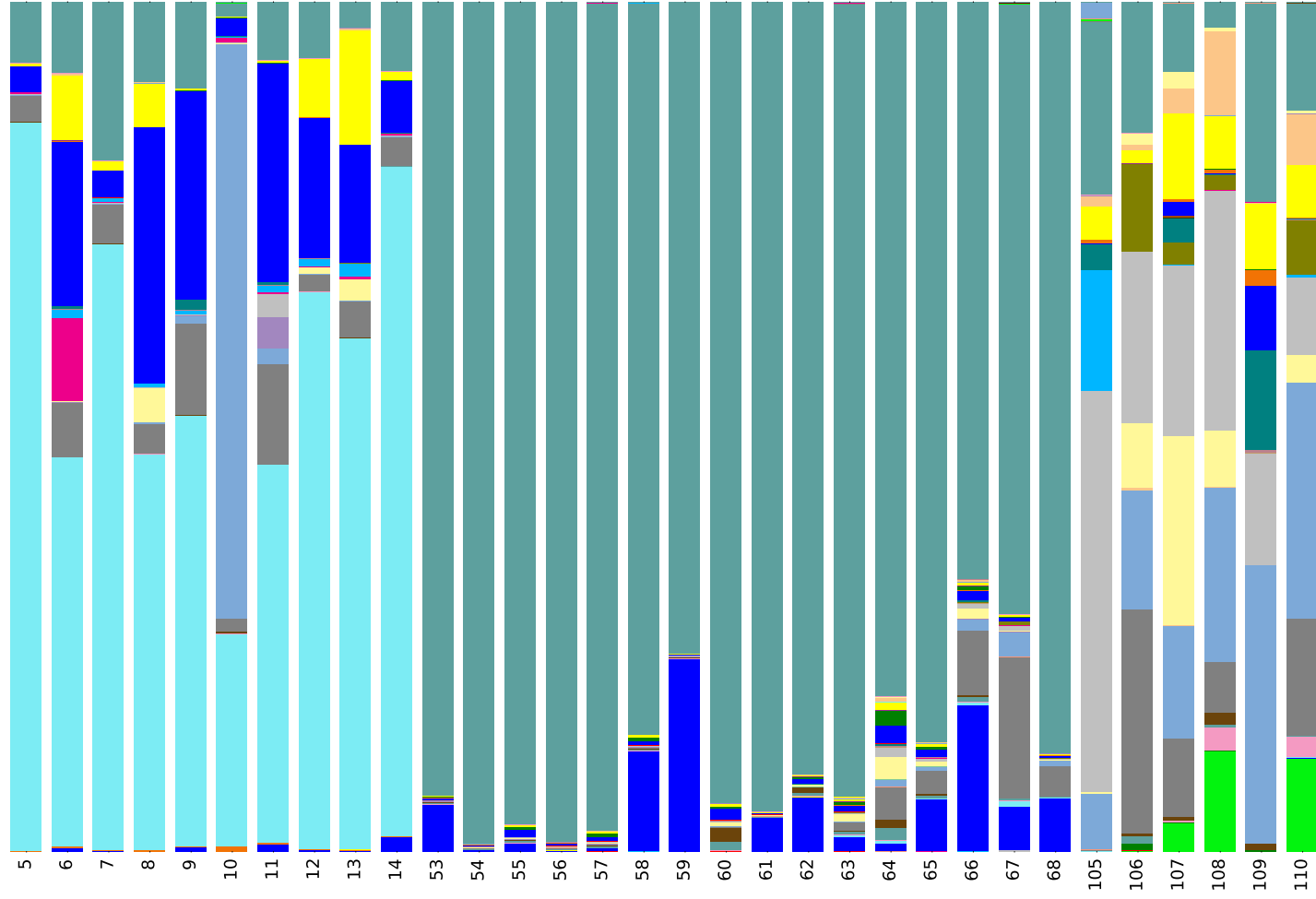

Supplement: Supplementary file 10 [file DataSheet5.ZIP › Supplemental_File_4_AviaryStudies_Days1-8_TaxaSummaries/charts/0C0DzbexfQiXCmS6qTzc0UlFwYraC6.pdf]

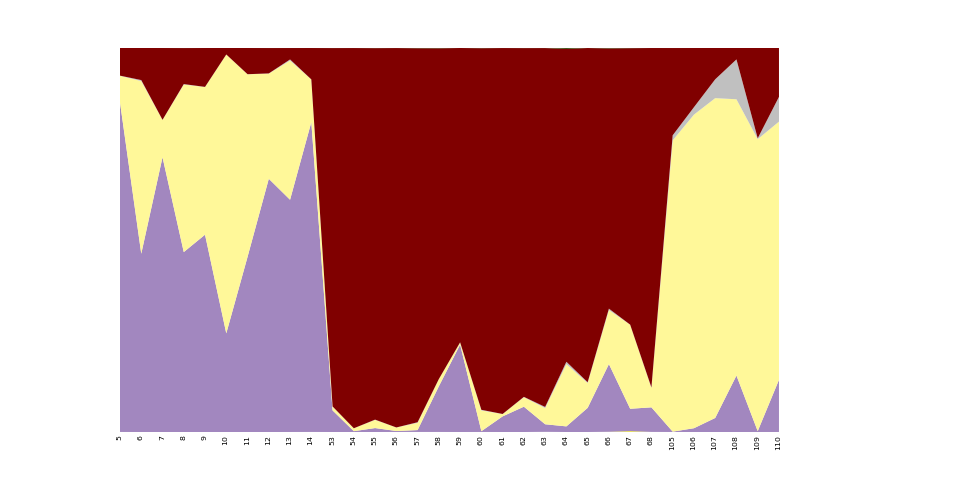

Supplement: Supplementary file 10 [file DataSheet5.ZIP › Supplemental_File_4_AviaryStudies_Days1-8_TaxaSummaries/charts/0Sc4sK7widuTIS5C3jucgZOCsRIFCi.png]

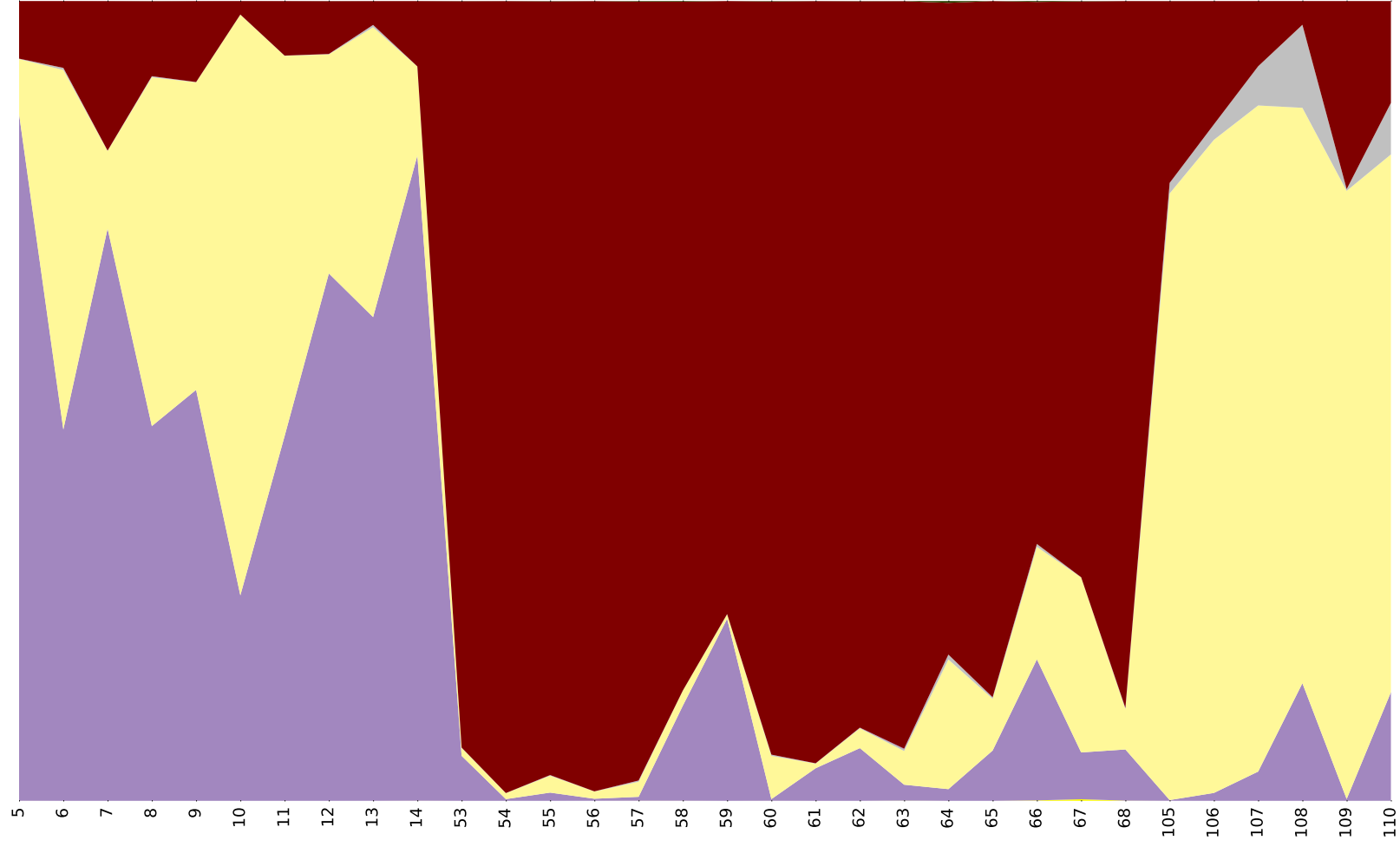

Supplement: Supplementary file 10 [file DataSheet5.ZIP › Supplemental_File_4_AviaryStudies_Days1-8_TaxaSummaries/charts/82JGORUgmmHeRYSt1WZ1hxR6d7EEug.pdf]

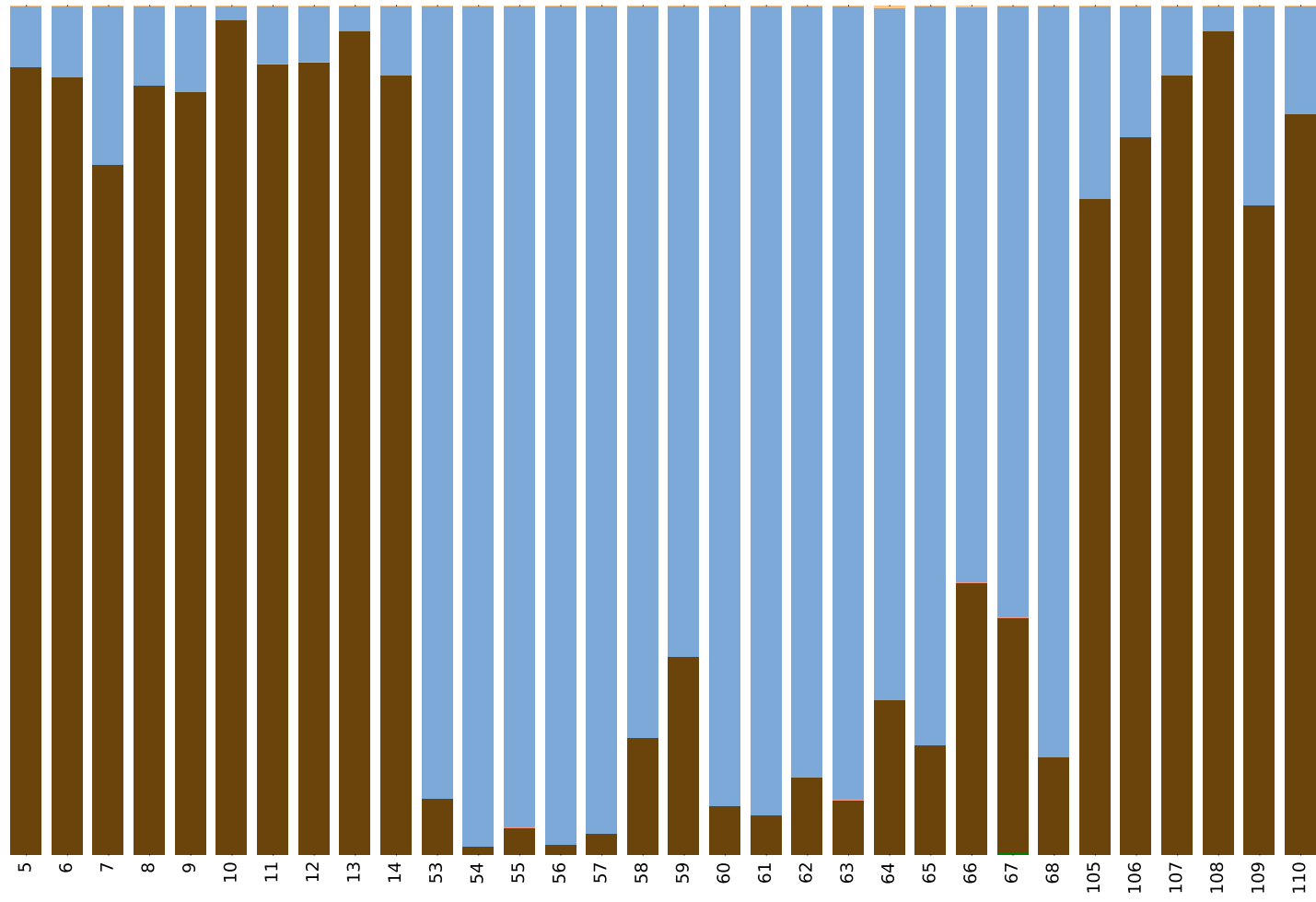

Supplement: Supplementary file 10 [file DataSheet5.ZIP › Supplemental_File_4_AviaryStudies_Days1-8_TaxaSummaries/charts/CeTxmeAex4KA3M8F2oJgh1s8em7mQT.pdf]

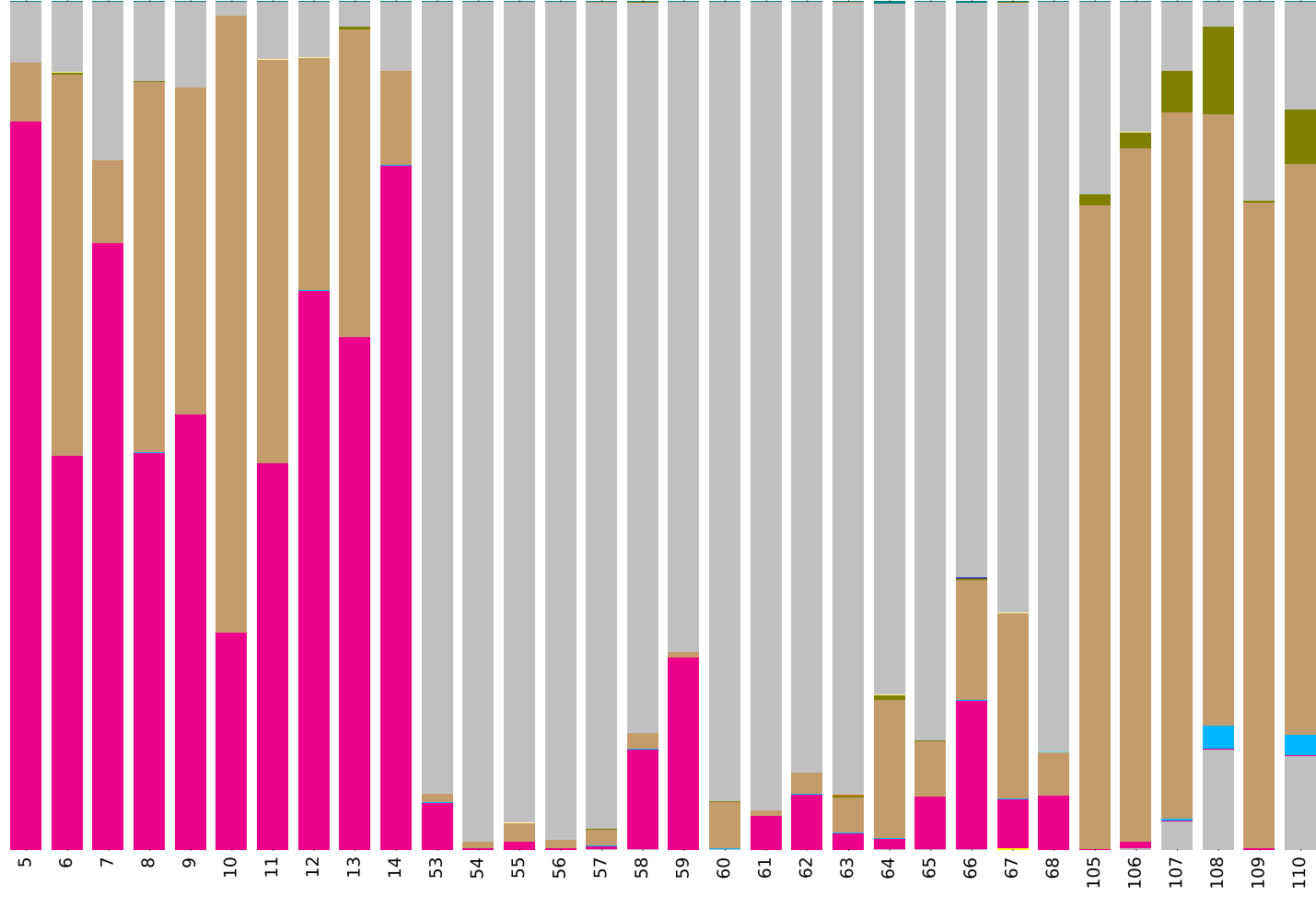

Supplement: Supplementary file 10 [file DataSheet5.ZIP › Supplemental_File_4_AviaryStudies_Days1-8_TaxaSummaries/charts/cxDjA42O8xjuhimzDMAD0QwruG7Feo.pdf]

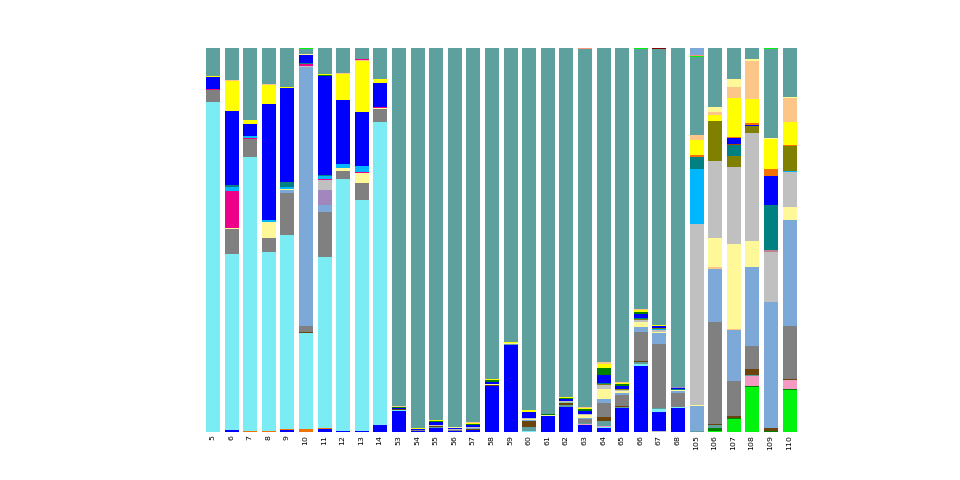

Supplement: Supplementary file 10 [file DataSheet5.ZIP › Supplemental_File_4_AviaryStudies_Days1-8_TaxaSummaries/charts/DD7IrfA8oopBb2cQzigketiXm8dkEq.png]

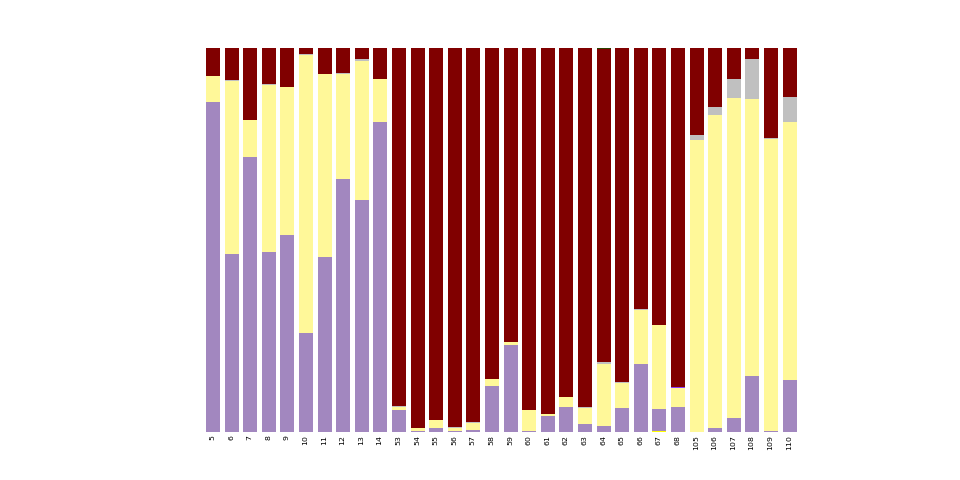

Supplement: Supplementary file 10 [file DataSheet5.ZIP › Supplemental_File_4_AviaryStudies_Days1-8_TaxaSummaries/charts/fHCYyBGME4ZNf7l1ZLxPiyDTqxhAIS.png]

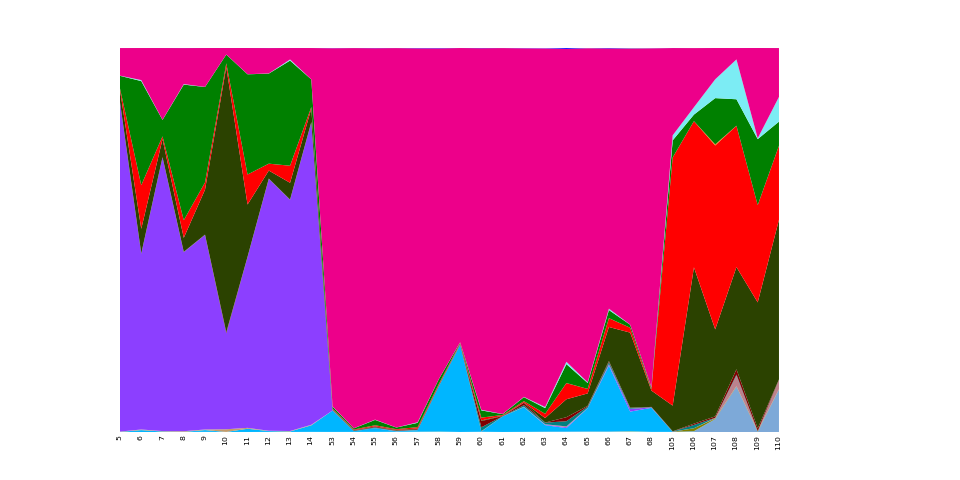

Supplement: Supplementary file 10 [file DataSheet5.ZIP › Supplemental_File_4_AviaryStudies_Days1-8_TaxaSummaries/charts/gKFrozjQ37nO3MlkagNdJlfnKsFndB.png]

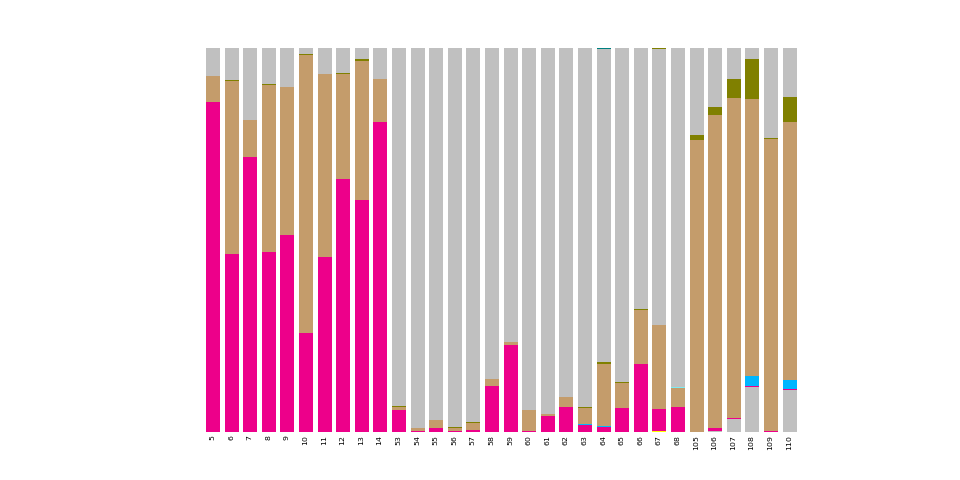

Supplement: Supplementary file 10 [file DataSheet5.ZIP › Supplemental_File_4_AviaryStudies_Days1-8_TaxaSummaries/charts/HTQQcri5ijR3ZwCJtF9gspbT7GJq0C.png]

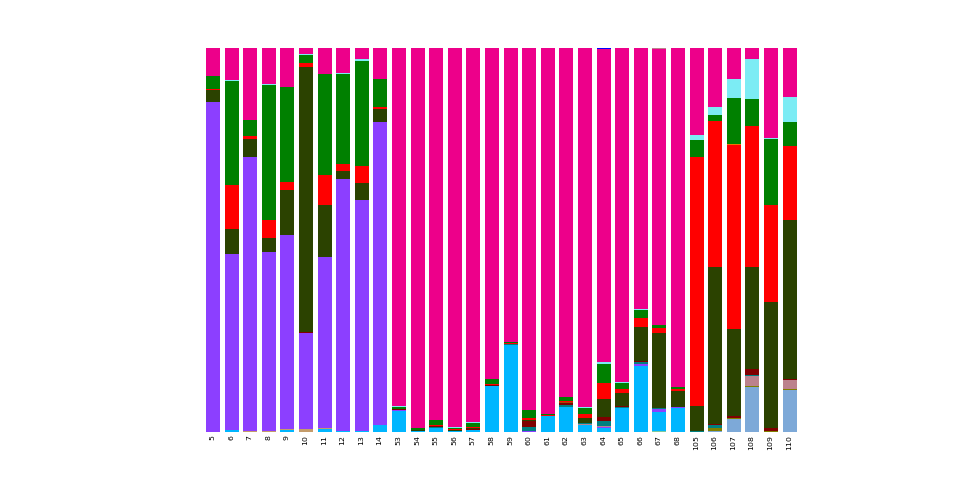

Supplement: Supplementary file 10 [file DataSheet5.ZIP › Supplemental_File_4_AviaryStudies_Days1-8_TaxaSummaries/charts/N9rn0zQKoiwhkfy0hbEfgP2gYceMS6.png]

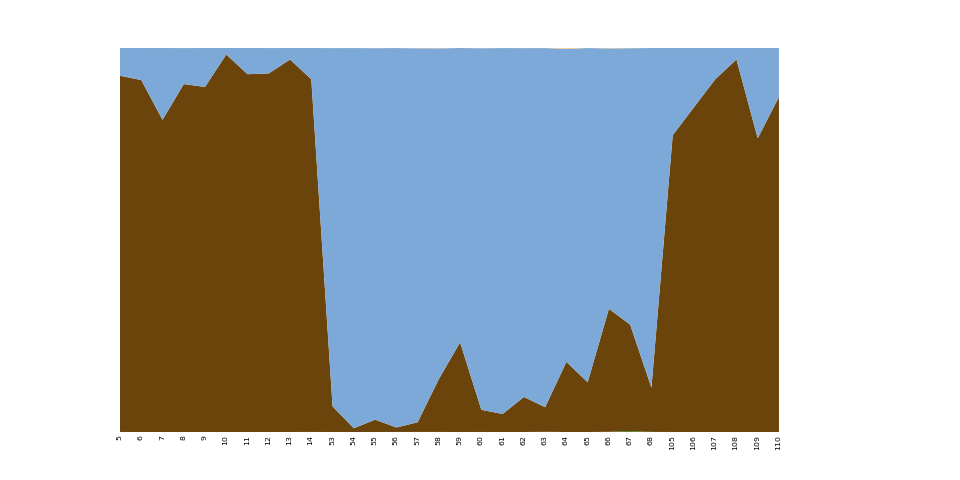

Supplement: Supplementary file 10 [file DataSheet5.ZIP › Supplemental_File_4_AviaryStudies_Days1-8_TaxaSummaries/charts/q3b8zz8WSwR9cl20MOTYtl5IbcPABR.png]

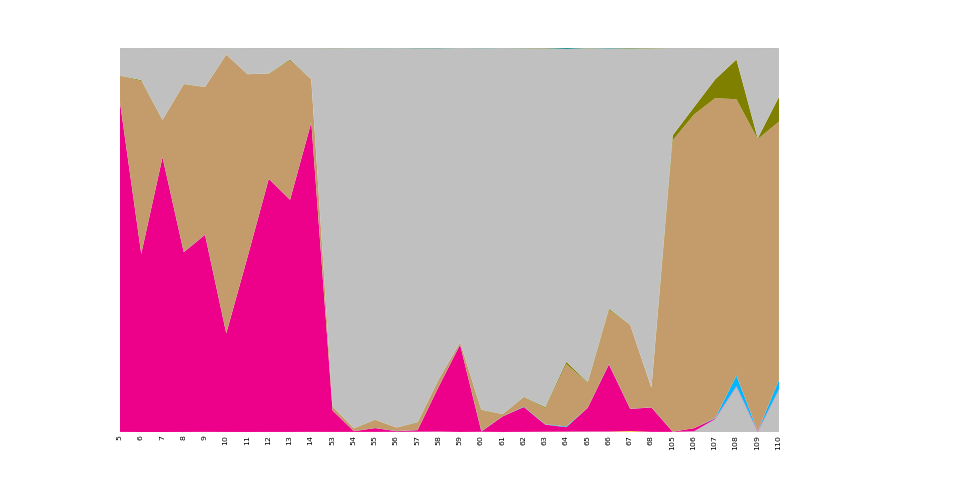

Supplement: Supplementary file 10 [file DataSheet5.ZIP › Supplemental_File_4_AviaryStudies_Days1-8_TaxaSummaries/charts/QbAH63R78XZbPEcwMmoc8d8aZeYK1n.png]

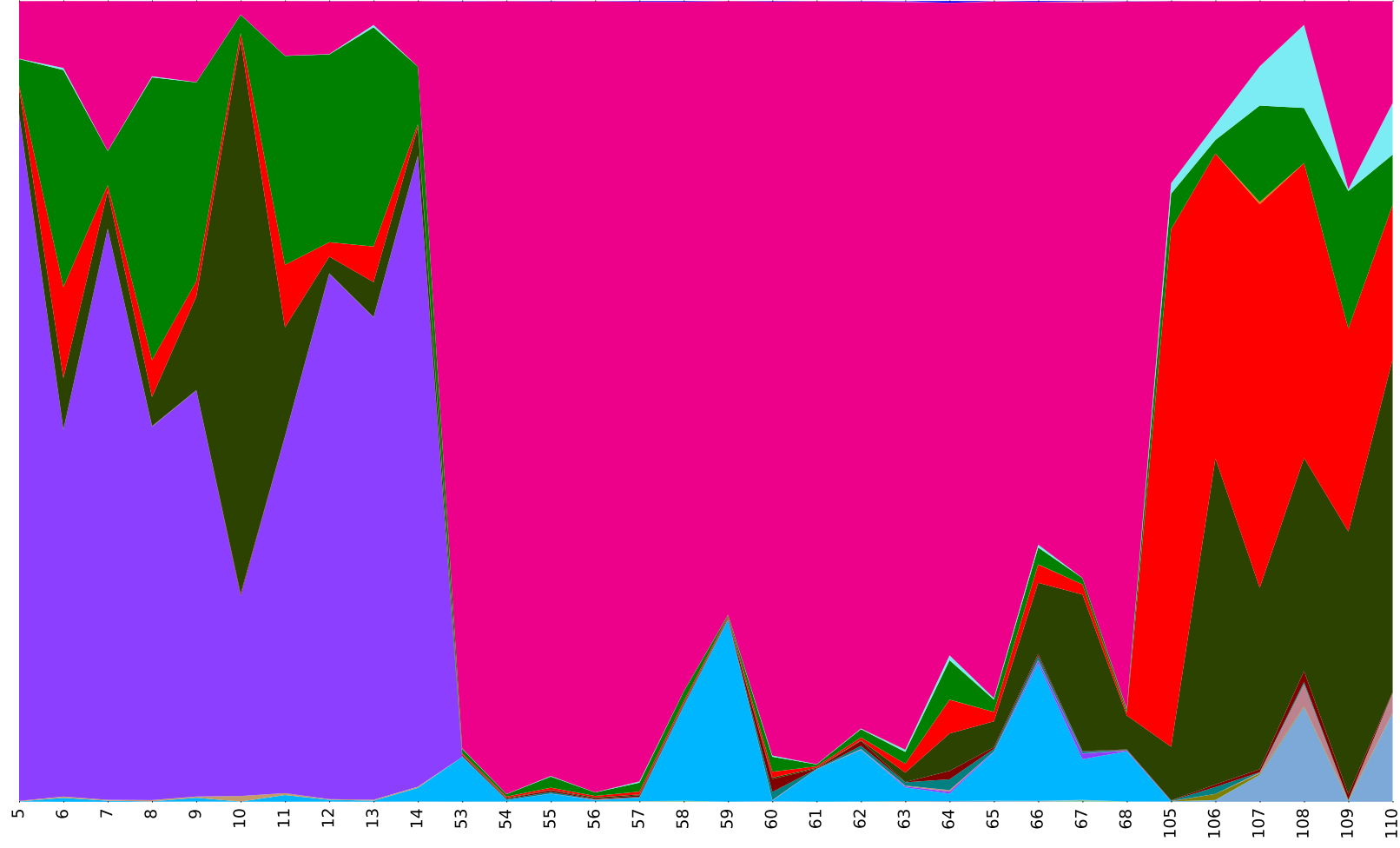

Supplement: Supplementary file 10 [file DataSheet5.ZIP › Supplemental_File_4_AviaryStudies_Days1-8_TaxaSummaries/charts/r73ieC6KBZexQu5u8lC6HjphSwsTPe.pdf]

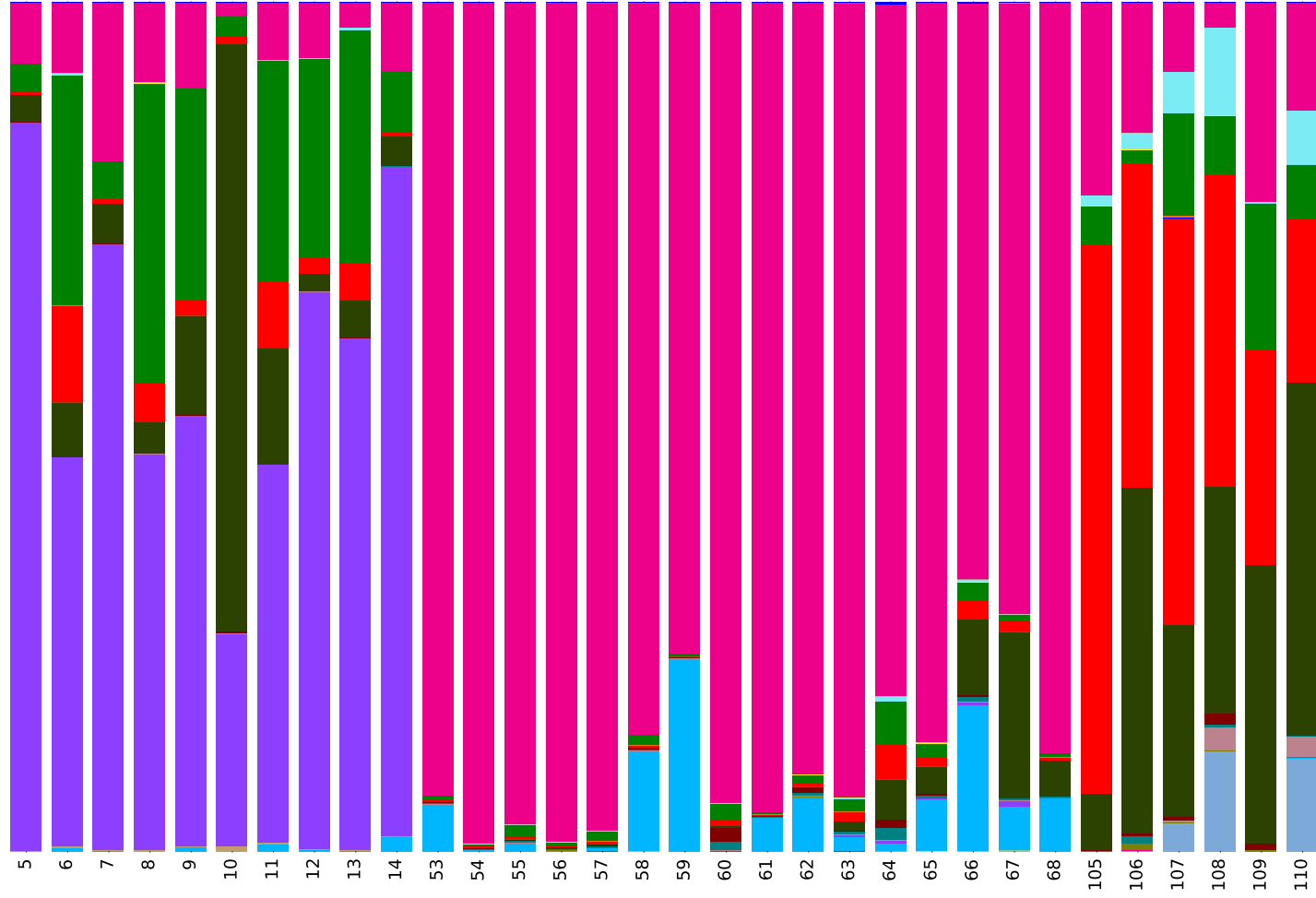

Supplement: Supplementary file 10 [file DataSheet5.ZIP › Supplemental_File_4_AviaryStudies_Days1-8_TaxaSummaries/charts/R7dFjOm5uX9TLid6CG7sK2NO2qHh1E.pdf]

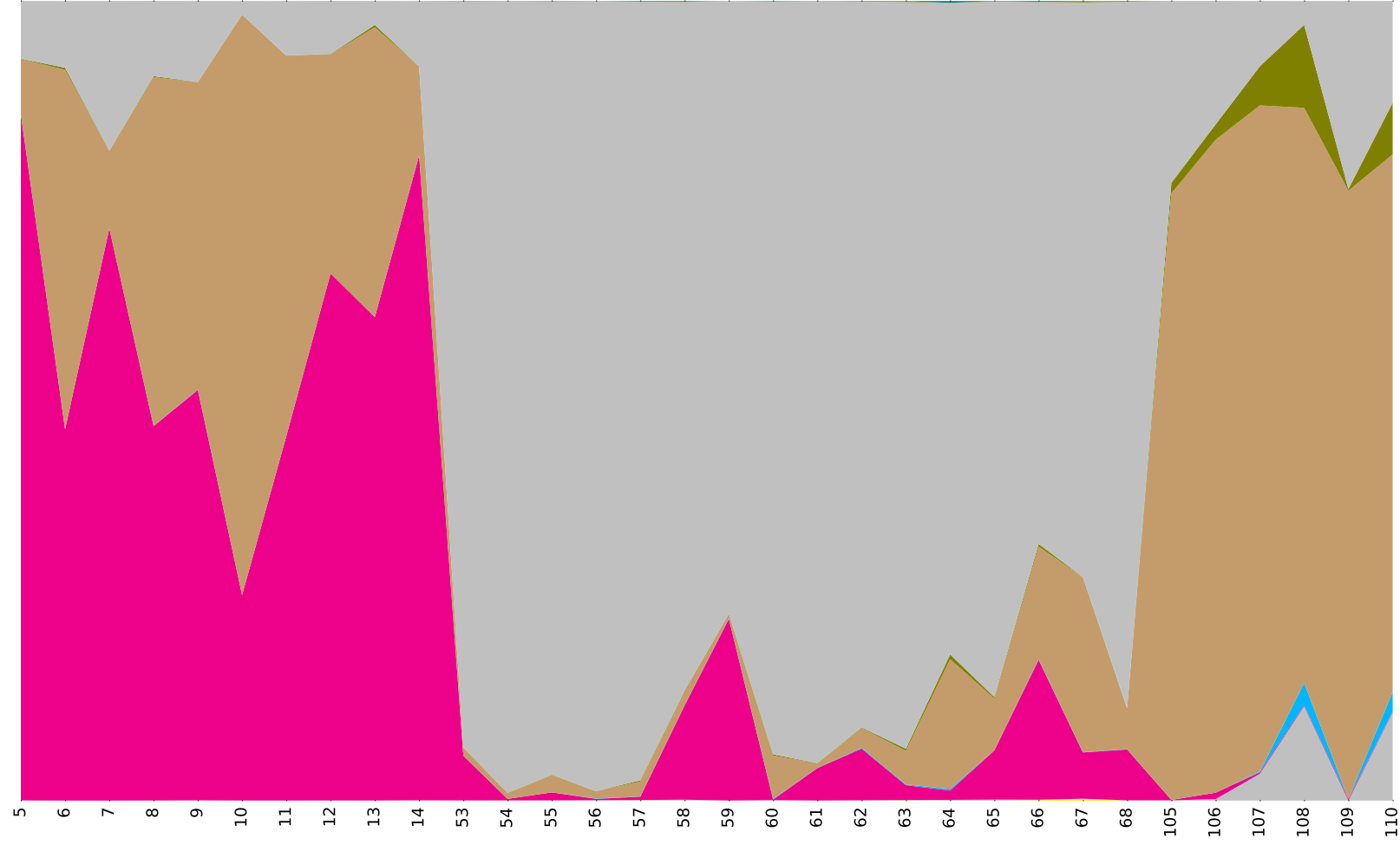

Supplement: Supplementary file 10 [file DataSheet5.ZIP › Supplemental_File_4_AviaryStudies_Days1-8_TaxaSummaries/charts/SXsXKDdq9mD5tdDDde6kNKk2iTqUUo.pdf]

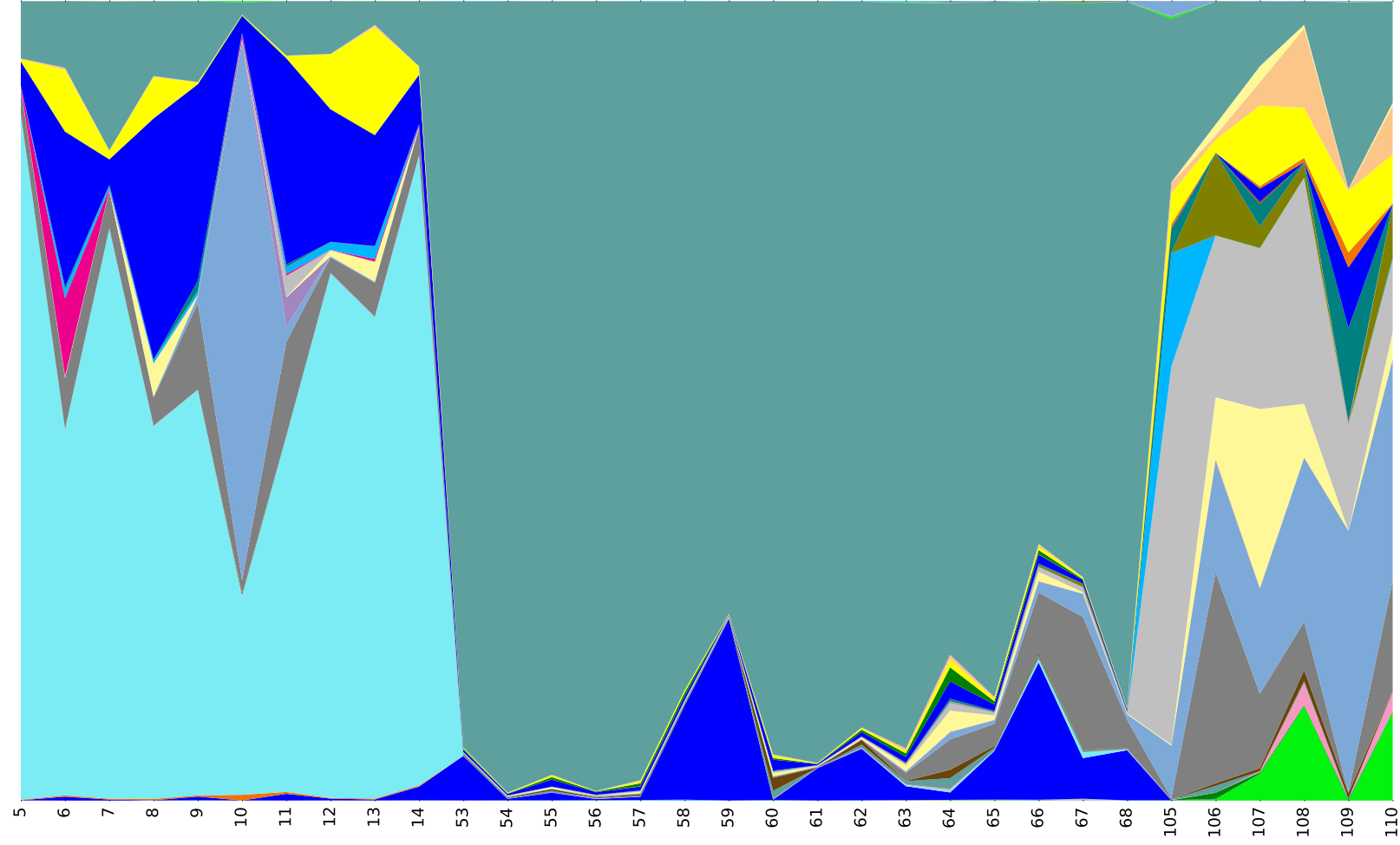

Supplement: Supplementary file 10 [file DataSheet5.ZIP › Supplemental_File_4_AviaryStudies_Days1-8_TaxaSummaries/charts/Sz36QBxqcsrGSqw1PzKQnxK8onhtPu.pdf]

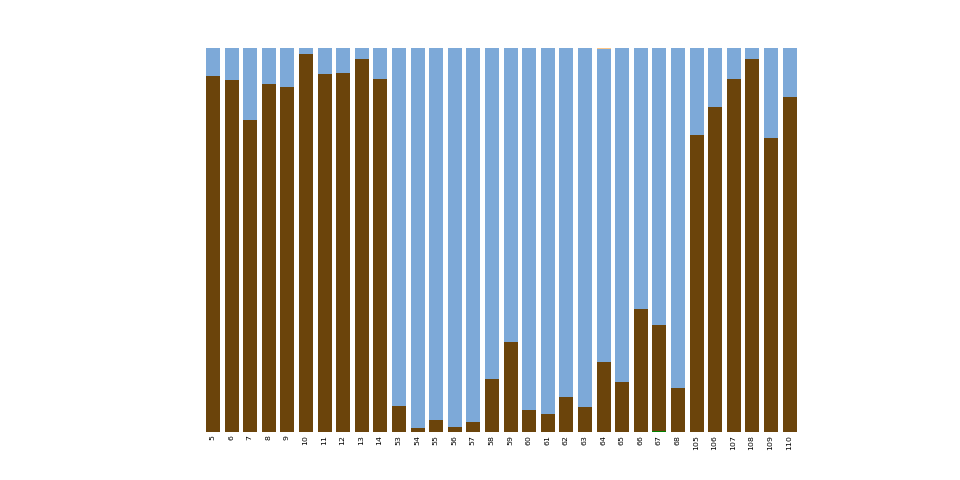

Supplement: Supplementary file 10 [file DataSheet5.ZIP › Supplemental_File_4_AviaryStudies_Days1-8_TaxaSummaries/charts/wQpcbNDkygC6LZXkzZ5rnclAogi2c7.png]

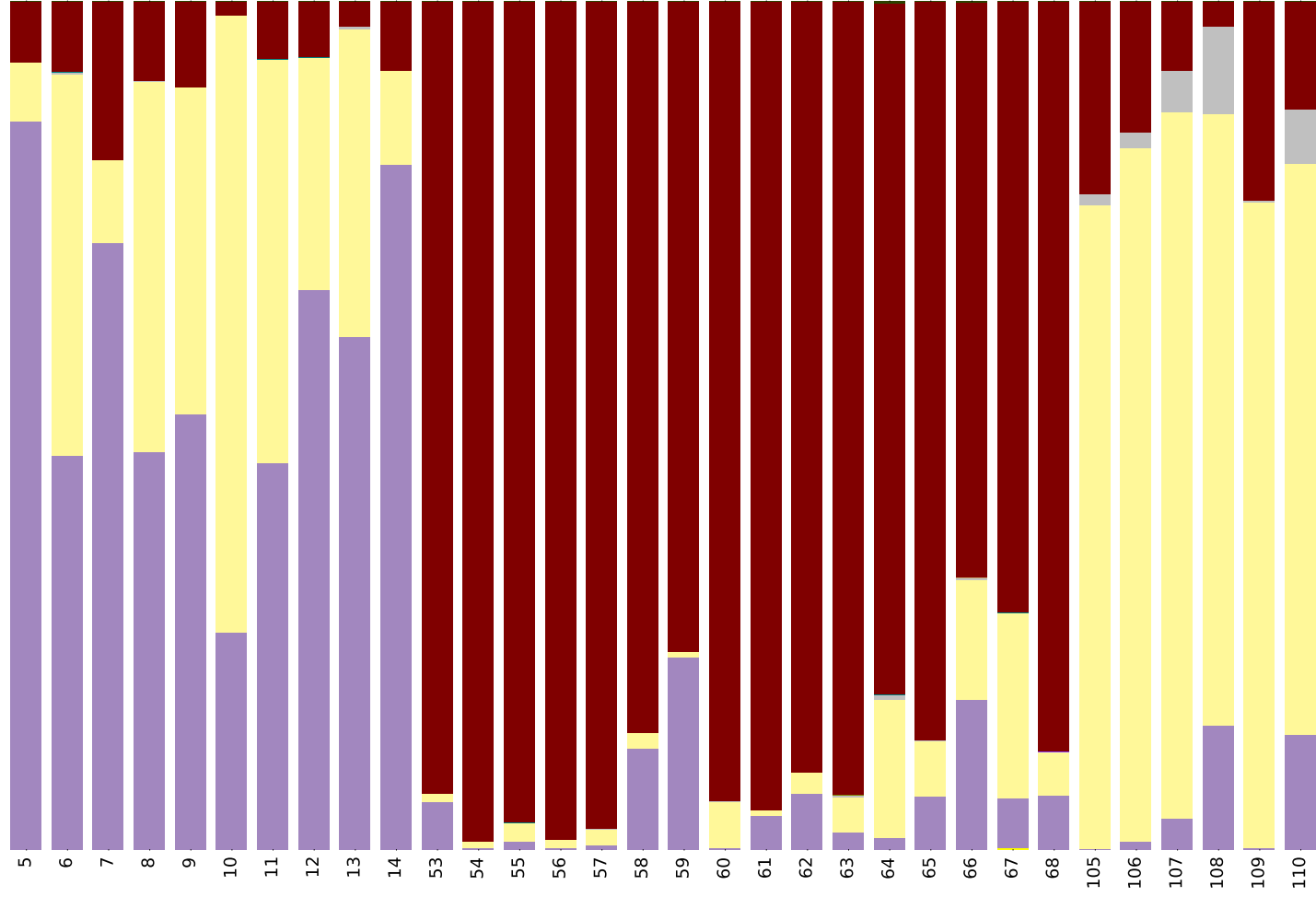

Supplement: Supplementary file 10 [file DataSheet5.ZIP › Supplemental_File_4_AviaryStudies_Days1-8_TaxaSummaries/charts/WwkoeaKO22qXLLQAWFbWy6us1etppd.pdf]

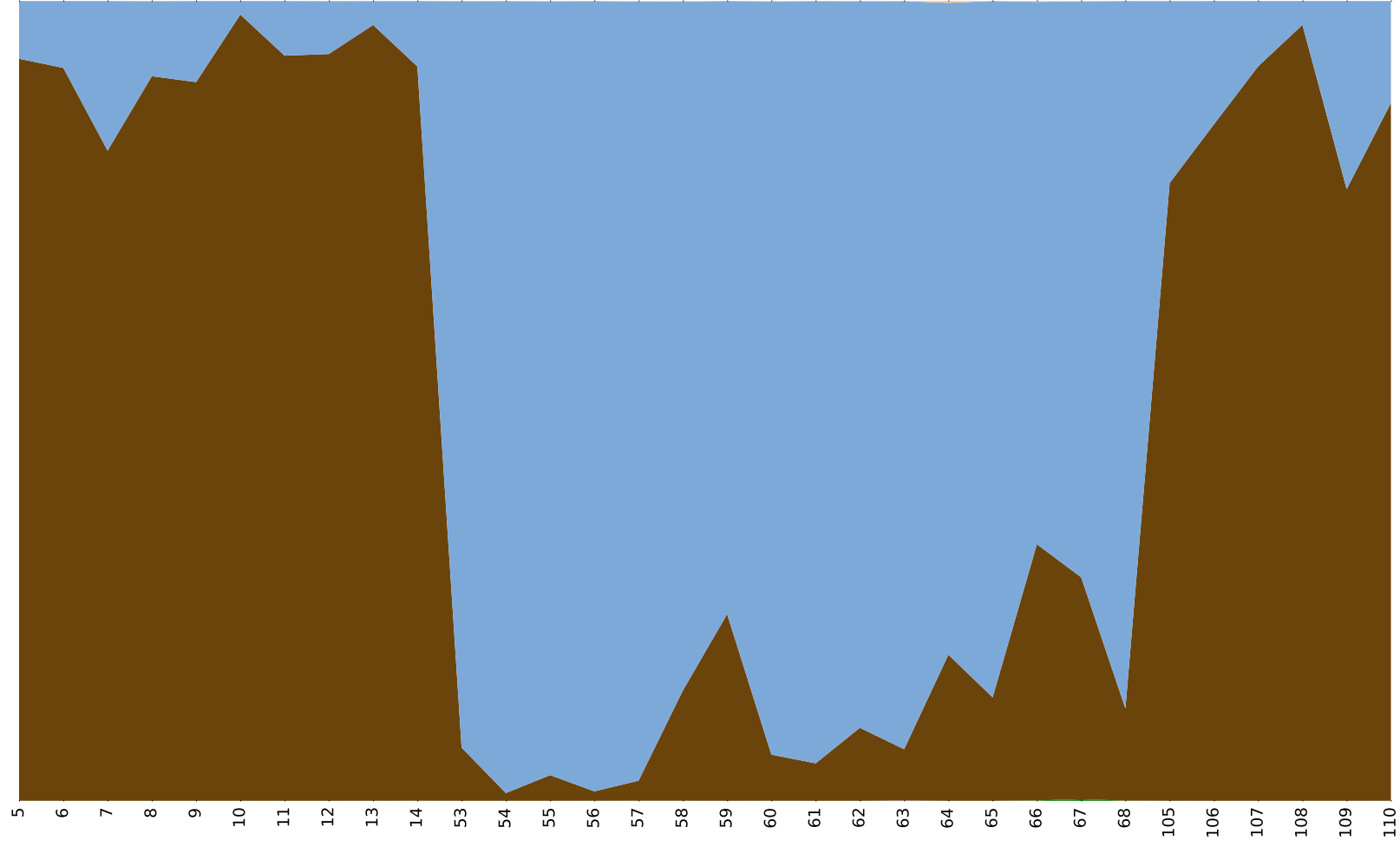

Supplement: Supplementary file 10 [file DataSheet5.ZIP › Supplemental_File_4_AviaryStudies_Days1-8_TaxaSummaries/charts/X0730W1PcKfZjQgSipQXh8zagLzcNu.pdf]
