# Supplementary material for: Characterization of Gut Microbiome Dynamics in Developing Pekin Ducks and Impact of Management System
Source: Front Microbiol. 2017 Jan 4;7:2125. doi: 10.3389/fmicb.2016.02125 (PMC5209349; doi:10.3389/fmicb.2016.02125)
Supplement: Supplementary file 7 [file DataSheet2.ZIP › Supplemental_File_2_AviaryStudy2_TaxaSummaries/charts/taX472TNERZLGY5iD8JkD7KZbFNOpZ_legend.pdf]

NOHIT;Other;Other;Other
k__Archaea;p__Crenarchaeota;c__Thaumarchaeota;o__Cenarchaeales
k__Bacteria;p__Acidobacteria;c__BPC102;o__MVS-40
k__Bacteria;p__Acidobacteria;c__DA052;o__Ellin6513
k__Bacteria;p__Actinobacteria;c__Acidimicrobiia;o__Acidimicrobiales
k__Bacteria;p__Actinobacteria;c__Actinobacteria;o__Actinomycetales
k__Bacteria;p__Actinobacteria;c__Actinobacteria;o__Bifidobacteriales
k__Bacteria;p__Actinobacteria;c__Coriobacteriia;o__Coriobacteriales
k__Bacteria;p__Bacteroidetes;c__Bacteroidia;o__Bacteroidales
k__Bacteria;p__Bacteroidetes;c__Flavobacteriia;o__Flavobacteriales
k__Bacteria;p__Bacteroidetes;c__Sphingobacteriia;o__Sphingobacteriales
k__Bacteria;p__Bacteroidetes;c__[Saprospirae];o__[Saprospirales]
k__Bacteria;p__Chlorobi;c__OPB56;o__
k__Bacteria;p__Chloroflexi;c__Anaerolineae;o__GCA004
k__Bacteria;p__Chloroflexi;c__Anaerolineae;o__SBR1031
k__Bacteria;p__Cyanobacteria;c__4C0d-2;o__MLE1-12
k__Bacteria;p__Cyanobacteria;c__Synechococcophycideae;o__Synechococcales
k__Bacteria;p__Deferribacteres;c__Deferribacteres;o__Deferribacterales
k__Bacteria;p__Firmicutes;c__Bacilli;o__Bacillales
k__Bacteria;p__Firmicutes;c__Bacilli;o__Lactobacillales
k__Bacteria;p__Firmicutes;c__Bacilli;o__Turicibacterales
k__Bacteria;p__Firmicutes;c__Clostridia;o__Clostridiales
k__Bacteria;p__Firmicutes;c__Erysipelotrichi;o__Erysipelotrichales
k__Bacteria;p__Fusobacteria;c__Fusobacteriia;o__Fusobacteriales
k__Bacteria;p__Gemmatimonadetes;c__Gemmatimonadetes;o__
k__Bacteria;p__Planctomycetes;c__Planctomycetia;o__Gemmatales
k__Bacteria;p__Proteobacteria;c__Alphaproteobacteria;o__Caulobacterales
k__Bacteria;p__Proteobacteria;c__Alphaproteobacteria;o__Rhizobiales
k__Bacteria;p__Proteobacteria;c__Alphaproteobacteria;o__Rhodobacterales
k__Bacteria;p__Proteobacteria;c__Alphaproteobacteria;o__Rhodospirillales
k__Bacteria;p__Proteobacteria;c__Alphaproteobacteria;o__Rickettsiales
k__Bacteria;p__Proteobacteria;c__Alphaproteobacteria;o__Sphingomonadales
k__Bacteria;p__Proteobacteria;c__Betaproteobacteria;o__Burkholderiales
k__Bacteria;p__Proteobacteria;c__Betaproteobacteria;o__Ellin6067
k__Bacteria;p__Proteobacteria;c__Betaproteobacteria;o__IS-44
k__Bacteria;p__Proteobacteria;c__Betaproteobacteria;o__MKC10
k__Bacteria;p__Proteobacteria;c__Betaproteobacteria;o__Methylophilales
k__Bacteria;p__Proteobacteria;c__Betaproteobacteria;o__Neisseriales
k__Bacteria;p__Proteobacteria;c__Betaproteobacteria;o__Nitrosomonadales
k__Bacteria;p__Proteobacteria;c__Betaproteobacteria;o__Rhodocyclales
k__Bacteria;p__Proteobacteria;c__Deltaproteobacteria;o__Desulfovibrionales
k__Bacteria;p__Proteobacteria;c__Deltaproteobacteria;o__Myxococcales
k__Bacteria;p__Proteobacteria;c__Deltaproteobacteria;o__Sva0853
k__Bacteria;p__Proteobacteria;c__Epsilonproteobacteria;o__Campylobacterales
k__Bacteria;p__Proteobacteria;c__Gammaproteobacteria;Other
k__Bacteria;p__Proteobacteria;c__Gammaproteobacteria;o__Aeromonadales
k__Bacteria;p__Proteobacteria;c__Gammaproteobacteria;o__Alteromonadales
k__Bacteria;p__Proteobacteria;c__Gammaproteobacteria;o__Enterobacteriales
k__Bacteria;p__Proteobacteria;c__Gammaproteobacteria;o__Legionellales
k__Bacteria;p__Proteobacteria;c__Gammaproteobacteria;o__Methylococcales
k__Bacteria;p__Proteobacteria;c__Gammaproteobacteria;o__Pasteurellales
k__Bacteria;p__Proteobacteria;c__Gammaproteobacteria;o__Pseudomonadales
k__Bacteria;p__Proteobacteria;c__Gammaproteobacteria;o__Vibrionales
k__Bacteria;p__Proteobacteria;c__Gammaproteobacteria;o__Xanthomonadales
k__Bacteria;p__Tenericutes;c__Mollicutes;o__RF39
