# Supplementary material for: Characterization of Gut Microbiome Dynamics in Developing Pekin Ducks and Impact of Management System
Source: Front Microbiol. 2017 Jan 4;7:2125. doi: 10.3389/fmicb.2016.02125 (PMC5209349; doi:10.3389/fmicb.2016.02125)
Supplement: Supplementary file 9 [file DataSheet4.ZIP › Supplemental_File_4_AviaryBarn_EnviroAgeGrp_TaxaSummaries/charts/YSkdFRr3byBZrolNmeFqSo937WHy9O_legend.pdf]

NOHIT;Other;Other;Other;Other
k__Archaea;p__Crenarchaeota;c__Thaumarchaeota;o__Cenarchaeales;f__Cenarchaeaceae
k__Bacteria;p__Acidobacteria;c__BPC102;o__MVS-40;f__
k__Bacteria;p__Acidobacteria;c__DA052;o__Ellin6513;f__
k__Bacteria;p__Actinobacteria;c__Acidimicrobiia;o__Acidimicrobiales;f__OCS155
k__Bacteria;p__Actinobacteria;c__Actinobacteria;o__Actinomycetales;f__
k__Bacteria;p__Actinobacteria;c__Actinobacteria;o__Actinomycetales;f__Actinomycetaceae
k__Bacteria;p__Actinobacteria;c__Actinobacteria;o__Actinomycetales;f__Brevibacteriaceae
k__Bacteria;p__Actinobacteria;c__Actinobacteria;o__Actinomycetales;f__Corynebacteriaceae
k__Bacteria;p__Actinobacteria;c__Actinobacteria;o__Actinomycetales;f__Dermabacteraceae
k__Bacteria;p__Actinobacteria;c__Actinobacteria;o__Actinomycetales;f__Dietziaceae
k__Bacteria;p__Actinobacteria;c__Actinobacteria;o__Actinomycetales;f__Intrasporangiaceae
k__Bacteria;p__Actinobacteria;c__Actinobacteria;o__Actinomycetales;f__Jonesiaceae
k__Bacteria;p__Actinobacteria;c__Actinobacteria;o__Actinomycetales;f__Kineosporiaceae
k__Bacteria;p__Actinobacteria;c__Actinobacteria;o__Actinomycetales;f__Microbacteriaceae
k__Bacteria;p__Actinobacteria;c__Actinobacteria;o__Actinomycetales;f__Micrococcaceae
k__Bacteria;p__Actinobacteria;c__Actinobacteria;o__Actinomycetales;f__Mycobacteriaceae
k__Bacteria;p__Actinobacteria;c__Actinobacteria;o__Bifidobacteriales;f__Bifidobacteriaceae
k__Bacteria;p__Actinobacteria;c__Coriobacteriia;o__Coriobacteriales;f__Coriobacteriaceae
k__Bacteria;p__Bacteroidetes;c__Bacteroidia;o__Bacteroidales;Other
k__Bacteria;p__Bacteroidetes;c__Bacteroidia;o__Bacteroidales;f__Bacteroidaceae
k__Bacteria;p__Bacteroidetes;c__Bacteroidia;o__Bacteroidales;f__Porphyromonadaceae
k__Bacteria;p__Bacteroidetes;c__Bacteroidia;o__Bacteroidales;f__Prevotellaceae
k__Bacteria;p__Bacteroidetes;c__Bacteroidia;o__Bacteroidales;f__Rikenellaceae
k__Bacteria;p__Bacteroidetes;c__Bacteroidia;o__Bacteroidales;f__S24-7
k__Bacteria;p__Bacteroidetes;c__Bacteroidia;o__Bacteroidales;f__[Barnesiellaceae]
k__Bacteria;p__Bacteroidetes;c__Bacteroidia;o__Bacteroidales;f__[Odoribacteraceae]
k__Bacteria;p__Bacteroidetes;c__Bacteroidia;o__Bacteroidales;f__[Paraprevotellaceae]
k__Bacteria;p__Bacteroidetes;c__Flavobacteriia;o__Flavobacteriales;f__Flavobacteriaceae
k__Bacteria;p__Bacteroidetes;c__Flavobacteriia;o__Flavobacteriales;f__[Weeksellaceae]
k__Bacteria;p__Bacteroidetes;c__Sphingobacteriia;o__Sphingobacteriales;f__Sphingobacteriaceae
k__Bacteria;p__Bacteroidetes;c__[Saprospirae];o__[Saprospirales];f__Chitinophagaceae
k__Bacteria;p__Bacteroidetes;c__[Saprospirae];o__[Saprospirales];f__Saprospiraceae
k__Bacteria;p__Chlorobi;c__OPB56;o__;f__
k__Bacteria;p__Chloroflexi;c__Anaerolineae;o__GCA004;f__
k__Bacteria;p__Chloroflexi;c__Anaerolineae;o__SBR1031;f__SHA-31
k__Bacteria;p__Cyanobacteria;c__4C0d-2;o__MLE1-12;f__
k__Bacteria;p__Cyanobacteria;c__Synechococcophycideae;o__Synechococcales;f__Synechococcaceae
k__Bacteria;p__Deferribacteres;c__Deferribacteres;o__Deferribacterales;f__Deferribacteraceae
k__Bacteria;p__Firmicutes;c__Bacilli;o__Bacillales;Other
k__Bacteria;p__Firmicutes;c__Bacilli;o__Bacillales;f__
k__Bacteria;p__Firmicutes;c__Bacilli;o__Bacillales;f__Alicyclobacillaceae
k__Bacteria;p__Firmicutes;c__Bacilli;o__Bacillales;f__Bacillaceae
k__Bacteria;p__Firmicutes;c__Bacilli;o__Bacillales;f__Planococcaceae
k__Bacteria;p__Firmicutes;c__Bacilli;o__Bacillales;f__Staphylococcaceae
k__Bacteria;p__Firmicutes;c__Bacilli;o__Lactobacillales;Other
k__Bacteria;p__Firmicutes;c__Bacilli;o__Lactobacillales;f__
k__Bacteria;p__Firmicutes;c__Bacilli;o__Lactobacillales;f__Aerococcaceae
k__Bacteria;p__Firmicutes;c__Bacilli;o__Lactobacillales;f__Carnobacteriaceae
k__Bacteria;p__Firmicutes;c__Bacilli;o__Lactobacillales;f__Enterococcaceae
k__Bacteria;p__Firmicutes;c__Bacilli;o__Lactobacillales;f__Lactobacillaceae
k__Bacteria;p__Firmicutes;c__Bacilli;o__Lactobacillales;f__Leuconostocaceae
k__Bacteria;p__Firmicutes;c__Bacilli;o__Lactobacillales;f__Streptococcaceae
k__Bacteria;p__Firmicutes;c__Bacilli;o__Turicibacterales;f__Turicibacteraceae
k__Bacteria;p__Firmicutes;c__Clostridia;o__Clostridiales;Other
k__Bacteria;p__Firmicutes;c__Clostridia;o__Clostridiales;f__
k__Bacteria;p__Firmicutes;c__Clostridia;o__Clostridiales;f__Clostridiaceae
k__Bacteria;p__Firmicutes;c__Clostridia;o__Clostridiales;f__Eubacteriaceae
k__Bacteria;p__Firmicutes;c__Clostridia;o__Clostridiales;f__Lachnospiraceae
k__Bacteria;p__Firmicutes;c__Clostridia;o__Clostridiales;f__Peptococcaceae
k__Bacteria;p__Firmicutes;c__Clostridia;o__Clostridiales;f__Peptostreptococcaceae
k__Bacteria;p__Firmicutes;c__Clostridia;o__Clostridiales;f__Ruminococcaceae
k__Bacteria;p__Firmicutes;c__Clostridia;o__Clostridiales;f__Veillonellaceae
k__Bacteria;p__Firmicutes;c__Clostridia;o__Clostridiales;f__[Tissierellaceae]
k__Bacteria;p__Firmicutes;c__Erysipelotrichi;o__Erysipelotrichales;f__Erysipelotrichaceae
k__Bacteria;p__Fusobacteria;c__Fusobacteriia;o__Fusobacteriales;f__Fusobacteriaceae
k__Bacteria;p__Gemmatimonadetes;c__Gemmatimonadetes;o__;f__
k__Bacteria;p__Planctomycetes;c__Planctomycetia;o__Gemmatales;f__Gemmataceae
k__Bacteria;p__Proteobacteria;c__Alphaproteobacteria;o__Caulobacterales;f__Caulobacteraceae
k__Bacteria;p__Proteobacteria;c__Alphaproteobacteria;o__Rhizobiales;f__
k__Bacteria;p__Proteobacteria;c__Alphaproteobacteria;o__Rhizobiales;f__Bradyrhizobiaceae
k__Bacteria;p__Proteobacteria;c__Alphaproteobacteria;o__Rhizobiales;f__Brucellaceae
k__Bacteria;p__Proteobacteria;c__Alphaproteobacteria;o__Rhizobiales;f__Hyphomicrobiaceae
k__Bacteria;p__Proteobacteria;c__Alphaproteobacteria;o__Rhizobiales;f__Methylobacteriaceae
k__Bacteria;p__Proteobacteria;c__Alphaproteobacteria;o__Rhizobiales;f__Phyllobacteriaceae
k__Bacteria;p__Proteobacteria;c__Alphaproteobacteria;o__Rhizobiales;f__Rhizobiaceae
k__Bacteria;p__Proteobacteria;c__Alphaproteobacteria;o__Rhodobacterales;f__Rhodobacteraceae
k__Bacteria;p__Proteobacteria;c__Alphaproteobacteria;o__Rhodospirillales;f__Acetobacteraceae
k__Bacteria;p__Proteobacteria;c__Alphaproteobacteria;o__Rhodospirillales;f__Rhodospirillaceae
k__Bacteria;p__Proteobacteria;c__Alphaproteobacteria;o__Rickettsiales;f__
k__Bacteria;p__Proteobacteria;c__Alphaproteobacteria;o__Rickettsiales;f__Pelagibacteraceae
k__Bacteria;p__Proteobacteria;c__Alphaproteobacteria;o__Rickettsiales;f__Rickettsiaceae
k__Bacteria;p__Proteobacteria;c__Alphaproteobacteria;o__Sphingomonadales;f__Erythrobacteraceae
k__Bacteria;p__Proteobacteria;c__Alphaproteobacteria;o__Sphingomonadales;f__Sphingomonadaceae
k__Bacteria;p__Proteobacteria;c__Betaproteobacteria;o__Burkholderiales;f__
k__Bacteria;p__Proteobacteria;c__Betaproteobacteria;o__Burkholderiales;f__Alcaligenaceae
k__Bacteria;p__Proteobacteria;c__Betaproteobacteria;o__Burkholderiales;f__Comamonadaceae
k__Bacteria;p__Proteobacteria;c__Betaproteobacteria;o__Burkholderiales;f__Oxalobacteraceae
k__Bacteria;p__Proteobacteria;c__Betaproteobacteria;o__Ellin6067;f__
k__Bacteria;p__Proteobacteria;c__Betaproteobacteria;o__IS-44;f__
k__Bacteria;p__Proteobacteria;c__Betaproteobacteria;o__MKC10;f__
k__Bacteria;p__Proteobacteria;c__Betaproteobacteria;o__Methylophilales;f__Methylophilaceae
k__Bacteria;p__Proteobacteria;c__Betaproteobacteria;o__Neisseriales;f__Neisseriaceae
k__Bacteria;p__Proteobacteria;c__Betaproteobacteria;o__Nitrosomonadales;f__Nitrosomonadaceae
k__Bacteria;p__Proteobacteria;c__Betaproteobacteria;o__Rhodocyclales;f__Rhodocyclaceae
k__Bacteria;p__Proteobacteria;c__Deltaproteobacteria;o__Desulfovibrionales;f__Desulfovibrionaceae
k__Bacteria;p__Proteobacteria;c__Deltaproteobacteria;o__Myxococcales;f__0319-6G20
k__Bacteria;p__Proteobacteria;c__Deltaproteobacteria;o__Sva0853;f__SAR324
k__Bacteria;p__Proteobacteria;c__Epsilonproteobacteria;o__Campylobacterales;f__Campylobacteraceae
k__Bacteria;p__Proteobacteria;c__Epsilonproteobacteria;o__Campylobacterales;f__Helicobacteraceae
k__Bacteria;p__Proteobacteria;c__Gammaproteobacteria;Other;Other
k__Bacteria;p__Proteobacteria;c__Gammaproteobacteria;o__Aeromonadales;f__Aeromonadaceae
k__Bacteria;p__Proteobacteria;c__Gammaproteobacteria;o__Alteromonadales;f__Alteromonadaceae
k__Bacteria;p__Proteobacteria;c__Gammaproteobacteria;o__Alteromonadales;f__Idiomarinaceae
k__Bacteria;p__Proteobacteria;c__Gammaproteobacteria;o__Alteromonadales;f__Shewanellaceae
k__Bacteria;p__Proteobacteria;c__Gammaproteobacteria;o__Alteromonadales;f__[Chromatiaceae]
k__Bacteria;p__Proteobacteria;c__Gammaproteobacteria;o__Enterobacteriales;f__Enterobacteriaceae
k__Bacteria;p__Proteobacteria;c__Gammaproteobacteria;o__Legionellales;f__Coxiellaceae
k__Bacteria;p__Proteobacteria;c__Gammaproteobacteria;o__Methylococcales;f__Methylococcaceae
k__Bacteria;p__Proteobacteria;c__Gammaproteobacteria;o__Pasteurellales;f__Pasteurellaceae
k__Bacteria;p__Proteobacteria;c__Gammaproteobacteria;o__Pseudomonadales;f__Moraxellaceae
k__Bacteria;p__Proteobacteria;c__Gammaproteobacteria;o__Pseudomonadales;f__Pseudomonadaceae
k__Bacteria;p__Proteobacteria;c__Gammaproteobacteria;o__Vibrionales;Other
k__Bacteria;p__Proteobacteria;c__Gammaproteobacteria;o__Vibrionales;f__Pseudoalteromonadaceae
k__Bacteria;p__Proteobacteria;c__Gammaproteobacteria;o__Vibrionales;f__Vibrionaceae
k__Bacteria;p__Proteobacteria;c__Gammaproteobacteria;o__Xanthomonadales;f__Sinobacteraceae
k__Bacteria;p__Proteobacteria;c__Gammaproteobacteria;o__Xanthomonadales;f__Xanthomonadaceae
k__Bacteria;p__Tenericutes;c__Mollicutes;o__RF39;f__
