# Supplementary figures and images for: Characterization of Gut Microbiome Dynamics in Developing Pekin Ducks and Impact of Management System (part 2 of 2)
Source: Front Microbiol. 2017 Jan 4;7:2125. doi: 10.3389/fmicb.2016.02125 (PMC5209349; doi:10.3389/fmicb.2016.02125)

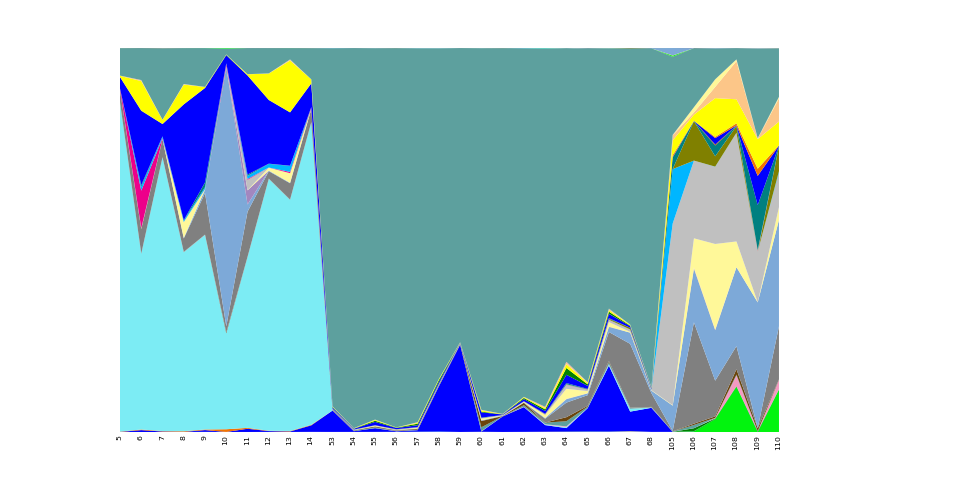

Supplement: Supplementary file 10 [file DataSheet5.ZIP › Supplemental_File_4_AviaryStudies_Days1-8_TaxaSummaries/charts/ZkoN2YhiRQc6Gn1YergrHBCBXDuAUU.png]
