# Supplementary material for: Characterization of Gut Microbiome Dynamics in Developing Pekin Ducks and Impact of Management System
Source: Front Microbiol. 2017 Jan 4;7:2125. doi: 10.3389/fmicb.2016.02125 (PMC5209349; doi:10.3389/fmicb.2016.02125)
Supplement: Supplementary file 11 [file DataSheet6.PDF]

frontiers

Supplementary Material

Characterization of Gut Microbiome Dynamics in Developing Pekin Ducks and Impact of Management System

Aaron A. Best*, Amanda L. Porter, Susan M. Fraley, Gregory S. Fraley

*** Correspondence:** Aaron A. Best: best@hope.edu

1 Supplementary Figures and Tables

1.1 Supplementary Figures

Supplementary Figure 1 Alpha diversity metrics for Aviary Studies 1 and 2. Samples were grouped by age of the duck throughout the grow out period. Four measures of alpha diversity (within sample variation) are shown. Analyses were conducted on a data rarefied to 10,000 sequencing reads. The average diversity and standard deviation are shown for each metric. Group level ANOVA results are described below. Significantly different pairs of age groups are described in the text and in Table S2. (A) Alpha diversity metrics through the 36 day grow out period for Aviary Study 1**.** Group level ANOVA results: Phylogenetic Distance, F=81.254, DF=5, p = 3.8539 x 10^{-24}; Chao1, F=27.546, DF=5, p = 7.5581 x 10^{-14}; Observed Species, F=89.165, DF=5, p=4.0731 x 10^{-25}; Shannon, F=74.226, DF=5, p=3.351 x 10^{-23}. In general, diversity of the gut microbiome increases as ducks mature through the grow out cycle. (B) Alpha diversity metrics through the first 10 days of the grow out period for Aviary Study 2. Group level ANOVA results: Phylogenetic Distance, F=2.813, DF=9, p = 0.009386; Chao1, F=1.223, DF=9, p = 0.302424; Observed Species, F=2.395, DF=9, p=0.024203; Shannon, F=20.318, DF=9, p=7.9934 x 10^{-14}. Shannon diversity of the gut microbiome increases as ducks mature through the 10 day period.

Supplementary Figure 2 Principal Coordinate Analysis of Weighted UniFrac Distances for Aviary Study 2. Samples are colored by age of the duck throughout the grow out period – Red, Day1; Blue, Day 2; Orange, Day 3; Green, Day 4; Purple, Day 5; Yellow, Day 6; Cyan, Day 7; Pink, Day 8; Dark Aqua, Day 9; Brown, Day 10. Analyses were conducted on data rarefied to 10,000 sequencing reads. Axes are scaled by the percent of variation explained by each principle coordinate. Individual ceacal samples from ducks of the same age tend to group along PC1. Halos from statistical

resampling are not visible at this scale, an indication of the significance of the separation shown between points.

Supplementary Figure 3 Comparison of Aviary Studies Days 1 and 8. (A) Principal Coordinate Analysis of Weighted UniFrac Distances for Aviary Studies, Days 1 and 8. Samples are colored by age of the duck and study – Red, Day 1-Study 1; Blue, Day 1-Study 2; Orange, Day 8-Study 1; Green, Day 8-Study 2. Analyses were conducted on data rarefied to 10,000 sequencing reads. Axes represent the percent of variation explained by each principle coordinate. Individual ceacal samples from one day old ducks group along both PC1 and PC2, whereas samples from eight day old ducks are distinct from Day 1 ducks (along PC1) and from each other (both PC1 and PC2). (B) Summary of Bacterial Taxa Observed for Aviary Studies, Days 1 and 8. The relative abundances of bacterial 97% operational taxonomic units (OTUs) are shown for duck caecal samples from days 1 and 8 of Aviary Studies 1 and 2. Depicts genus level (or higher) classifications for observed OTUs for samples grouped according to the age of the duck. Full color legends for each panel are listed in Supplementary Table S5, Taxa Summary Legends. The relative abundance of taxa is similar between samples from day 1 ducks, whereas samples from day 8 ducks differ greatly between the two studies.

1.2 Supplementary Tables

Supplementary Table 1 This table includes information for each sequenced sample analyzed in this study. The text version of the table through the description column served as the mapping file for all Qiime analyses. The table also includes the number of sequencing reads for each sample after quality filtering and alpha diversity metrics for each sample (not rarefied).

Supplementary Table 2 This table contains pairwise t-test results for alpha diversity metrics calculated on 10,000 rarefied reads per sample for Aviary Study 1 grouped by age of the ducks.

Supplementary Table 3 This table contains the results of DESeq2 analyses for different groups of samples. Each sheet contains the results of a pairwise DESeq2 analysis from 1) duck samples in Aviary Study 1 at each of the developmental time points (1, 8, 15, 22, 29 and 36 days) and 2) duck samples in Aviary Studies 1 and 2 compared for Days 1 and 8. Taxa that are significantly differentially distributed at a p-value of <= 0.05 are highlighted in gray. This set is sorted on the log fold change (least to greatest). The directionality of the fold change is associated with one of the two comparison groups as indicated in the "Enriched in" column labeled by day or aviary study and day. The sum of the number of taxa observed as significantly differentially distributed for the groups in the comparison are indicated in the "Totals" column. A breakdown of the number of taxa associated with phylum level groupings is also shown.

Supplementary Table 4 This table contains the results of DESeq2 analyses for different groups of samples. Each sheet contains the results of a pairwise DESeq2 analysis from duck samples in Aviary and Barn studies compared for environment and a grouping that combines environment plus three age groups (early, mid, late). Taxa that are significantly differentially distributed at a p-value of <= 0.05 are highlighted in gray. This set is sorted on the log fold change (least to greatest). The directionality of the fold change is associated with one of the two comparison groups as indicated in the "Enriched in" column labeled by environment or environmental age group. The sum of the number of taxa observed as significantly differentially distributed for the groups in the comparison are indicated in the "Totals" column. A breakdown of the number of taxa associated with phylum level groupings is also shown.

Supplementary Table 5 This table contains the full color legends for taxa summaries depicted in Figures 2, 3, 4, 5 and Supplementary Figure 3. In addition, the relative proportion of each taxon for each sample or sample group are included.

Supplementary File 1 This is a compressed directory containing output from a taxa summary analysis in Qiime. The directory contains individual sample taxa summaries for Aviary Study 1 (supplements Figure 2). To examine the results, uncompress the directory and open the file, "bar_charts.html", in a browser. Bar charts of relative abundances of taxa for each sample, full color legends, and relative abundance tables are shown for all phylogenetic levels. Mousing over a color in an individual bar shows taxon and relative abundance. Sample IDs can be cross referenced with Supplemental Table 1 to determine the features of each individual sample.

Supplementary File 2 This is a compressed directory containing output from a taxa summary analysis in Qiime. The directory contains individual sample taxa summaries for Aviary Study 2 (supplements Figure 3). To examine the results, uncompress the directory and open the file, "bar_charts.html", in a browser. Bar charts of relative abundances of taxa for each sample, full color legends, and relative abundance tables are shown for all phylogenetic levels. Mousing over a color in an individual bar shows taxon and relative abundance. Sample IDs can be cross referenced with Supplemental Table 1 to determine the features of each individual sample.

Supplementary File 3 This is a compressed directory containing output from a taxa summary analysis in Qiime. The directory contains individual sample taxa summaries for the comparison of Aviary and Barn environments (Supplements Figure 4). To examine the results, uncompress the directory and open the file, "bar_charts.html", in a browser. Bar charts of relative abundances of taxa for each sample, full color legends, and relative abundance tables are shown for all phylogenetic levels. Mousing over a color in an individual bar shows taxon and relative abundance. Sample IDs can be cross referenced with Supplemental Table 1 to determine the features of each individual sample.

Supplementary File 4 This is a compressed directory containing output from a taxa summary analysis in Qiime. The directory contains individual sample taxa summaries for the comparison of Aviary and Barn environments grouped into age category (early, mid, late) (Supplements Figure 5). To examine the results, uncompress the directory and open the file, "bar_charts.html", in a browser. Bar charts of relative abundances of taxa for each sample, full color legends, and relative abundance tables are shown for all phylogenetic levels. Mousing over a color in an individual bar shows taxon and relative abundance. Sample IDs can be cross referenced with Supplemental Table 1 to determine the features of each individual sample.

Supplementary File 5 This is a compressed directory containing output from a taxa summary analysis in Qiime. The directory contains individual sample taxa summaries for Aviary Study 1 and Aviary Study 2 ducks aged 1 and 8 days (Supplements Supplemental Figure 3). To examine the results, uncompress the directory and open the file, "bar_charts.html", in a browser. Bar charts of relative abundances of taxa for each sample, full color legends, and relative abundance tables are shown for all phylogenetic levels. Mousing over a color in an individual bar shows taxon and relative abundance. Sample IDs can be cross referenced with Supplemental Table 1 to determine the features of each individual sample.
