# Supplementary material for: Characterization of Gut Microbiome Dynamics in Developing Pekin Ducks and Impact of Management System
Source: Front Microbiol. 2017 Jan 4;7:2125. doi: 10.3389/fmicb.2016.02125 (PMC5209349; doi:10.3389/fmicb.2016.02125)
Supplement: Supplementary file 13 [file Image2.PDF]

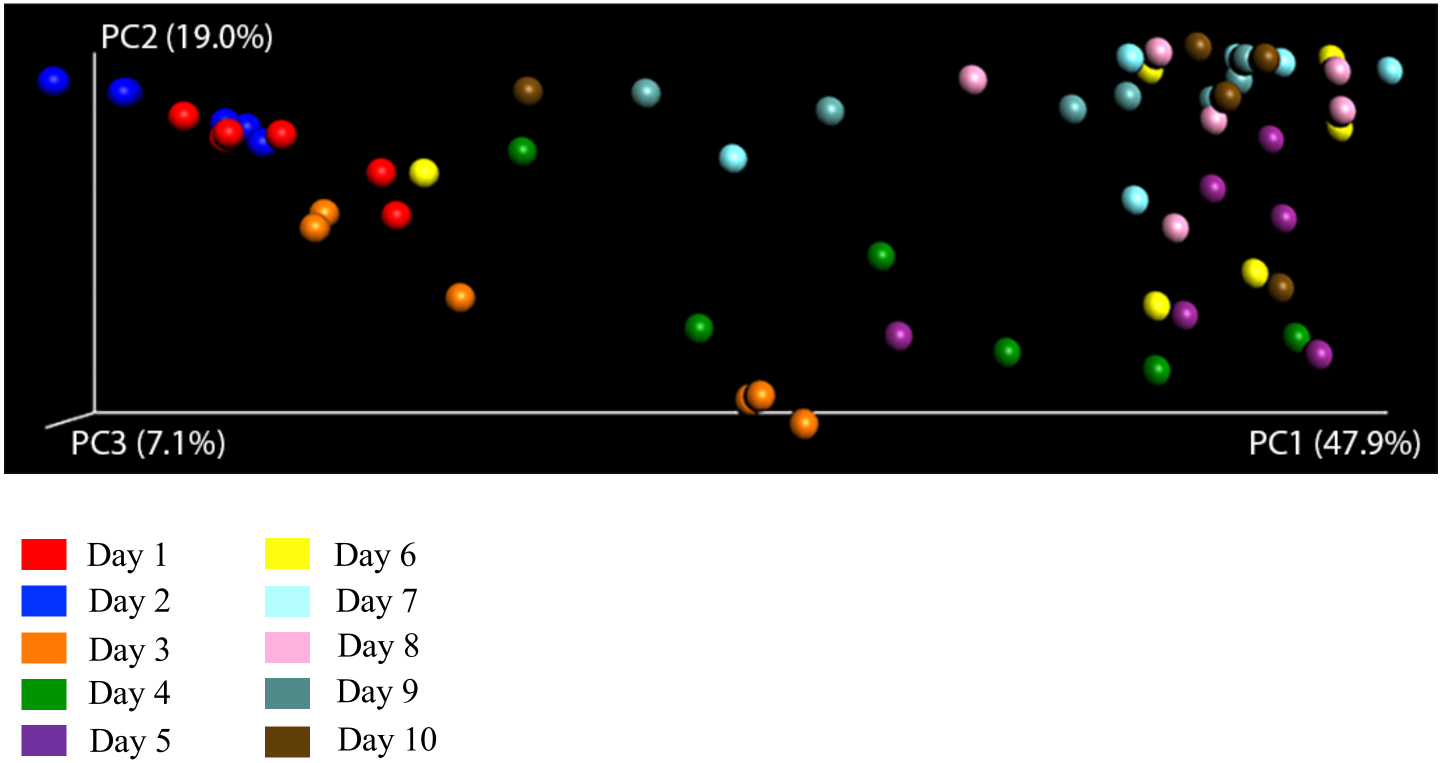

Supplementary Figure 2 Principal Coordinate Analysis of Weighted UniFrac Distances for Aviary Study 2. Samples are colored by age of the duck throughout the grow out period – Red, Day1; Blue, Day 2; Orange, Day 3; Green, Day 4; Purple, Day 5; Yellow, Day 6; Cyan, Day 7; Pink, Day 8; Dark Aqua, Day 9; Brown, Day 10. Analyses were conducted on data rarefied to 10,000 sequencing reads. Axes are scaled by the percent of variation explained by each principle coordinate. Individual ceacal samples from ducks of the same age tend to group along PC1. Halos from statistical resampling are not visible at this scale, an indication of the significance of the separation shown between points.
