# Supplementary material for: Characterization of Gut Microbiome Dynamics in Developing Pekin Ducks and Impact of Management System
Source: Front Microbiol. 2017 Jan 4;7:2125. doi: 10.3389/fmicb.2016.02125 (PMC5209349; doi:10.3389/fmicb.2016.02125)
Supplement: Supplementary file 14 [file Image3.PDF]

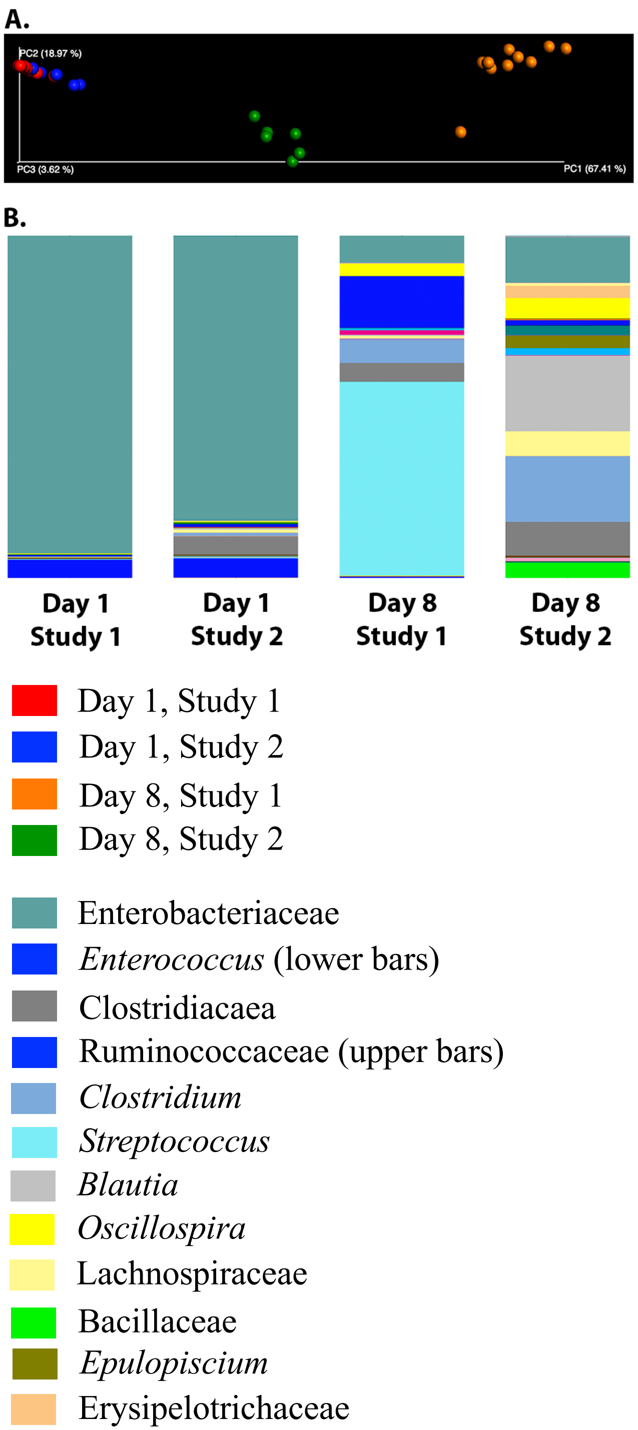

Supplementary Figure 3 Comparison of Aviary Studies Days 1 and 8. (A) Principal Coordinate Analysis of Weighted UniFrac Distances for Aviary Studies, Days 1 and 8. Samples are colored by age of the duck and study – Red, Day 1-Study 1; Blue, Day 1-Study 2; Orange, Day 8-Study 1; Green, Day 8-Study 2. Analyses were conducted on data rarefied to 10,000 sequencing reads. Axes represent the percent of variation explained by each principle coordinate. Individual ceacal samples from one day old ducks group along both PC1 and PC2, whereas samples from eight day old ducks are distinct from Day 1 ducks (along PC1) and from each other (both PC1 and PC2). (B) Summary of Bacterial Taxa Observed for Aviary Studies, Days 1 and 8. The relative abundances of bacterial 97% operational taxonomic units (OTUs) are shown for duck caecal samples from days 1 and 8 of Aviary Studies 1 and 2. Depicts genus level (or higher) classifications for observed OTUs for samples grouped according to the age of the duck. Full color legends for each panel are listed in Supplementary Table S5, Taxa Summary Legends. The relative abundance of taxa is similar between samples from day 1 ducks, whereas samples from day 8 ducks differ greatly between the two studies.
